# Reductive Amination of Dialdehyde Cellulose: Access to Renewable Thermoplastics

**DOI:** 10.1021/acs.biomac.2c01022

**Published:** 2022-12-21

**Authors:** Jonas Simon, Lukas Fliri, Janak Sapkota, Matti Ristolainen, Stephen A. Miller, Michael Hummel, Thomas Rosenau, Antje Potthast

**Affiliations:** † Department of Chemistry, Institute of Chemistry of Renewable Resources, University of Natural Resources and Life Sciences Vienna (BOKU), Konrad-Lorenz-Strasse 24, Tulln3430, Austria; ‡ Department of Bioproducts and Biosystems, Aalto University, Aalto0076, Finland; § NE Research Center, UPM Pulp Research and Innovations, Lappeenranta53200, Finland; ∥ The George and Josephine Butler Laboratory for Polymer Research, Department of Chemistry, University of Florida, Gainesville, Florida32611-7200, United States

## Abstract

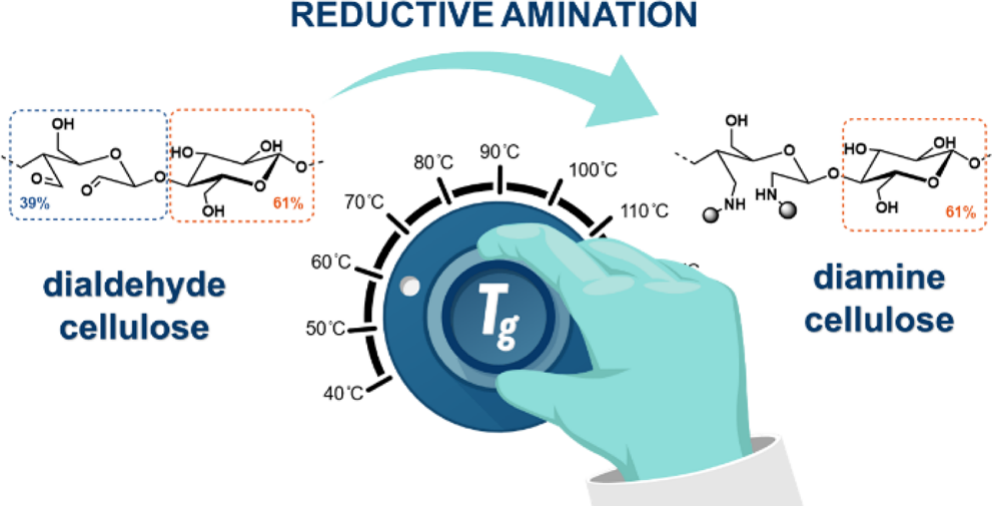

The reductive amination of dialdehyde cellulose (DAC) with 2-picoline borane was investigated for its applicability in the generation of bioderived thermoplastics. Five primary amines, both aliphatic and aromatic, were introduced to the cellulose backbone. The influences of the side chains on the course of the reaction were examined by various analytical techniques with microcrystalline cellulose as a model compound. The obtained insights were transferred to a 39%-oxidized softwood kraft pulp to study the thermal properties of thereby generated high-molecular-weight thermoplastics. The number-average molecular weights (*M*_n_) of the diamine celluloses, ranging from 60 to 82 kD, were investigated by gel permeation chromatography. The diamine celluloses exhibited glass transition temperatures (*T*_g_) from 71 to 112 °C and were stable at high temperatures. Diamine cellulose generated from aniline and DAC showed the highest conversion, the highest *T*_g_ (112 °C), and a narrow molecular weight distribution (*D̵* of 1.30).

## 1 Introduction

Our modern society relies on fossil fuel-based plastics and enjoys the comfort of single-use plastics in daily life. However, the world is now facing the consequences of constantly increasing plastic pollution.^1^ An effective way to tackle this issue and to deal with the depletion of fossil fuels^2^ is to meet it head-on, by replacing all commodity plastics with sustainable bioderived alternatives. In addition to low production costs, such bioplastics must have similar or even improved thermal and mechanical properties compared to today’s commodity plastics.^3^ Thermal properties define the range of application and processability. Thus, high glass transition temperatures (*T*_g_) are needed—preferably above 100 °C to provide high mechanical strength also when, for instance, in contact with hot beverages.^4^ Cellulose theoretically provides two structural features by nature required to generate high *T*_g_ bioplastics: high conformational barriers due to the repeating unit ring structure and strong interchain interactions through strong hydrogen bonding and some hydrophobic interactions.^5,6^ However, the energy of the hydrogen bonding and hydrophobic interactions in native cellulose exceeds the degradation energy, making native cellulose thermally unprocessable, that is, unmeltable.^7,8^ The overarching strategies to plasticize cellulose are twofold: (1) derivatization of the hydroxy groups in C2, C3, and C6 position to cellulose graft copolymers to decrease intermolecular strength while maintaining the polymer backbone^9−16^ or (2) partial cleavage of the cellulose main-chain followed by introducing side chains into the generated “soft” segments.^17−22^

Many polymer scientists have studied cellulose derivatives or cellulose graft copolymers in the past century by modifying the hydroxy groups in cellulose, including cellulose acetate or cellulose acetate butyrate, both commercialized in the late 1800s.^9,10^ However, melt processing of these short-chain cellulose esters relies on adding large amounts of plasticizers.^23−25^ The addition of external plasticizers compromises their performance, recyclability, and compatibility with human health and the environment.^26−28^ Introducing long side chains along the cellulose backbone circumvents the need for external plasticizers and improves thermal processability.^11−16^ Although improving thermal processability, derivatization with long-chain fatty acids or other bulky reactants necessitates vigorous reaction conditions. These include high reaction temperatures, long reaction times, hazardous solvents, or toxic reagents. In addition, high degrees of modification are required to generate cellulose-based thermoplastics, which can decrease the thermal stability in turn.^16^

The second strategy to modify cellulose into thermally processable derivatives is by partially cleaving the cellulose backbone. Generally, this approach generates a considerable fraction of “soft” segments, which adds flexibility and weakens the strong interchain interactions. Periodate oxidation of cellulose cleaves the cellulose backbone under mild conditions selectively between the C2/C3 positions of the glucopyranose units, thereby generating dialdehyde cellulose (DAC).^29,30^ The periodate reactivity can be further increased by adding metal salts^31,32^ and by mechanical^33^ or ultrasound treatment.^34^ A major drawback of periodate oxidation is the generation of equimolar amounts of toxic waste^35^ and the high price and toxicity of sodium periodate itself.^36,37^ Nevertheless, these problems can be compensated by efficient recycling of the oxidant either electrochemically^38,39^ or with hypochlorite.^37^ Being environmentally most compatible, an ozone treatment under alkaline conditions^40^ can be employed for periodate recycling, which is easily upscalable and has the additional advantage of removing low-molecular-weight organic byproducts. Plappert et al. previously published the successful formation of transparent DAC films with high oxygen barrier properties and demonstrated the potential of DAC for film applications.^41^ However, unmodified DAC shows no thermoplastic behavior prior to decomposition, limiting its use to solvent-based film casting. The non-thermoplastic behavior of DAC, despite the open ring of its “formal” dialdehyde structure, is presumably caused by interchain cross-linking of the aldehyde groups in the form of hemialdals and hemiacetals.^42−44^ Furthermore, the inherent instability of native DAC toward aging,^100^ discoloration, and beta-elimination^45^ likewise introduces problems for packaging applications. If the aldehyde groups and their masked forms are further transformed, the unwarranted cross-links can be cleaved and, depending on the introduced modification, the materials’ stability can be improved. This offers access to thermoplastics with attractive thermal and mechanical properties. For example, after reduction with sodium borohydride, the generated dialcohol celluloses showed *T*_g_ values above 75 °C.^17−21^ In 2020, Esen and Meier reported another DAC modification via the Passerini three-component reaction, yielding a series of DAC-based products with very high *T*_g_ values (121 to 166 °C).^22^ The *T*_g_ decreased for both DAC derivatives (dialcohol cellulose and Passerini products) with an increasing number of cleaved glucopyranose units, the derivatives being already thermoplastic when starting from partially oxidized cellulose.^17,22^ The thermal data of the dialcohol celluloses and the Passerini products demonstrated that the *T*_g_ in DAC derivatives can be controlled in two ways: (1) by adjusting the rigidity of the cellulose backbone through the number of cleaved glucopyranose units, often referred to as the degree of oxidation (DO), and (2) by introducing different side chains.

The reactive aldehyde functionality of DAC offers various modification strategies including imine formation, oxidation, reduction, oximation, or reductive amination. Nonetheless, to the best of our knowledge, beyond sodium borohydride reduction and Passerini reaction, none of the materials derived from DAC have so far been investigated toward their suitability and performance as thermoplastics with tunable thermal properties. Considering the vast variety of commercially available amines, we decided to investigate reductive aminations of DAC as a potential pathway to generate thermoplastic cellulose materials via DAC as an intermediate.

**Scheme 1:**
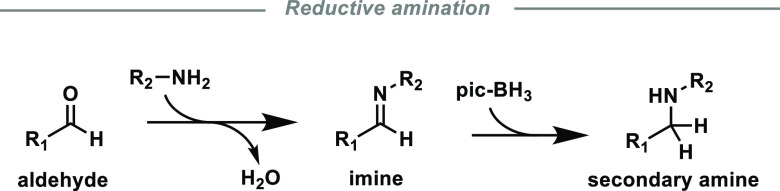
Reductive Amination of Aldehydes with a Primary Amine and 2-Picoline Borane (pic-BH_3_)

Previous studies modified DAC through reductive amination, for example, to immobilize proteins,^46−48^ to analyze the topochemistry of the surface oxidation of cellulose,^49^ to generate biobased absorbers,^50−54^ or to cast films.^55^ Thereby, different reaction conditions, reagents, and reducing agents, such as sodium borohydride, cyanoborohydride, or 2-picoline borane, were applied. Given the known depolymerization of DAC already at slight alkalinity, which is a major drawback of reductions with sodium borohydride, we used 2-picoline borane in this work (Scheme 1).^56,57^ The reduction with 2-picoline borane proceeds under slightly acidic reaction conditions, which holds beta-elimination side reactions^45,58^ at bay and increases the reactivity of DAC itself by shifting the equilibria between masked and free aldehyde groups toward the latter.^59^

Previous research on DAC-derived thermoplastics has been conducted with a variety of starting materials with a wide range of DOs. Esen and Meier, for example, used a water-soluble, high-DO fraction isolated from microcrystalline cellulose for their study. Although this approach allows for homogenous reaction conditions, simplifies work-up, and gives access to more powerful solution-state analytical procedures, it also necessitates harsher reaction conditions and excessive use of reagents during DAC formation. In a more industrially relevant context, we conducted our study with partially oxidized softwood kraft pulp. However, the heterogeneous reaction conditions caused new preparational and analytical challenges. Although the literature on the transformation of DAC is vast, little effort has been generally undertaken to confirm the chemistry of the derivatized polymers unambiguously. Often full conversion to the anticipated product is postulated based on lower-resolution spectroscopy tools, such as Fourier transform infrared (FTIR) or cross-polarization magic angle spinning ^13^C nuclear magnetic resonance (NMR) spectroscopy. However, these techniques can be incapable of detecting minor products from possible and probable side reactions, which likewise influence the materials’ properties. Furthermore, the assumption of quantitative transformation is disputable also for other reasons, for instance, the presence of relatively stable hemiacetal cross-links in the DAC educt, which results in decreased reactivity compared to the free aldehyde.

**Scheme 2:**
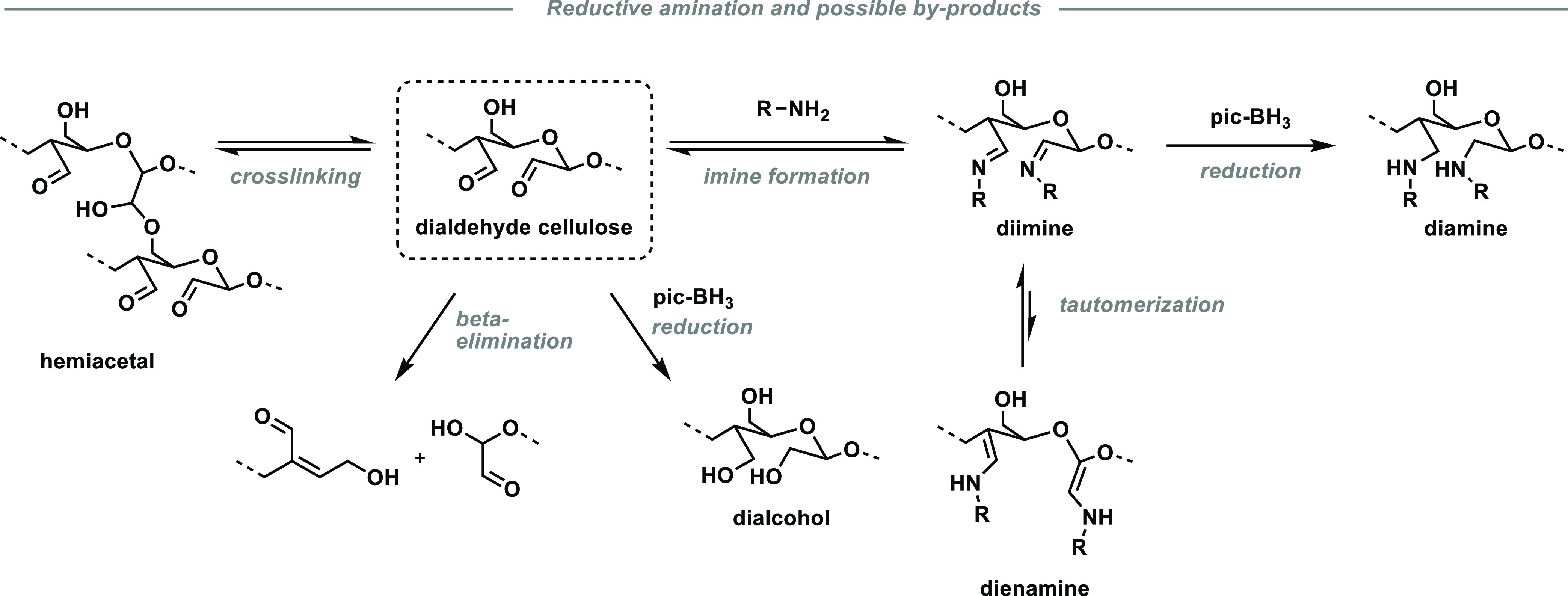
Reductive Amination of Dialdehyde Cellulose with a Primary Amine (R–NH_2_) and 2-Picoline Borane (pic-BH_3_): Main Process and Possible Side Reactions

In the case of the presented reductive aminations, several side reactions are conceivable, including reduction of the carbonyl groups to dialcohol cellulose, incomplete reduction of the corresponding imine, imine–enamine tautomerization, formation of seven-membered cyclic structures,^60,61^ alkali-induced degradation, and incomplete conversion or reformation of the dialdehyde moiety, resulting in residual hemiacetal cross-links (see Scheme 2). To establish the anticipated relationship of the introduced amine side chain on the thermal properties, exact knowledge of the functional groups present in the material is necessary. For this purpose, besides commonly applied FTIR spectroscopy, elemental analysis (EA), and titration techniques, we also used a recently reported solution-state NMR protocol for celluloses and cellulose derivatives, relying on [P_4444_][OAc]/DMSO-*d*_6_ (w/w = 1:4) as a solvent to characterize the obtained materials.^62,63^ To assist in the interpretation of the NMR data, the reactions were separately performed on a partly oxidized microcrystalline cellulose as a model without solubility issues and without peak superposition from hemicelluloses, before working with the industrial pulp. Furthermore, the impact of the reductive amination on the molecular weight distribution was investigated by gel permeation chromatography (GPC). The influence of the introduced moieties and the change in the molecular weight distribution on the thermal properties were investigated by thermogravimetric analysis (TGA) and differential scanning calorimetry (DSC).

## 2 Experimental Section

### 2.1 Chemicals and Reagents

Softwood kraft pulp (mixture of spruce and pine) was provided by UPM Kymmene Oyj (Lappeenranta, Finland). The hemicellulose content was 8.5% calculated from peak deconvolution of the C4 resonance in the ^13^C solid-state NMR spectrum (Table S1) according to Jusner et al.^64^ The calculated hemicellulose content was in good agreement with the monosaccharide quantification by methanolysis and gas chromatography (Table S2) according to Sundherg et al.^65,66^ The molecular weight was previously determined by multidetector GPC (Figure S3): *M*_n_ = 55 kDa, *M*_w_ = 614 kDa, *M*_z_ = 1651 kDa, and *D̵* = 11.1. The softwood kraft pulp was disintegrated in deionized water using a commercial kitchen blender before use. Avicel (microcrystalline cellulose) with a reported particle size of 50 μm (Sigma-Aldrich), sodium periodate (≥99.8%; Sigma-Aldrich), 2-picoline borane (95%; Sigma-Aldrich), tyramine (≥98.0%; TCI), ethanolamine (≥99.0%; Fluka Analytical), butylamine (99.5%; Sigma-Aldrich), hexylamine (99%; Acros Organics), and aniline (99%; Sigma-Aldrich) were purchased and used without further purification.

### 2.2 Characterization

Solution-state NMR experiments were performed according to a procedure initially reported for cellulose nanocrystals (CNCs).^62,63^ The used [P_4444_][OAc]/DMSO-*d*_6_ (w/w = 1:4) solvent was prepared according to the literature. All spectra were recorded on a Bruker NMR AV III 400 spectrometer at an acquisition temperature of 65 °C. For standard sample preparation, 50 mg of the freeze-dried cellulosic material was weighed into a 4 mL screw cap glass vial, before [P_4444_][OAc]/DMSO-*d*_6_ (w/w = 1:4) was added up to a final weight of 1.0 g, resulting in a concentration of 5 wt%. The sealed mixture was stirred with a small magnetic stirring bar and heated to 65°C by means of an oil bath for 16 to 20 h. Thereafter, the homogenous samples were transferred into standard 4 mm NMR tubes. NMR experiments were performed on the cellulosic starting materials, the obtained DAC, the five diamine celluloses derived from microcrystalline cellulose, two diamine celluloses derived from the softwood kraft pulp sample, and a dialcohol cellulose model compound for comparison. Owing to the high molecular weight of the softwood kraft pulp and resulting solubility issues, a lower measuring concentration of 1 wt% had to be used. All NMR samples were routinely characterized by quantitative ^1^H NMR (pulseprog: *zg*; ns = 32; d1 = 10s), diffusion-edited ^1^H NMR (pulseprog: *ledbpgp2s1d*; ns = 512; d1 = 1s),^63^ and 2D multiplicity-edited ^1^H–^13^C HSQC spectra (pulseprog: *hsqcedetgpsisp2.3*; ns = 8 with 512 f1 increments). The obtained spectra are summarized in the Supporting Information. Due to significant overlap of the peaks from the introduced moieties with the water peak and residual electrolyte or cellulose backbone resonances, no meaningful calculation of the degree of substitution (DS) could be performed based on the quantitative ^1^H data. However, semiquantitative evaluations of the qualitative diffusion-edited ^1^H spectra were carried out and were in good agreement with the DS determination according to EA and titration data. Owing to comparably low resolution, despite excessively long measurement times, no 1D ^13^C NMR spectra were recorded. The ^13^C resonances were extracted from the 2D spectra (HSQC) instead.

EA was performed by combustion EA on a Thermo Flash Smart CHNSO elemental analyzer. For sample preparation, 1–3 mg of the material was accurately weighed into tin foil cups. Cellulosic samples were thoroughly freeze-dried before measurement. The device was calibrated by a linear calibration using sulfanilamide as the standard. All measurements were carried out at least in triplicate and averaged. C, H, N, and S were directly analyzed, and DS calculations were based on the nitrogen values.

TGA was performed on a TGA 5500 (TA Instruments). About 5 to 10 mg of each sample was equilibrated at room temperature and heated at 10 °C min^–1^ to 600 °C under a nitrogen flow rate of 25 mL min^–1^. The reported *T*_95_ values represent the temperatures at which 5 % of the mass is lost, neglecting mass loss from solvent evaporation before 100 °C.

DSC thermograms were obtained using a DSC2500 apparatus (TA Instruments). About 15 mg of each sample was dried under nitrogen at 100 °C for 15 min. Aliquots of these samples (5 to 10 mg) were weighed into a sealed aluminum pan that passed through a heat–cool–heat cycle at 10 °C min^–1^. Reported data are from the second heating cycle. The temperature ranged from (min) −50 to (max) 190 °C.

FTIR spectra were obtained on a Frontier FTIR spectrophotometer from PerkinElmer operating in the attenuated total reflection (ATR) mode. The diamine cellulose samples were dried under reduced pressure before analysis. The parameters for all measurements included 4 cm^–1^ resolution, the 4000–650 cm^–1^ spectral range, and accumulation of 32 scans per sample. The obtained spectra are summarized in the Supporting Information.

Potentiometric measurements were performed using an 877 Titrino plus instrument from Metrohm AG equipped with a 30 mL beaker, a 20 mL dosing unit, and a magnetic stirrer.

GPC was performed using a size exclusion/multiangle light scattering (SEC-MALLS) system including a MALLS detector (Wyatt Dawn DSP, Wyatt Inc.) coupled with a refractive index detector (Shodex RI-71, Showa Denko K.K.), four Waters HPLC columns (Styrage HMW 6E, 7.8 mm i.d., 300 mm length, 15–20 μm), one Agilent GPC/SEC guard column (PL gel, 7.8 mm i.d., 50 mm length, 20 μm), and a Bio-Inert 1260 Infinity II pump (Agilent) with automatic injection (HP Series 1100 autosampler, Agilent). *N*,*N*-Dimethylacetamide/lithium chloride (0.9%, w/v; filtered through a 0.02 μm filter) was used as the mobile phase, and 100 μL was injected for each measurement with a 45 min run time at a flow rate of 1 mL/min. All samples were dissolved after the work-up procedure in *N*,*N*-dimethylacetamide/lithium chloride (9% w/v) according to the standard procedure for cellulose samples by Siller et al.^67,68^ Dissolved samples were diluted with *N*,*N*-dimethylacetamide and filtered through a 0.45 μm syringe filter before analysis. The molecular weight distribution and the GPC–MALLS statistical moments were calculated based on a refractive index increment of 0.136 mL/g for cellulose in *N*,*N*-dimethylacetamide/lithium chloride (0.9% w/v). The raw data were processed with Astra 4.7 (Wyatt Technologies) and GRAMS/AI 7.0 software (Thermo Fisher Scientific). The obtained chromatograms are summarized in the Supporting Information.

### 2.3 Dialdehyde Cellulose Synthesis

Disintegrated softwood kraft pulp (27.70 g, 1 equiv) was added to 1.65 L of a 0.15 M sodium periodate solution (52.04 g, 1.4 equiv). The mixture was stirred at 45 °C for 4.5 h in the dark to limit side reactions. The resulting DAC (DO = 39%) was filtered, washed thoroughly with deionized water, and stored never-dried at −20 °C. The DAC (DO = 8 %) from microcrystalline cellulose was prepared similarly by adding microcrystalline cellulose (20.00 g, 1 equiv) to 1.20 L of a 0.03 M sodium periodate solution (7.91 g, 0.3 equiv). The mixture was stirred for 24 h at room temperature.

### 2.4 Standard Procedure for the Reductive Amination of Dialdehyde Cellulose

The reductive amination protocol by Sirviö et al. was adapted to generate diamine celluloses from DAC.^55^ A 100 mL round-bottom flask was charged with the primary amine (4.1 equiv based on the aldehyde groups in DAC) and 50 mL of DI water. The mixture was adjusted to pH 4.5 with hydrochloric acid and sodium hydroxide. DAC (1 equiv) was added followed by 2-picoline borane (2 equiv based on the aldehyde groups in DAC). The mixture was stirred at 45 °C for 24 h. Methanol (150 mL) was added to the reaction mixture, followed by centrifugation and thorough washing of the solids with methanol. The resulting diamine celluloses were freeze-dried and analyzed by FTIR spectroscopy, DSC, TGA, EA, potentiometric titration, and liquid-state NMR spectroscopy. Each sample (60 mg) was stored in the never-dried state at −20 °C until being further characterized by GPC. The diamine celluloses from 8%-oxidized microcrystalline cellulose were freeze-dried and analyzed by FTIR spectroscopy, EA, potentiometric titration, GPC, and liquid-state NMR spectroscopy.

### 2.5 Determination of the Degree of Oxidation by Multivariate Calibration

The DO of DAC from softwood kraft pulp was determined from the FTIR spectrum combined with partial least squares regression according to our previous work.^69,70^ The published model was used to predict the DO from the recorded FTIR spectrum. The DO of DAC from microcrystalline cellulose was determined by potentiometric titration after treatment with hydroxylamine hydrochloride.

### 2.6 Determination of the Aldehyde Content by Potentiometric Titration

The remaining unreacted aldehyde groups in the diamine celluloses and the DO of DAC obtained from microcrystalline cellulose were determined by the quantitative reaction with hydroxylamine hydrochloride followed by titration to initial pH using sodium hydroxide solution.^72^ We slightly adapted the procedure previously reported for DACs.^69^ Hydroxylamine hydrochloride solution (0.25 M) was prepared by adding 3.3 g of hydroxylamine hydrochloride and 75.4 mg of sodium hydroxide to 200 mL of deionized water (pH after dissolution 4.5). The freeze-dried sample (18 to 22 mg) was added to 5.00 mL of the freshly prepared hydroxylamine hydrochloride solution. The mixtures were shaken for 44 h. Each mixture (5.00 mL) was diluted with 4 mL of deionized water and titrated against 0.01 M sodium hydroxide solution back to the initial pH. Each sample was measured in duplicate. The remaining aldehyde content CHO was calculated according to
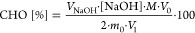
1with
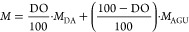
2where *M* is the molecular weight of the diamine cellulose, assuming the polymer to consist of aminated units and non-oxidized glucopyranose units only. DO is the degree of oxidation of the substrate (39 % or 8 %), *M*_DA_ is the molecular weight of the aminated glucopyranose unit, *M*_AGU_ is the molecular weight of the unoxidized glucopyranose unit (162.14 g mol^–1^),*V*_NaOH_ is the volume of NaOH consumed in the titration, [NaOH] is the concentration of the sodium hydroxide solution (0.01 M),*V*_0_ is the initial volume of the added hydroxylamine hydrochloride solution (5 mL), *V*_1_ is the volume of the titrated oxime solution (5 mL), and *m*_0_ is the mass of the DAC sample treated with hydroxylamine hydrochloride (18–22 mg). To calculate the DO of DAC from microcrystalline cellulose, the molecular weight *M* in eq 1 was assumed to equal the molecular weight of the unoxidized anhydroglucose unit (162.14 g mol^–1^). The derivation of eq 1 to calculate the aldehyde content CHO is described in the Supporting Information (p. S37).

### 2.7 Determination of the Degree of Substitution by Elemental Analysis

The DS of the diamine celluloses was calculated from the nitrogen content determined by EA according to
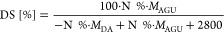
3where N % is the measured nitrogen content in percent, *M*_DA_ is the molecular weight of a diaminated unit, and *M*_AGU_ is the molecular weight of the non-oxidized glucopyranose unit (162.14 g mol^–1^). The derivation of eq 3 to calculate the DS is described in the Supporting Information (p. S38).

## 3 Results and Discussion

### 3.1 Synthesis and Characterization of the Diamine Celluloses Derived from Microcrystalline Cellulose via Dialdehyde Cellulose

**Scheme 3:**
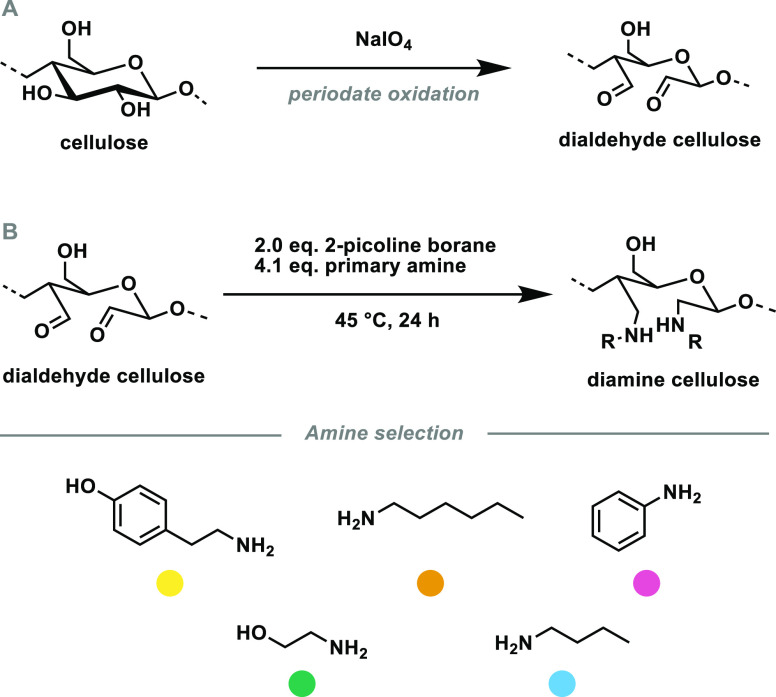
Synthesis of Diamine Celluloses from Cellulose in Two Steps: (A) Periodate Oxidation and (B) Reductive Amination, Using Five Different Primary Amines

For the preparation of the diamine celluloses via DAC, we used 2-picoline borane according to an adapted protocol by Sirviö et al.^55^ Five different primary amines were brought to reaction with partially periodate-oxidized cellulose (Scheme 3). Butylamine and hexylamine represent two aliphatic amines, ethanolamine and tyramine represent aliphatic amino alcohols, and aniline represents an aromatic amine. Tyramine was included as a naturally occurring amine which is obtained by decarboxylation of the amino acid tyrosine. Besides the anticipated influence on the material properties, the different side chains also affect solubility, nucleophilicity, basicity, and the steric demand of the educts.

Consequently, different reaction outcomes and conversion rates are expected. To establish proper structure/property relationships, we focused on chemical characterization and analysis of potential side reactions in the first part of this study, performing solution-state NMR experiments and cross-validating the results with FTIR, EA, and determination of the remaining aldehyde content by potentiometric titration. The relatively high DO (39%) of the oxidized softwood kraft pulp and its more complex chemical structure (due to the presence of hemicelluloses; compare Figure S5 and Tables S1 and S2) led to significant peak superposition, complicating assignment of the signals (compare Figure S35). Thus, at first, we resorted to the well-studied and less heavily oxidized (8%) microcrystalline cellulose Avicel PH-101 as an easier-to-analyze model for the NMR experiments.^73^ Spectra of selected samples of softwood kraft pulp derivatives recorded for comparison confirmed the similarity of the introduced moieties (Figures S34–S43).

The binary NMR solvent [P_4444_][OAc]/DMSO-*d*_6_ (w/w = 1:4) had been previously used to investigate high-DO DACs obtained from CNCs.^62^ Thereby, complete degradation to low-molecular-weight compounds owing to beta-alkoxy elimination in the inherently alkaline acetate-based electrolyte was observed,^58^ and no aldehyde or hemiacetal functionalities could be assigned. Similarly, the DAC (DO of 39%) derived from softwood kraft pulp completely disintegrated and depolymerized, as evidenced by the absence of peaks in the diffusion-edited ^1^H experiment (Figure S37). In contrast, the less modified DAC (DO of 8%) from microcrystalline cellulose showed an increase in the end group peak intensities, suggesting only a partial degradation. Both quantitative ^1^H spectra showed a prominent peak around 8.60 ppm, which was previously assigned to formiate (HCOO^–^) as one of the major end products of alkaline degradation of DAC.^63,74^ We found this formiate resonance to be a reasonable marker for the presence of residual DAC moieties in NMR measurements of its derivatives.

**Figure 1:**
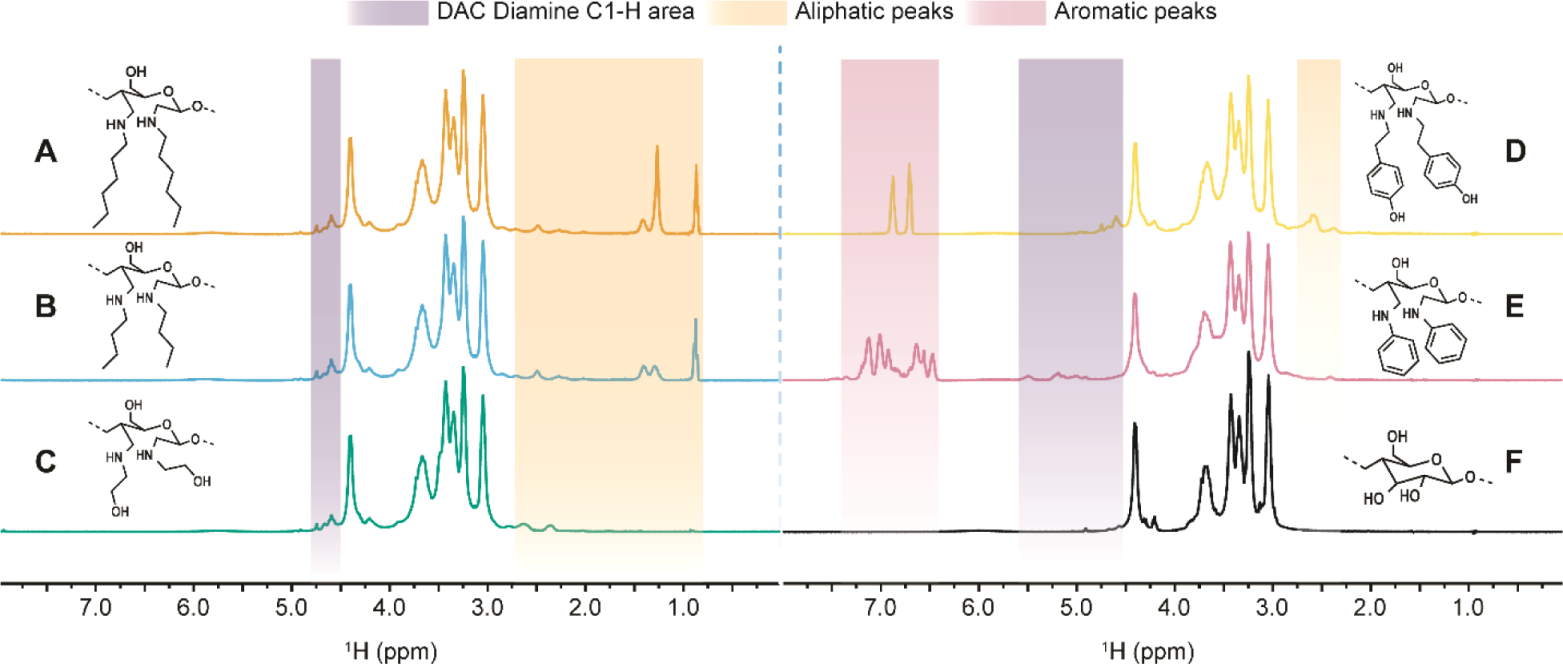
Comparison of diffusion-edited ^1^H NMR spectra ([P_4444_][OAc]/DMSO-*d*_6_ (w/w = 1:4); 400 MHz; 65 °C) of diamine celluloses prepared from partially oxidized microcrystalline cellulose (5 wt %; DO = 8%): (A) dihexylamine, (B) dibutylamine, (C) diethanolamine, (D) dityramine, and (E) dianiline; (F) spectrum of unmodified microcrystalline cellulose for comparison. Besides the resonances of the cellulose backbone between 3 and 4.4 ppm, three distinct spectral regions for the characterization of the products are discernible. The diamine cellulose C1–H area from 4.5 to 5.5 ppm shows characteristic peaks at 4.74 and 4.59 ppm for all derivatives of aliphatic amines and at 5.49 and 5.18 ppm for the aromatic dianiline cellulose (E). In accordance with the introduced residues, the diamine celluloses show resonances in the aliphatic spectral area below 3 ppm (A–D) or the aromatic region around 6 to 8 ppm (D,E). No peaks of products from possible side reactions were visible. Note: the diffusion-edited ^1^H experiment blinds out resonances of low-molecular-weight compounds present in the NMR sample (H_2_O, [P_4444_][OAc], DMSO-*d*_6_, and impurities from degradation).^63^ Thus, the visible peaks originate from polymeric molecules, proving the covalent modification of the cellulose backbone.

The prepared diamine celluloses showed no signs of significant degradation during the rather harsh dissolution and measuring conditions, thus suggesting a stabilizing effect of the modifications toward alkaline conditions. Fast and reliable evidence of the formation of the anticipated structures was obtained from the diffusion-edited ^1^H spectra (Figure 1). This type of NMR experiment removes the resonances of all low-molecular-weight constituents in the sample, leaving only signals of the polymeric constituents behind.^63^ All five derivatives showed additional resonances from the amine moieties in the expected spectral regions. Remarkably, even the aliphatic signals in the dibutylamine and dihexylamine celluloses were well resolved, despite the superposition with the intensive solvent resonances. The further assignment of the spin systems of the modified glucopyranose units was complicated, even by multiple bond correlated 2D NMR spectroscopy. Apparently, supramolecular phenomena introduced by the scission of the backbone ring structure led to peak splitting and broadening. Thus, only the characteristic peak areas for different moieties can be given. For example, the C1–H moiety of all aliphatic diamine derivatives showed two characteristic peaks at 4.74 and 4.59 ppm.

The screening of the NMR spectra for potential side reactions (Scheme 2) produced no resonances from either imine or enamine structures, proving a complete conversion of the intermediates. The quantitative reduction is furthermore supported by the absence of bands from imine or enamine functional groups in the FTIR spectra (compare Figures S44 to S55). A comparison of the diamine spectra with a dialcohol cellulose prepared by reduction with sodium borohydride showed that the 2-picoline borane reduction was selective for imine moieties and that 2-picoline borane did not reduce the carbonyl (aldehyde) groups under the given conditions. Characteristic resonances of the dialcohol acetal C1 moiety (HSQC shows two peaks at 4.95/103.9 and 4.76/103.6 ppm; Figure S18) were absent in all diamine derivatives. We cannot definitely rule out the formation of cyclic seven-membered structures from the NMR data. However, the resonances in the aromatic region of the HSQC spectrum of the dianiline cellulose (Figure S33) strongly indicate derivatization at both C2 and C3. The quantitative ^1^H spectra of all prepared diamine celluloses showed a formiate degradation peak around 8.60 ppm, indicating some degradation into low-molecular components.

To verify the assumption of incomplete conversion of the carbonyl groups, we determined the DS from the nitrogen content by EA and the remaining aldehyde moieties (CHO) by potentiometric titration (after treatment with hydroxylamine hydrochloride; Table 1).

**Table 1:**
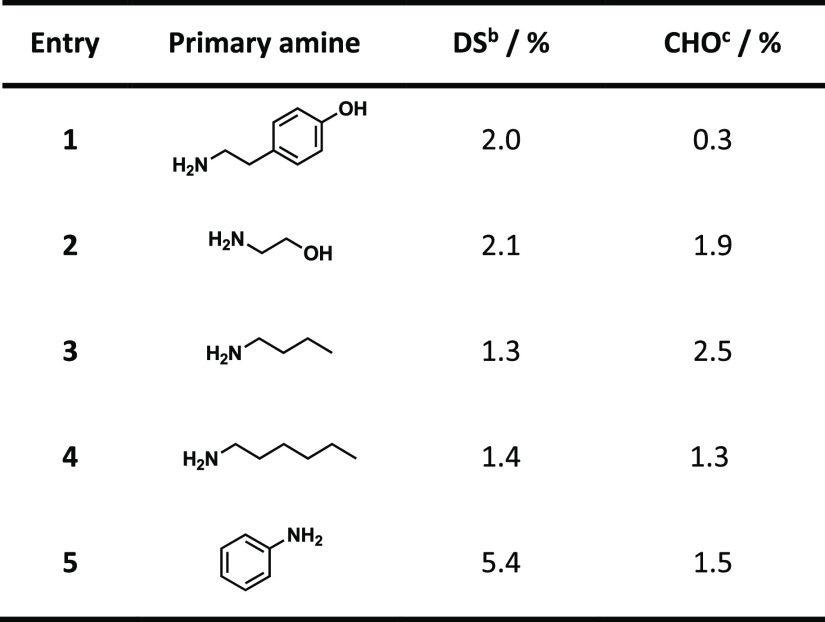
Diamine Celluloses from 8%-Oxidized Microcrystalline Cellulose and Five Different Primary Amines^a^

a Reaction was conducted in deionized water at 45 °C for 24 h with 2.0 equiv of 2-picoline borane.

b DS calculated from the nitrogen content determined by EA.

c Aldehyde content determined from potentiometric titration after oximation with hydroxylamine hydrochloride.

The calculated DS values supported the observations from the NMR experiments, indicating incomplete conversion of the aldehyde moieties in DAC to the corresponding amines. In the case of all five diamine celluloses, the isolated materials contained non-reacted aldehyde groups. However, the reductive amination using aniline showed a significantly better conversion with a DS value of 5.4 %, which is about two-thirds of the aldehyde groups in the DAC (DO = 8 %). The diamine celluloses obtained from reacting aliphatic amines with DAC (entries 1–4 in Table 1) contained only 1 to 2 % amines, a conversion of less than 25 % of the carbonyl groups in the DAC substrate. Only when using aniline (entry 5), the calculated remaining aldehyde groups and the incorporated aniline groups add up to the DO of the DAC used (DO of 8 %), and only a minute amount of the DAC moieties were lost via beta-elimination to low-molecular-weight and water-soluble fractions. In the case of the reductive amination with aliphatic amines (entries 1–4), it seems that most of the carbonyl groups are lost in low-molecular-weight fractions, while a smaller fraction remains non-reacted in the polymer backbone. The different conversion rates are also supported by the relative peak intensities of the introduced moieties compared to the cellulose CH-1 proton resonance in the qualitative diffusion-edited ^1^H spectra. A more exact evaluation from the quantitative ^1^H experiment was prevented by significant peak splitting and superposition. The difference in the conversion rates can be explained by two effects: stabilization of the imine intermediate and basicity of the primary amine. For the reductive amination with aniline, the imine intermediate is stabilized by resonance with the aromatic π-system. In addition, aniline is a much weaker base than the aliphatic amines, owing to mesomeric delocalization effects of the nitrogen lone pair. This lower basicity of aniline limits beta-elimination processes. Both effects cooperate and favor the reductive amination with aniline, increasing the carbonyl groups’ conversion to the corresponding dianiline cellulose.

### 3.2 Preparation of Thermoplastics from Softwood Kraft Pulp

In the second part of this study, we focused on the preparation of thermoplastic materials by applying the investigated periodate oxidation/reductive amination pathway to softwood kraft pulp. Working with softwood kraft pulp instead of microcrystalline cellulose has two major advantages when it comes to material development: (1) the high molecular weight of the pulp also leads to high-molecular-weight derivatives and (2) the production of pulp necessitates less purification and processing steps, which makes the final materials less expensive. Both factors are essential when preparing thermoplastics (e.g., for packaging applications) which are meant to compete with fossil-based alternatives.

We aimed for a sufficiently high DO (39 %) of glucopyranose units in the softwood kraft pulp to generate enough “soft” segments in the polymer backbone to observe thermoplastic behavior in the derived diamine celluloses, while maintaining as many unmodified units as possible. On the one hand, the rigidity of the unmodified glucopyranose units increases the *T*_g_, while, on the other hand, only partial modification of the cellulosic substrate saves resources (i.e., time and chemicals). After periodate oxidation of the softwood kraft pulp, we applied the previously optimized reduction conditions to prepare five diamine celluloses from the 39%-oxidized softwood kraft pulp. Since the chemical composition of the pulp is more complex—due to the content of hemicellulose and modified hemicellulose which leads to peak overlap in the solution-state NMR spectra—we limited the characterization to the remaining aldehyde content (CHO) and the DS by potentiometric titration and EA, respectively (Table 2). The conversion rates of the pulp-derived diamine celluloses were similar to those in the previous set of experiments with microcrystalline cellulose. All five isolated diamine celluloses contained residual aldehyde groups. In the aliphatic amines (Table 2, entries 1–4), only a fraction of the carbonyl groups were aminated (with DS values between 8 and 13 %). However, the reductive amination with aniline led to about 84 % conversion of the introduced carbonyl groups (DS value of 32.6 %), while only a few aldehyde groups remained unreacted. Observed side reactions were, as mentioned above, beta-alkoxy elimination and incomplete conversion. The FTIR data (Figures S50–S55) and the NMR spectra (examples of dianiline and diethanolamine cellulose, Figures S38–S43) indicated no other side reactions. The derivatization of microcrystalline cellulose thus proved to be a suitable model system to investigate and optimize the reaction pathway beforehand. The conversions of both cellulosic substrates were in good agreement.

**Table 2:**
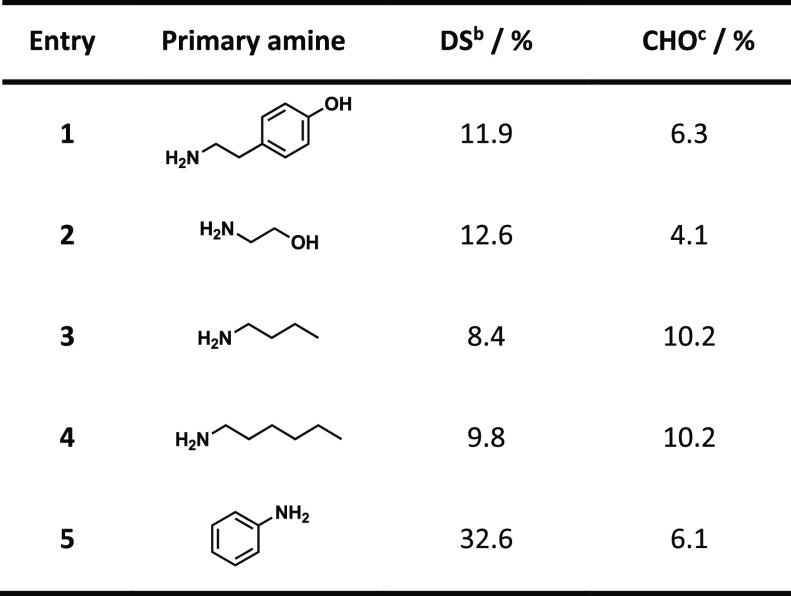
Diamine Celluloses from 39%-Oxidized Softwood Kraft Pulp and Five Different Primary Amines^a^

a Reaction conducted in deionized water at 45 °C for 24 h with 2.0 equiv of 2-picoline borane.

b DS calculated from the nitrogen content determined by EA.

c Aldehyde content determined from potentiometric titration after oximation with hydroxylamine hydrochloride.

### 3.3 Effect of Reductive Amination on Molecular Weight Distribution

**Figure 2:**
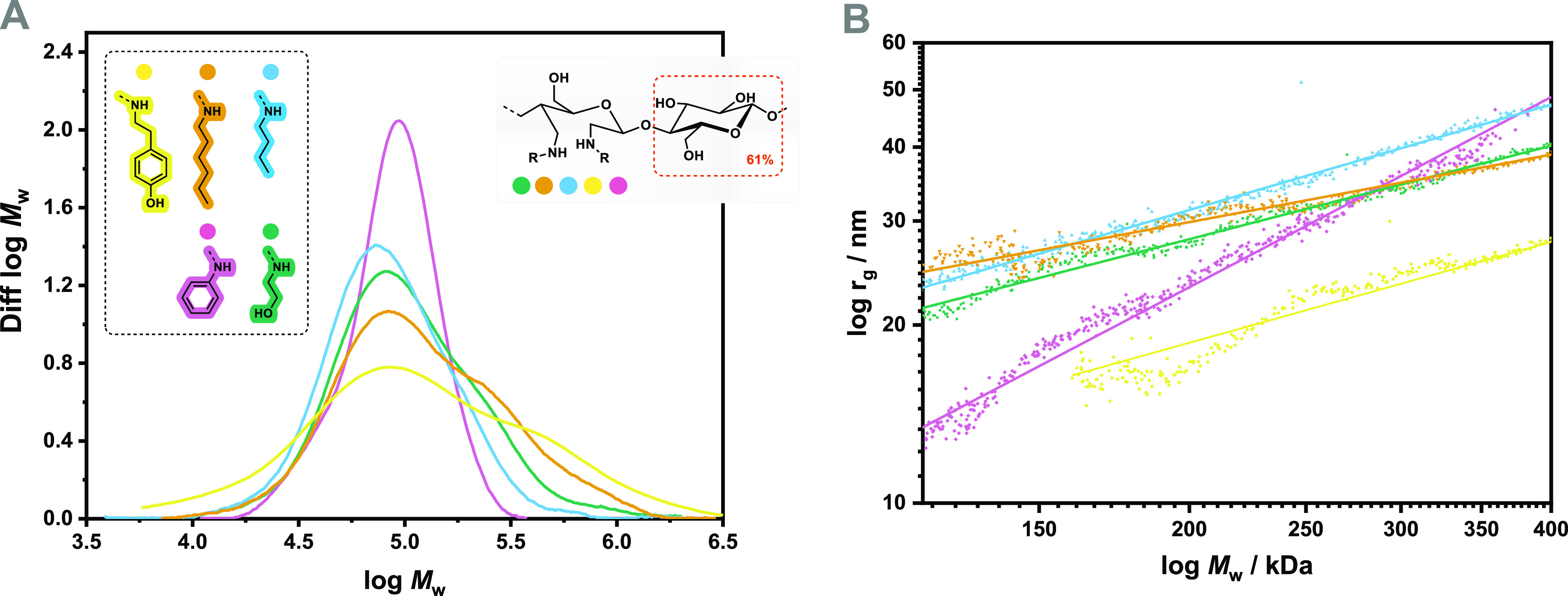
Molecular weight distributions (A) and conformation plots (B) of diamine celluloses derived from periodate oxidation of softwood kraft pulp followed by reductive amination with five different amines using the 2-picoline borane reductant.

The isolated diamine celluloses derived from 39%-oxidized softwood kraft pulp were analyzed by GPC in an *N*,*N*-dimethylacetamide/lithium chloride eluant. All five isolated diamine celluloses were high-molecular-weight polymers with *M*_n_ values between 60 and 82 kDa (Table 3). However, the molecular weight distributions of all diamine celluloses had decreased compared to the untreated starting pulp (Figure S2) and lost their usual bimodal shape (Figure 2A). The depolymerization of the polymer chains can be explained by degradation during periodate oxidation and beta-elimination during reductive amination. Since only a fraction of the introduced carbonyl groups was aminated in the second step, the molecular weight distributions (except for dianiline cellulose) showed a high-molecular-weight fraction due to cross-linking via hemiacetal formation involving the remaining carbonyl moieties. Dityramine cellulose (Table 3, entry 1) showed the broadest molecular weight distribution (*D̵* of 4.11) of all five diamine celluloses. It is reasonable to assume that the hydroxy groups of the bulky tyramine substituent participate in hemiacetal formation with the non-reacted aldehyde groups, as an additional cross-linking option.

**Table 3:**
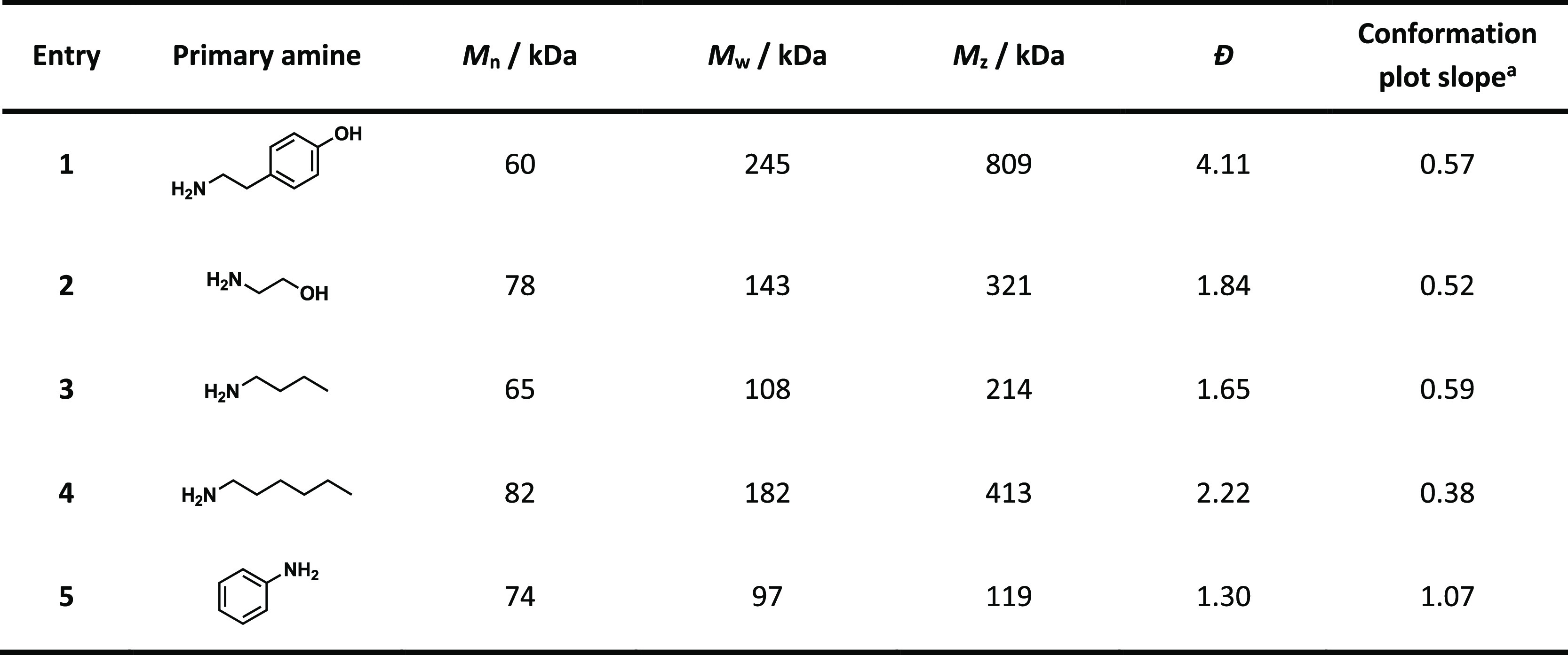
GPC–MALLS Statistical Moments for the Diamine Celluloses Obtained from Softwood Kraft Pulp

a Slope of the linear regression of the multiangle light-scattering data in the conformation plot.

Although the isolated dianiline cellulose also contains some unreacted aldehyde groups, the molecular weight distribution is almost a neat Gaussian distribution (Figure 2A) with a dispersity *D̵* of 1.30 (Table 3). One reason for this observation is that the remaining aldehyde groups in dianiline cellulose form intramolecular hemiacetal linkages instead of interchain cross-links. This theory is supported by the unusual change in the slope of the conformational plot (Figure 2B and Table 3), that is, radius of gyration versus molecular weight. The linear regression of the light scattering data has a slope close to 1, indicating a rod-like dissolution structure.^75^ We speculate that interactions of the introduced aromatic rings force the polymer chains into a rod-like structure due to quadrupolar and π–π stacking interactions. This phenomenon has been amply described for other classes of macromolecules containing aromatic groups.^76^

The slopes of the conformation plots of the other diamine celluloses, except the product from hexylamine, are about 0.5 to 0.6. A slope between 0.5 and 0.6 is typical for random coils in solution.^75^ Interestingly, the slope of the conformation plot of dihexyl cellulose (Table 3, entry 4) is significantly lower, proving a much more compact structure. A slope of approx. 1/3 is typical for spherical polymers in solution.^75^ This observation could be explained by hydrophobic interactions of the relatively long alkyl residues. Nevertheless, it is surprising that this change in the tertiary structure was not observed for dibutyl cellulose, in which the alkyl chains are only a little shorter.

### 3.4 Thermal Properties

The thermal properties of a polymer define its range of applications and its processability. In this work, we used TGA and DSC to investigate the polymers’ thermal degradation and to determine their *T*_g_, respectively. The generated diamine celluloses were relatively stable and degraded above 207 °C (Figure 2A and Table 3). The *T*_95_ values—defining the temperature of 5 % mass loss—of all diamine celluloses exceed the *T*_95_ value of the initial DAC (200 °C). Therefore, the reductive amination apparently did not impair, but even slightly improves, the thermal stability of the polymer. The effect is the most pronounced for dianiline cellulose (Table 4, entry 5), with a *T*_95_ value of 275 °C. This was expected since the dianiline cellulose contains three times more amines than the other diamine celluloses. In the case of the *N*-alkyl-substituted derivatives, the thermal stability increases slightly with the length of the side chain.

**Table 4:**
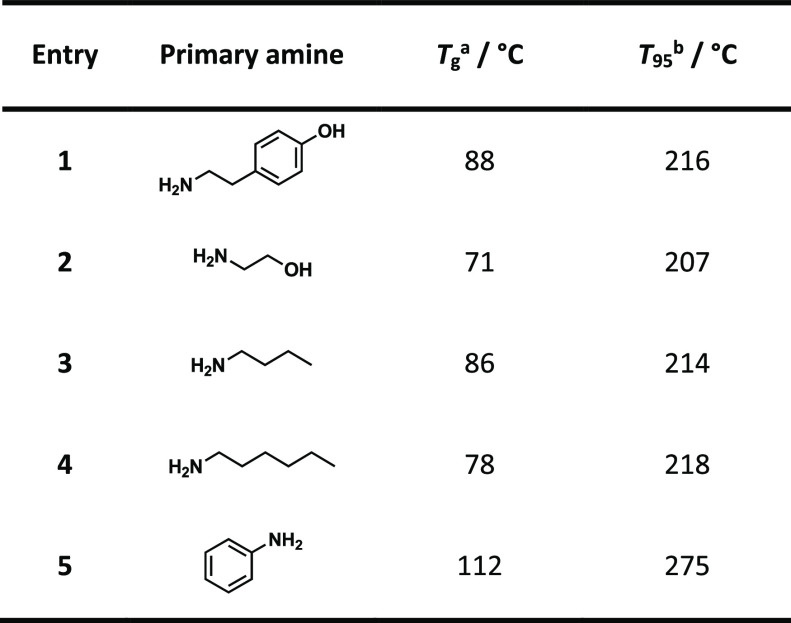
Thermal Data of the Diamine Celluloses Derived from Softwood Kraft Pulp

a Determined by DSC.

b Temperature at 5% mass loss by TGA.

**Figure 3:**
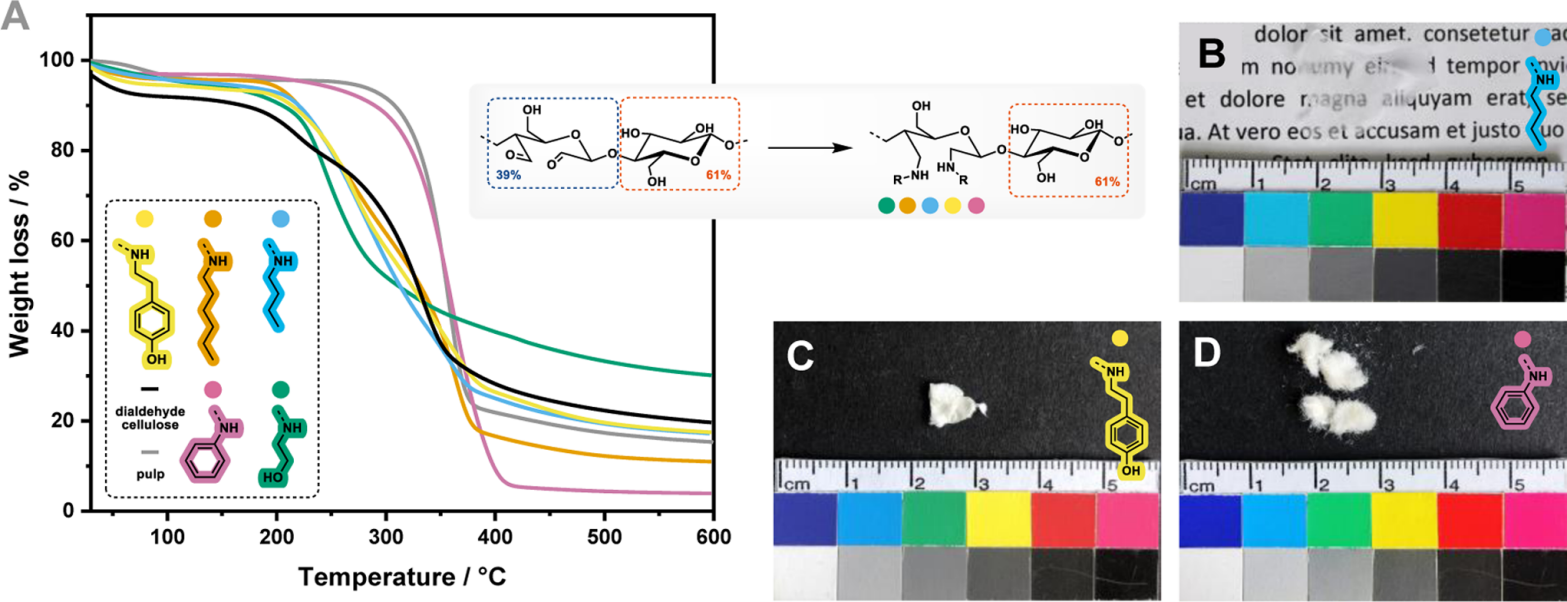
TGA data (A) and photographic images of the softwood kraft pulp-derived diamine celluloses from butylamine (B), tyramine (C), and aniline (D) after freeze-drying.

The diamine celluloses also had different physical appearances after freeze-drying (Figure 3B–D). Although the *N*-alkyl- and ethanolamine-substituted diamine celluloses formed transparent films, the films from derivatives containing aromatic groups remained opaque. For many applications, transparent polymers are preferred, especially in the packaging industry.

**Figure 4:**
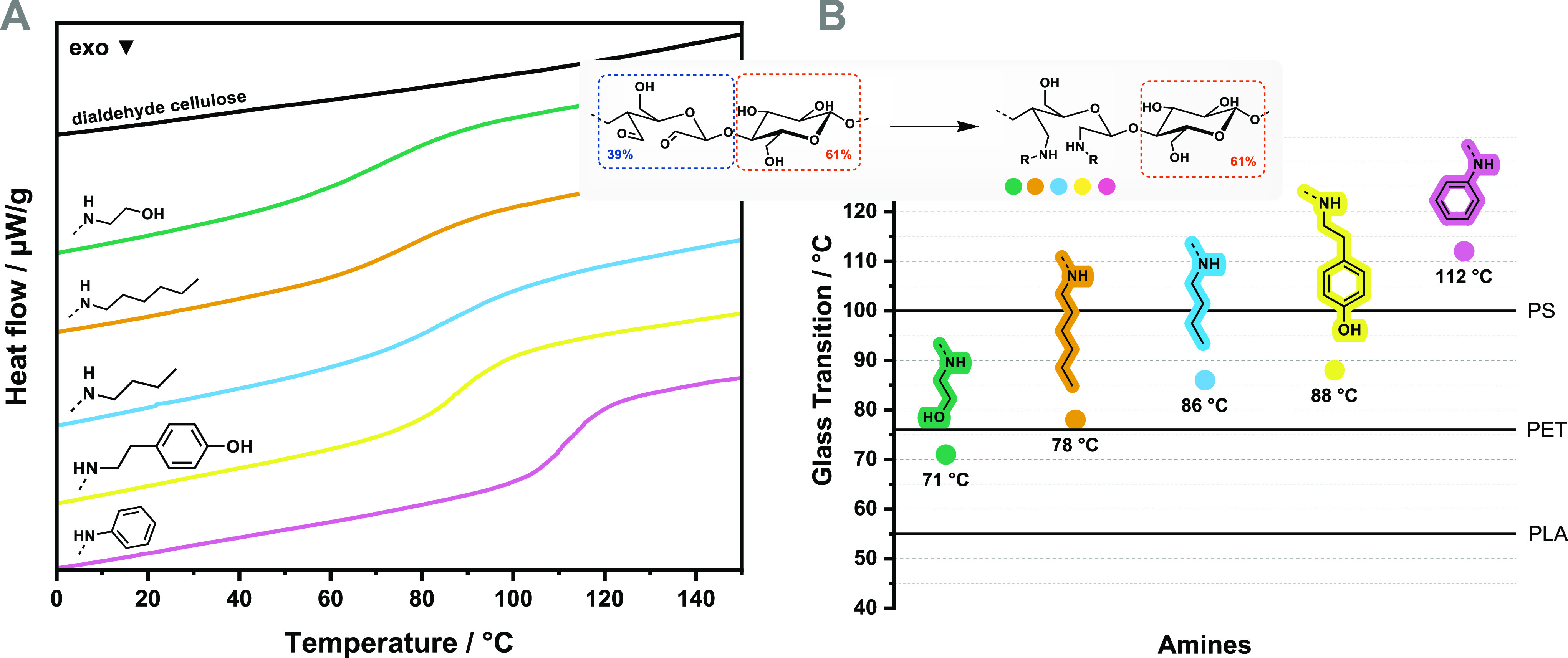
DSC thermograms (A) and obtained glass transition temperatures (B) of the diamine celluloses from softwood kraft pulp compared to those of commodity plastics polystyrene (PS), polyethylene terephthalate (PET), and polylactic acid (PLA).

As expected, the substituents highly influenced the *T*_g_. The diamine celluloses exhibited *T*_g_ values from 71 to 112 °C (Figure 4 and Table 4). However, the influence of the *N*-phenyl group on *T*_g_ cannot be compared directly with the other diamine celluloses since the DS differs significantly. Nevertheless, for other polymers, it is known that the high conformational restriction and strong polar associations (quadrupolar and π–π stacking interactions) lead to high-*T*_g_ thermoplastics.^4^ Dianiline cellulose showed the highest *T*_g_ of the entire series (112 °C).

Despite their low DS, also the other four diamine celluloses showed thermoplastic behavior with attractive *T*_g_ values. Since the DS of the aliphatic diamine celluloses is in a similar range, the influence of the substituent on *T*_g_ can be explained by polymer/structure relationships. Two opposing effects mainly influence *T*_g_: (A) side chains with conformational restrictions and polar associations increase *T*_g_ and (B) additional free volume decreases *T*_g_.^4^ For example, when replacing the *N*-butyl substituent with the *N*-hexyl group, additional free volume is introduced and *T*_g_ decreases moderately (Table 4, entry 4 and 5). The tyramine group (Table 4, entry 1) adds free volume, but the hydroxyphenyl substituent also increases interchain attractions. The *T*_g_ is close to the *T*_g_ of the *N*-butyl substituted polymer. The introduction of ethanolamine gave the lowest *T*_g_ of the entire series (71 °C), although the *N*-ethanol substituent adds less free volume than the *N*-butyl or *N*-hexyl substituent. This finding can be explained by the decreased amount of non-reacted aldehyde groups along the polymer backbone. Decreased cross-linking due to hemiacetal formation weakens the interchain binding of the polymer chains and leads to a lower *T*_g_ value.

All diamine celluloses were stable at high temperatures with *T*_95_ values above 200 °C and exhibited *T*_g_ values in the range of commercially available thermoplastics, such as PET (Figure 3B). The *T*_g_ of dianiline cellulose (112 °C) exceeded that of polystyrene (100 °C). No melting temperature was detected by DSC. Thus, the presumed processing temperature of all five diamine celluloses is above their *T*_g_ values, which was practically confirmed by hot-pressing the materials using a commercial hair straightener. The thermal processing of these diamine celluloses is straightforward with a broad processing window, as the *T*_g_ values are well below their degradation temperatures (*T*_95_).

## 4 Conclusions and Outlook

A previously reported procedure for reductive amination of DAC with 2-picoline borane was adapted as a pathway to synthesize diamine celluloses, as thermoplastics from softwood kraft pulp. Conducting the reaction first on a microcrystalline cellulose offered a means for optimization and training of the analytical approaches. The combination of solution-state NMR spectroscopy, EA, and potentiometric titration allowed comprehensive analytical characterization of the diamine cellulose products. The reductive amination was incomplete, leaving residual carbonyl groups in the polymer backbone behind. Even using mild and slightly acidic conditions, beta-elimination could not be avoided. Both conversion and beta-elimination were found to be strongly dependent on the introduced primary amine. The stabilizing effect of aniline on the imine intermediate and its lower basicity led to significantly higher conversion and less beta-elimination in this case.

Using five different primary amines and a 39%-oxidized softwood kraft pulp, we generated a series of thermoplastics and analyzed the effect of the substituent on the thermal properties. Despite the residual hemiacetal cross-links and the low DS, all examined diamine celluloses exhibited high *T*_g_ values (71 to 112 °C), which were in the range of commercially available thermoplastics (e.g., PET and PS). Moreover, the diamine celluloses were stable at high temperatures with a *T*_95_ value between 207 and 275 °C. The aniline derivative showed the highest *T*_g_ and *T*_95_ values of all investigated samples. The generated diamine celluloses showed high molecular weights with *M*_n_ values greater than 60 kDa. However, the molecular weight distributions were shifted to significantly lower values compared to the starting softwood kraft pulp. Four of five samples showed a high-molecular-weight fraction, as expected for residual hemiacetal cross-links being present. Only the dianiline cellulose exhibited a narrow molar mass distribution (*D̵* of 1.30), in addition to supramolecular effects of π–π interactions of the aromatic substituents, leading to a rod-like structure in solution.

With this work, we demonstrated the influence of the introduced amine on the thermal properties of DAC-derived diamine celluloses and showed that even a relatively low DS can lead to thermoplastic derivatives. However, although often disregarded in the literature, the transformation of DAC to the corresponding amines was not quantitative and was accompanied by degradative side reactions. These phenomena likewise influence the thermal properties. Dianiline cellulose showed the highest conversion, the best thermal properties, and a peculiar, symmetric molecular weight distribution.

Follow-up chemistry should exploit the stabilizing effect of some substituents on the imine intermediate which apparently increases the conversion. Since working under slightly acidic conditions does not suppress beta-elimination reactions, amines with high basicity should be avoided to minimize these side reactions. In addition, optimized reaction conditions are necessary to improve the conversion rate to make more use of the introduced aldehyde groups and to decrease interchain cross-linking. While we concentrated, in this work, on introducing simple primary amines to demonstrate the principle, future work toward replacement of commodity plastics will focus on non-toxic and renewable amines. Similarly, further work is needed in material development and investigation of material properties, in addition to the thermal properties already investigated. Although 2-picoline borane had been classified as non-toxic for decades, latest data might indicate (eco)toxicity. Therefore, careful purification of the generated material and good recycling strategies are crucial during material development and optimization. These issues will be addressed in an upcoming account.

## Supporting Information Available

Additional experiment details, including characterization of the cellulosic starting materials, cellulose solution state NMR data, FTIR spectra, GPC/MALLS–RI data, and derivation of the used formulas (PDF)

## References

[ref1] MacLeod M.; Arp H. P. H.; Tekman M. B.; Jahnke A. The Global Threat from Plastic Pollution. Science 2021, 373, 61–65. 10.1126/science.abg5433. 34210878

[ref2] Shafiee S.; Topal E. When Will Fossil Fuel Reserves Be Diminished?. Energy Policy 2009, 37, 181–189. 10.1016/j.enpol.2008.08.016.

[ref3] Avérous L.; Pollet E. Biodegradable Polymers. Environmental Silicate Nano-Biocomposites; Avérous L., Pollet E., Eds.; Springer London: London, 2012; pp 13–39.

[ref4] Nguyen H. T. H.; Qi P.; Rostagno M.; Feteha A.; Miller S. A. The Quest for High Glass Transition Temperature Bioplastics. J. Mater. Chem. A 2018, 6, 9298–9331. 10.1039/C8TA00377G.

[ref5] Mazeau K.; Heux L. Molecular Dynamics Simulations of Bulk Native Crystalline and Amorphous Structures of Cellulose. J. Phys. Chem. B 2003, 107, 2394–2403. 10.1021/jp0219395.

[ref6] Glasser W. G.; Atalla R. H.; Blackwell J.; Malcolm Brown R.; Burchard W.; French A. D.; Klemm D. O.; Nishiyama Y. About the Structure of Cellulose: Debating the Lindman Hypothesis. Cellulose 2012, 19, 589–598. 10.1007/s10570-012-9691-7.

[ref7] Szcześniak L.; Rachocki A.; Tritt-Goc J. Glass Transition Temperature and Thermal Decomposition of Cellulose Powder. Cellulose 2008, 15, 445–451. 10.1007/s10570-007-9192-2.

[ref8] Li C.; Wu J.; Shi H.; Xia Z.; Sahoo J. K.; Yeo J.; Kaplan D. L. Fiber-Based Biopolymer Processing as a Route toward Sustainability. Adv. Mater. 2022, 34, 2105196 10.1002/adma.202105196. PMC8741650 34647374

[ref9] Weber C. O.; Cross C. F. Process of Making Cellulose Esters. U.S. Patent 632,605 A, 1898.

[ref10] Cross C. F.; Bevan E. J. Manufacture of Cellulose Acetate. U.S. Patent 580,826 A, 1894.

[ref11] Luan Y.; Wu J.; Zhan M.; Zhang J.; Zhang J.; He J. One Pot Homogeneous Synthesis of Thermoplastic Cellulose Acetate-Graft-Poly(l-Lactide) Copolymers from Unmodified Cellulose. Cellulose 2013, 20, 327–337. 10.1007/s10570-012-9818-x.

[ref12] Hou D.-F.; Li M.-L.; Yan C.; Zhou L.; Liu Z.-Y.; Yang W.; Yang M.-B. Mechanochemical Preparation of Thermoplastic Cellulose Oleate by Ball Milling. Green Chem. 2021, 23, 2069–2078. 10.1039/D0GC03853A.

[ref13] Tanaka S.; Iwata T.; Iji M. Long/Short Chain Mixed Cellulose Esters: Effects of Long Acyl Chain Structures on Mechanical and Thermal Properties. ACS Sustainable Chem. Eng. 2017, 5, 1485–1493. 10.1021/acssuschemeng.6b02066.

[ref14] Boulven M.; Quintard G.; Cottaz A.; Joly C.; Charlot A.; Fleury E. Homogeneous Acylation of Cellulose Diacetate: Towards Bioplastics with Tuneable Thermal and Water Transport Properties. Carbohydr. Polym. 2019, 206, 674–684. 10.1016/j.carbpol.2018.11.030. 30553372

[ref15] Jebrane M.; Terziev N.; Heinmaa I. Biobased and Sustainable Alternative Route to Long-Chain Cellulose Esters. Biomacromolecules 2017, 18, 498–504. 10.1021/acs.biomac.6b01584. 28084073

[ref16] Chen Z.; Zhang J.; Xiao P.; Tian W.; Zhang J. Novel Thermoplastic Cellulose Esters Containing Bulky Moieties and Soft Segments. ACS Sustainable Chem. Eng. 2018, 6, 4931–4939. 10.1021/acssuschemeng.7b04466.

[ref17] Kasai W.; Morooka T.; Ek M. Mechanical Properties of Films Made from Dialcohol Cellulose Prepared by Homogeneous Periodate Oxidation. Cellulose 2014, 21, 769–776. 10.1007/s10570-013-0153-7.

[ref18] Larsson P. A.; Berglund L. A.; Wågberg L. Ductile All-Cellulose Nanocomposite Films Fabricated from Core–Shell Structured Cellulose Nanofibrils. Biomacromolecules 2014, 15, 2218–2223. 10.1021/bm500360c. 24773125

[ref19] Larsson P. A.; Berglund L. A.; Wågberg L. Highly Ductile Fibres and Sheets by Core-Shell Structuring of the Cellulose Nanofibrils. Cellulose 2014, 21, 323–333. 10.1007/s10570-013-0099-9.

[ref20] López Durán V.; Larsson P. A.; Wågberg L. On the Relationship between Fibre Composition and Material Properties Following Periodate Oxidation and Borohydride Reduction of Lignocellulosic Fibres. Cellulose 2016, 23, 3495–3510. 10.1007/s10570-016-1061-4.

[ref21] Lei B.; Feng Y. Sustainable Thermoplastic Bio-Based Materials from Sisal Fibers. J. Cleaner Prod. 2020, 265, 121631 10.1016/j.jclepro.2020.121631.

[ref22] Esen E.; Meier M. A. R. Sustainable Functionalization of 2,3-Dialdehyde Cellulose via the Passerini Three-Component Reaction. ACS Sustainable Chem. Eng. 2020, 8, 15755–15760. 10.1021/acssuschemeng.0c06153.

[ref23] Park H.-M.; Misra M.; Drzal L. T.; Mohanty A. K. “Green” Nanocomposites from Cellulose Acetate Bioplastic and Clay: Effect of Eco-Friendly Triethyl Citrate Plasticizer. Biomacromolecules 2004, 5, 2281–2288. 10.1021/bm049690f. 15530043

[ref24] Gonçalves S. M.; dos Santos D. C.; Motta J. F. G.; dos Santos R. R.; Chávez D. W. H.; de Melo N. R. Structure and Functional Properties of Cellulose Acetate Films Incorporated with Glycerol. Carbohydr. Polym. 2019, 209, 190–197. 10.1016/j.carbpol.2019.01.031. 30732798

[ref25] Edgar K. J.; Buchanan C. M.; Debenham J. S.; Rundquist P. A.; Seiler B. D.; Shelton M. C.; Tindall D. Advances in Cellulose Ester Performance and Application. Prog. Polym. Sci. 2001, 26, 1605–1688. 10.1016/S0079-6700(01)00027-2.

[ref26] Cortina-Puig M.; Hurtado-Fernandez E.; Lacorte S. Plasticizers in Drinking Water and Beverages. Curr. Anal. Chem. 2018, 14, 344–357. 10.2174/1573411013666170922145949.

[ref27] Ma Y.; Liao S.; Li Q.; Guan Q.; Jia P.; Zhou Y. Physical and Chemical Modifications of Poly (Vinyl Chloride) Materials to Prevent Plasticizer Migration-Still on the Run. React. Funct. Polym. 2020, 147, 104458 10.1016/j.reactfunctpolym.2019.104458.

[ref28] Soyama M.; Iji M. Improving Mechanical Properties of Cardanol-Bonded Cellulose Diacetate Composites by Adding Polyester Resins and Glass Fiber. Polym. J. 2017, 49, 503–509. 10.1038/pj.2017.10.

[ref29] Jackson E. L.; Hudson C. S. Application of the Cleavage Type of Oxidation by Periodic Acid to Starch and Cellulose1. J. Am. Chem. Soc. 1937, 59, 2049–2050. 10.1021/ja01289a077.

[ref30] Nypelö T.; Berke B.; Spirk S.; Sirviö J. A. Review: Periodate Oxidation of Wood Polysaccharides—Modulation of Hierarchies. Carbohydr. Polym. 2021, 252, 117105 10.1016/j.carbpol.2020.117105. 33183584

[ref31] Sirvio J.; Hyvakko U.; Liimatainen H.; Niinimaki J.; Hormi O. Periodate Oxidation of Cellulose at Elevated Temperatures Using Metal Salts as Cellulose Activators. Carbohydr. Polym. 2011, 83, 1293–1297. 10.1016/j.carbpol.2010.09.036.

[ref32] Alam M.; Antal M.; Tejado A.; van de Ven T. G. M. Salt-Induced Acceleration of Chemical Reactions in Cellulose Nanopores. Cellulose 2012, 19, 517–522. 10.1007/s10570-011-9649-1.

[ref33] Sirviö J.; Liimatainen H.; Niinimäki J.; Hormi O. Dialdehyde Cellulose Microfibers Generated from Wood Pulp by Milling-Induced Periodate Oxidation. Carbohydr. Polym. 2011, 86, 260–265. 10.1016/j.carbpol.2011.04.054.

[ref34] Aimin T.; Hongwei Z.; Gang C.; Guohui X.; Wenzhi L. Influence of Ultrasound Treatment on Accessibility and Regioselective Oxidation Reactivity of Cellulose. Ultrason. Sonochem. 2005, 12, 467–472. 10.1016/j.ultsonch.2004.07.003. 15848109

[ref35] National Center for Biotechnology. PubChem Compound Summary for CID 23675764, Sodium iodate. https://pubchem.ncbi.nlm.nih.gov/compound/Sodium-iodate (accessed May 11, 2022).

[ref36] National Center for Biotechnology Information. PubChem Compound Summary for CID 23667635, Sodium periodate. https://pubchem.ncbi.nlm.nih.gov/compound/Sodium-periodate (accessed May 11, 2022).

[ref37] Liimatainen H.; Sirviö J.; Pajari H.; Hormi O.; Niinimäki J. Regeneration and Recycling of Aqueous Periodate Solution in Dialdehyde Cellulose Production. J. Wood Chem. Technol. 2013, 33, 258–266. 10.1080/02773813.2013.783076.

[ref38] Arndt S.; Weis D.; Donsbach K.; Waldvogel S. R. The “Green” Electrochemical Synthesis of Periodate. Angew. Chem., Int. Ed. Engl. 2020, 59, 8036–8041. 10.1002/anie.202002717. 32181555 PMC7317427

[ref39] Janssen L. J. J.; Blijlevens M. H. A. Electrochemical Oxidation of Iodate to Periodate. Electrochim. Acta 2003, 48, 3959–3964. 10.1016/S0013-4686(03)00535-8.

[ref40] Koprivica S.; Siller M.; Hosoya T.; Roggenstein W.; Rosenau T.; Potthast A. Regeneration of Aqueous Periodate Solutions by Ozone Treatment: A Sustainable Approach for Dialdehyde Cellulose Production. ChemSusChem 2016, 9, 825–833. 10.1002/cssc.201501639. 26990816

[ref41] Plappert S. F.; Quraishi S.; Pircher N.; Mikkonen K. S.; Veigel S.; Klinger K. M.; Potthast A.; Rosenau T.; Liebner F. W. Transparent, Flexible, and Strong 2,3-Dialdehyde Cellulose Films with High Oxygen Barrier Properties. Biomacromolecules 2018, 19, 2969–2978. 10.1021/acs.biomac.8b00536. 29757619 PMC6041771

[ref42] Amer H.; Nypelö T.; Sulaeva I.; Bacher M.; Henniges U.; Potthast A.; Rosenau T. Synthesis and Characterization of Periodate-Oxidized Polysaccharides: Dialdehyde Xylan (DAX). Biomacromolecules 2016, 17, 2972–2980. 10.1021/acs.biomac.6b00777. 27529432

[ref43] Kim U.-J.; Kuga S.; Wada M.; Okano T.; Kondo T. Periodate Oxidation of Crystalline Cellulose. Biomacromolecules 2000, 1, 488–492. 10.1021/bm0000337. 11710141

[ref44] Spedding H. 628. Infrared spectra of periodate-oxidised cellulose. J. Chem. Soc. 1960, 1960, 3147–3152. 10.1039/JR9600003147.

[ref100] Ahn K.; Zaccaron S.; Zwirchmayr N. S.; Hettegger H.; Hofinger H.; Bacher M.; Henniges U.; Hosoya T.; Potthast A.; Rosenau T. Yellowing and brightness reversion of celluloses: CO or COOH, who is the culprit?. Cellulose 2019, 26, 429–444. 10.1007/s10570-018-2200-x.

[ref45] Hosoya T.; Bacher M.; Potthast A.; Elder T.; Rosenau T. Insights into Degradation Pathways of Oxidized Anhydroglucose Units in Cellulose by β-Alkoxy-Elimination: A Combined Theoretical and Experimental Approach. Cellulose 2018, 25, 3797–3814. 10.1007/s10570-018-1835-y.

[ref46] Dash R.; Elder T.; Ragauskas A. J. Grafting of Model Primary Amine Compounds to Cellulose Nanowhiskers through Periodate Oxidation. Cellulose 2012, 19, 2069–2079. 10.1007/s10570-012-9769-2.

[ref47] Tsuchida S.; Takahashi R.; Yabe K.; Hamaue N.; Aoki T. A Simple Method for Preparing a Diamino Cellulose Disk from a Dialdehyde Cellulose Disk by Reductive Amination Using 2-Picoline-Borane. Cellulose 2022, 29, 3025–3033. 10.1007/s10570-022-04503-y.

[ref48] Bandi R.; Alle M.; Dadigala R.; Park C.-W.; Han S.-Y.; Kwon G.-J.; Kim J.-C.; Lee S.-H. Integrating the High Peroxidase Activity of Carbon Dots with Easy Recyclability: Immobilization on Dialdehyde Cellulose Nanofibrils and Cholesterol Detection. Appl. Mater. Today 2022, 26, 101286 10.1016/j.apmt.2021.101286.

[ref49] Guigo N.; Mazeau K.; Putaux J.-L.; Heux L. Surface Modification of Cellulose Microfibrils by Periodate Oxidation and Subsequent Reductive Amination with Benzylamine: A Topochemical Study. Cellulose 2014, 21, 4119–4133. 10.1007/s10570-014-0459-0.

[ref50] Ruan C.; Strømme M.; Lindh J. A Green and Simple Method for Preparation of an Efficient Palladium Adsorbent Based on Cysteine Functionalized 2,3-Dialdehyde Cellulose. Cellulose 2016, 23, 2627–2638. 10.1007/s10570-016-0976-0.

[ref51] Lindh J.; Ruan C.; Strømme M.; Mihranyan A. Preparation of Porous Cellulose Beads via Introduction of Diamine Spacers. Langmuir 2016, 32, 5600–5607. 10.1021/acs.langmuir.6b01288. 27181427

[ref52] Ruan C.-Q.; Strømme M.; Lindh J. Preparation of Porous 2,3-Dialdehyde Cellulose Beads Crosslinked with Chitosan and Their Application in Adsorption of Congo Red Dye. Carbohydr. Polym. 2018, 181, 200–207. 10.1016/j.carbpol.2017.10.072. 29253964

[ref53] Kim U.-J.; Kim H. J.; Choi J. W.; Kimura S.; Wada M. Cellulose-Chitosan Beads Crosslinked by Dialdehyde Cellulose. Cellulose 2017, 24, 5517–5528. 10.1007/s10570-017-1528-y.

[ref54] Jin L.; Li W.; Xu Q.; Sun Q. Amino-Functionalized Nanocrystalline Cellulose as an Adsorbent for Anionic Dyes. Cellulose 2015, 22, 2443–2456. 10.1007/s10570-015-0649-4.

[ref55] Sirviö J. A.; Liimatainen H.; Niinimäki J.; Hormi O. Sustainable Packaging Materials Based on Wood Cellulose. RSC Adv. 2013, 3, 16590–16596. 10.1039/C3RA43264E.

[ref56] Sato S.; Sakamoto T.; Miyazawa E.; Kikugawa Y. One-Pot Reductive Amination of Aldehydes and Ketones with α-Picoline-Borane in Methanol, in Water, and in Neat Conditions. Tetrahedron 2004, 60, 7899–7906. 10.1016/j.tet.2004.06.045.

[ref57] Ruhaak L. R.; Steenvoorden E.; Koeleman C. A. M.; Deelder A. M.; Wuhrer M. 2-Picoline-Borane: A Non-Toxic Reducing Agent for Oligosaccharide Labeling by Reductive Amination. Proteomics 2010, 10, 2330–2336. 10.1002/pmic.200900804. 20391534

[ref58] Potthast A.; Schiehser S.; Rosenau T.; Kostic M. Oxidative Modifications of Cellulose in the Periodate System – Reduction and Beta-Elimination Reactions. Holzforschung 2009, 63, 12–17. 10.1515/HF.2009.108.

[ref59] Münster L.; Vícha J.; Klofáč J.; Masař M.; Kucharczyk P.; Kuřitka I. Stability and Aging of Solubilized Dialdehyde Cellulose. Cellulose 2017, 24, 2753–2766. 10.1007/s10570-017-1314-x.

[ref60] Bobbitt J. M. Periodate Oxidation of Carbohydrates. Advances in Carbohydrate Chemistry; Wolfrom M. L., Tipson R. S., Eds.; Academic Press, 1956; Vol. 11, pp 1–41. 10.1016/s0096-5332(08)60115-0 13469627

[ref61] Maekawa E.; Koshijima T. Preparation and Structural Consideration of Nitrogen-Containing Derivatives Obtained from Dialdehyde Celluloses. J. Appl. Polym. Sci. 1991, 42, 169–178. 10.1002/app.1991.070420120.

[ref62] Koso T.; Rico del Cerro D.; Heikkinen S.; Nypelö T.; Buffiere J.; Perea-Buceta J. E.; Potthast A.; Rosenau T.; Heikkinen H.; Maaheimo H.; Isogai A.; Kilpeläinen I.; King A. W. T. 2D Assignment and Quantitative Analysis of Cellulose and Oxidized Celluloses Using Solution-State NMR Spectroscopy. Cellulose 2020, 27, 7929–7953. 10.1007/s10570-020-03317-0.

[ref63] King A. W. T.; Mäkelä V.; Kedzior S. A.; Laaksonen T.; Partl G. J.; Heikkinen S.; Koskela H.; Heikkinen H. A.; Holding A. J.; Cranston E. D.; Kilpeläinen I. Liquid-State NMR Analysis of Nanocelluloses. Biomacromolecules 2018, 19, 2708–2720. 10.1021/acs.biomac.8b00295. 29614220

[ref64] Jusner P.; Bacher M.; Simon J.; Bausch F.; Khaliliyan H.; Schiehser S.; Sumerskii I.; Schwaiger E.; Potthast A.; Rosenau T. Analyzing the Effects of Thermal Stress on Insulator Papers by Solid-State 13C NMR Spectroscopy. Cellulose 2022, 29, 1081–1095. 10.1007/s10570-021-04338-z.

[ref65] Becker M.; Zweckmair T.; Forneck A.; Rosenau T.; Potthast A.; Liebner F. Evaluation of Different Derivatisation Approaches for Gas Chromatographic–Mass Spectrometric Analysis of Carbohydrates in Complex Matrices of Biological and Synthetic Origin. J. Chromatogr. A 2013, 1281, 115–126. 10.1016/j.chroma.2013.01.053. 23399001

[ref66] Sundheq A.; Sundherg K.; Lillandt C.; Holmhom B. Determination of Hemicelluloses and Pectins in Wood and Pulp Fibres by Acid Methanolysis and Gas Chromatography. Nord. Pulp Pap. Res. J. 1996, 11, 216–219. 10.3183/npprj-1996-11-04-p216-219.

[ref67] Siller M.; Ahn K.; Pircher N.; Rosenau T.; Potthast A. Dissolution of Rayon Fibers for Size Exclusion Chromatography: A Challenge. Cellulose 2014, 21, 3291–3301. 10.1007/s10570-014-0356-6.

[ref68] Potthast A.; Radosta S.; Saake B.; Lebioda S.; Heinze T.; Henniges U.; Isogai A.; Koschella A.; Kosma P.; Rosenau T.; Schiehser S.; Sixta H.; Strlič M.; Strobin G.; Vorwerg W.; Wetzel H. Comparison Testing of Methods for Gel Permeation Chromatography of Cellulose: Coming Closer to a Standard Protocol. Cellulose 2015, 22, 1591–1613. 10.1007/s10570-015-0586-2.

[ref69] Simon J.; Tsetsgee O.; Iqbal N. A.; Sapkota J.; Ristolainen M.; Rosenau T.; Potthast A. A Fast Method to Measure the Degree of Oxidation of Dialdehyde Celluloses Using Multivariate Calibration and Infrared Spectroscopy. Carbohydr. Polym. 2022, 278, 118887 10.1016/j.carbpol.2021.118887. 34973725

[ref70] Simon J.; Tsetsgee O.; Iqbal N. A.; Sapkota J.; Ristolainen M.; Rosenau T.; Potthast A. Fourier Transform and near Infrared Dataset of Dialdehyde Celluloses Used to Determine the Degree of Oxidation with Chemometric Analysis. Data Brief 2022, 40, 107757 10.1016/j.dib.2021.107757. 35005146 PMC8718732

[ref72] Zhao H.; Heindel N. D. Determination of Degree of Substitution of Formyl Groups in Polyaldehyde Dextran by the Hydroxylamine Hydrochloride Method. Pharm. Res. 1991, 08, 400–402. 10.1023/A:1015866104055. 1711201

[ref73] Yuan S.; Tyufekchiev M.; Timko M. T.; Schmidt-Rohr K. Direct Quantification of the Degree of Polymerization of Hydrolyzed Cellulose by Solid-State NMR Spectroscopy. Cellulose 2022, 29, 2131–2144. 10.1007/s10570-022-04433-9.

[ref74] Liu P.; Pang B.; Dechert S.; Zhang X. C.; Andreas L. B.; Fischer S.; Meyer F.; Zhang K. Structure Selectivity of Alkaline Periodate Oxidation on Lignocellulose for Facile Isolation of Cellulose Nanocrystals. Angew. Chem., Int. Ed. Engl. 2020, 59, 3218–3225. 10.1002/anie.201912053. 31692150 PMC7027850

[ref75] Carraher C. E. Molecular Weight of Polymers. Introduction to Polymer Chemistry; Taylor & Francis, 2012; pp 71–72.

[ref76] Nose T.; Chu B. Light Scattering. Polymer Science: A Comprehensive Reference; Matyjaszewski K., Möller M., Eds.; Elsevier: Amsterdam, 2012; pp 301–329.

