# Supplementary material for: Reductive Amination of Dialdehyde Cellulose: Access to Renewable Thermoplastics
Source: Biomacromolecules. 2022 Dec 21;24(1):166–77. doi: 10.1021/acs.biomac.2c01022 (PMC9832504; doi:10.1021/acs.biomac.2c01022)
Supplement: Supplementary file 1 — bm2c01022_si_001.pdf [file bm2c01022_si_001.pdf]

SUPPLEMENTARY MATERIAL

Reductive Amination of Dialdehyde Cellulose: Access to Renewable Thermoplastics

Jonas Simon[a], Lukas Fliri[b], Janak Sapkota[c], Matti Ristolainen[c], Stephen A. Miller[d], Michael Hummel[b],Thomas Rosenau[a,*], Antje Potthast[a,*]

[a] Department of Chemistry, Institute of Chemistry of Renewable Resources, University of Natural Resources and Life Sciences Vienna (BOKU), Konrad-Lorenz-Strasse 24, 3430 Tulln, Austria

[b] Department of Bioproducts and Biosystems, Aalto University, 0076 Aalto, Finland

[c] NE Research Center, UPM Pulp Research and Innovations, 53200 Lappeenranta, Finland

[d] The George and Josephine Butler Laboratory for Polymer Research, Department of Chemistry, University of Florida, Gainesville, Florida 32611-7200, USA

*Corresponding author: antje.potthast@boku.ac.at

Table of contents

1) Overview of the isolated diamino celluloses from 39 %-oxidized softwood kraft pulp 3
2) Characterization of the cellulosic starting materials 4
2.1) Softwood Kraft Pulp 4
2.2) Microcrystalline cellulose (MCC, Avicel® PH-101) 7
3) Cellulose solution state NMR data 8
3.1) Microcrystalline cellulose (MCC, Avicel® PH-101) starting material 8
3.2) Dialdehyde cellulose (MCC, DO = 8 %) 10
3.3) Dialcohol cellulose from MCC – DAC (DO = 8 %) 12
3.4) Dityramine cellulose from MCC – DAC (DO = 8 %) 13
3.5) Diethanolamine cellulose from MCC – DAC (DO = 8 %) 15
3.6) Dibutylamine cellulose from MCC – DAC (DO = 8 %) 16
3.7) Dihexylamine cellulose from MCC – DAC (DO = 8 %) 18
3.8) Dianilineamine cellulose from MCC – DAC (DO = 8 %) 19
3.9) Softwood kraft pulp (SKP) starting material 21
3.10) Dialdehyde cellulose (SKP, DO = 39 %) 22
3.11) Diethanolamine cellulose from SKP – DAC (DO = 39 %) 23
3.12) Dianilineamine cellulose from SKP – DAC (DO = 39 %) 24
4) FTIR spectra for the isolated diamino celluloses 26
4.1) Diamine celluloses obtained from microcrystalline cellulose (MCC, Avicel® PH-101) (DO = 8 %) 26
4.2) Diamine celluloses obtained from partially oxidized softwood kraft pulp (DO = 39 %) 29
5) GPC/MALLS-RI data 32
5.1) Diamine celluloses obtained from microcrystalline cellulose (MCC, Avicel® PH-101) (DO = 8 %) 32
5.2) Diamine celluloses obtained from partially oxidized softwood kraft pulp (DO = 39 %) 34
6) Derivation of the formula to calculate the aldehyde content from titration data 37
7) Derivation of the formula to calculate the degree of substitution from elemental analysis data 38
References 39

1) Overview of the isolated diamino celluloses from 39 %-oxidized softwood kraft pulp

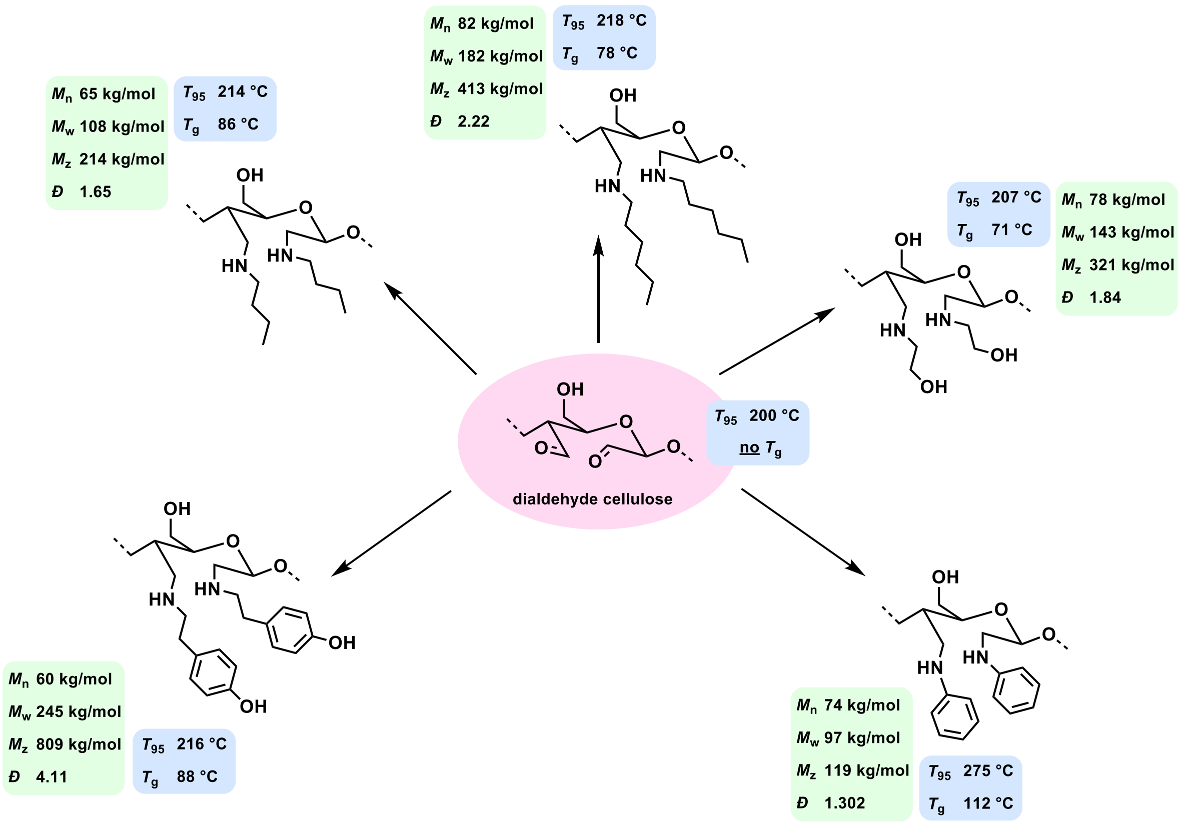

Figure S1. Thermal data and GPC-MALLS statistical moments of all our synthesized diamino celluloses from 39 %-oxidized softwood kraft pulp.

2) Characterization of the cellulosic starting materials

2.1) Softwood kraft pulp

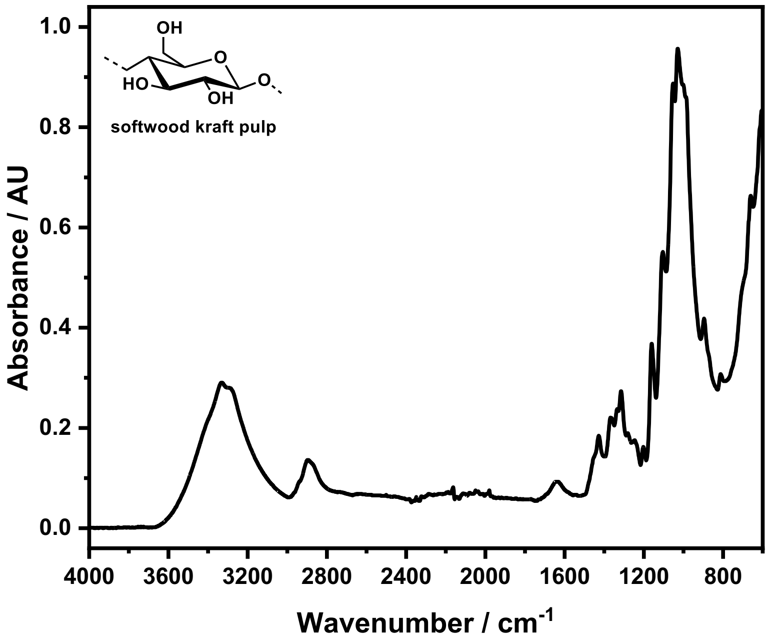

Figure S2. Normalized Fourier-transform infrared spectra of untreated softwood kraft pulp.

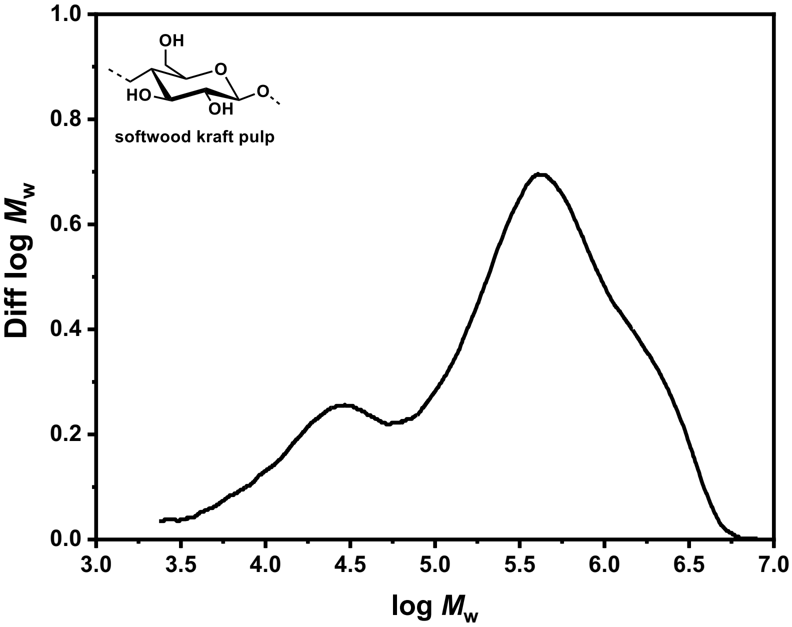

Figure S3. Molecular weight distribution of untreated softwood kraft pulp.

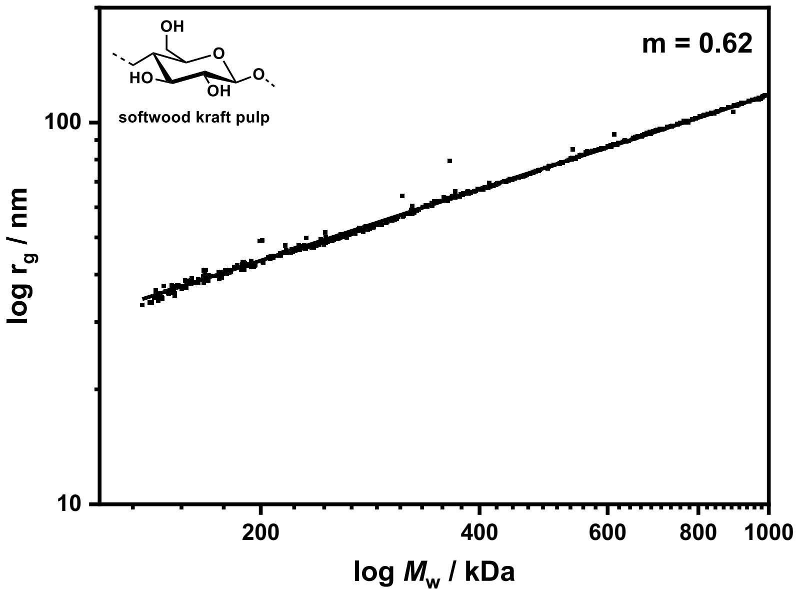

Figure S4. Conformation plot of untreated softwood kraft pulp. The slope of the linear regression of the light scattering data is 0.62.

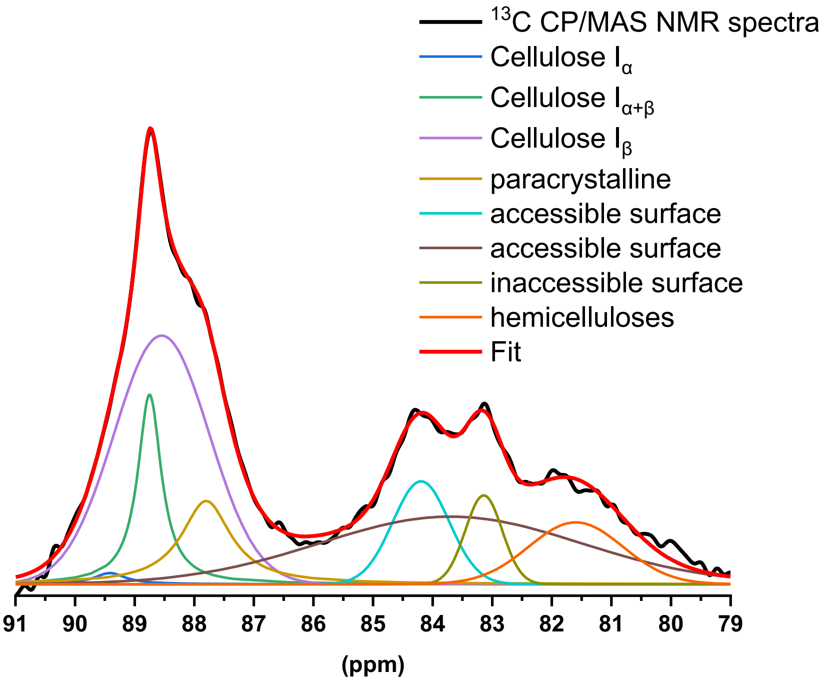

Figure S5. C4 resonance in ^{13}C CP/MAS NMR spectrum of untreated softwood kraft pulp deconvoluted according to Jusner et al.[1]

Table S1. Contributors to the C4 resonance in ^{13}C CP/MAS NMR spectrum of untreated softwood kraft pulp.

	(ppm)	Width (ppm)	Integral [91-79ppm] (%)
**Cellulose I_{α}**	89.42	0.64	0.72
**Cellulose $I_{\alpha+\beta}$**	88.75	0.48	9.47
**Cellulose I_{β}**	87.80	1.01	8.49
**Paracrystalline**	88.55	1.88	34.35
**Accessible surface I**	84.19	1.12	8.45
**Inaccessible surface**	83.70	5.17	25.29
**Accessible surface II**	83.14	0.73	4.77
**Hemicelluloses**	81.60	1.86	8.46
**Sum**			**100.00**

Table S2. Amounts of carbohydrates expressed as µg/mg determined by methanolysis and gas chromatography according to Sundberg et al.[2]

	Ara	Xyl	Man	Gal	Glc
**Softwood kraft pulp**	7.72	32.31	16.30	2.50	27.73

2.2) Microcrystalline cellulose (MCC, Avicel® PH-101)

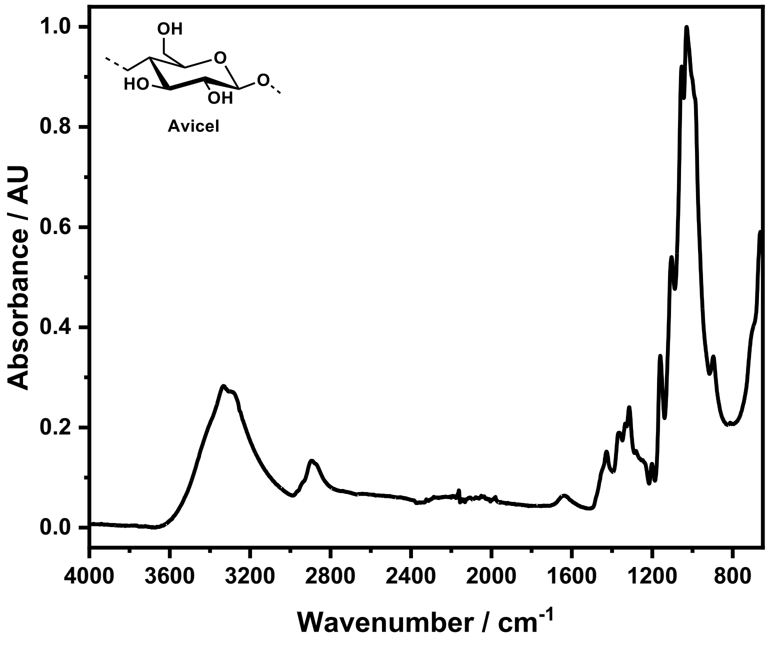

Figure S6. Normalized Fourier-transform infrared spectra of the Avicel® PH-101 starting material.

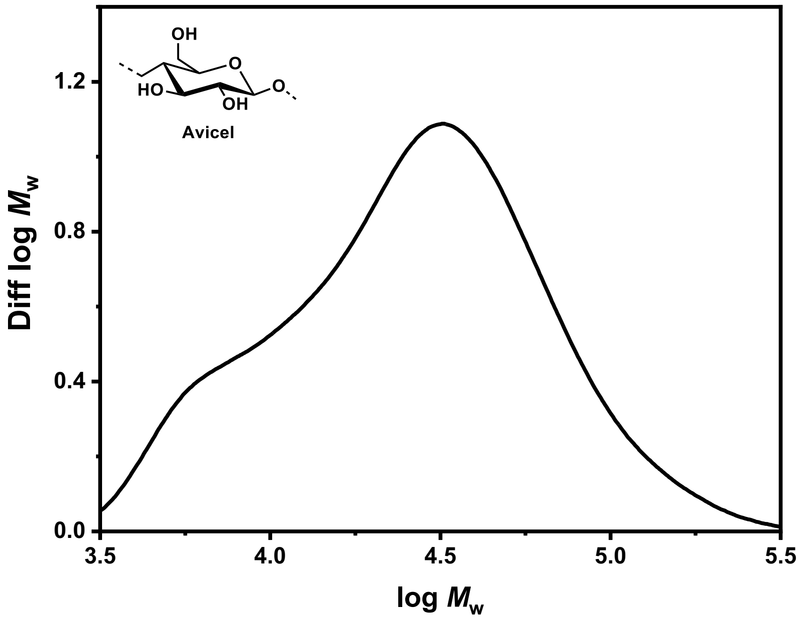

Figure S7. Molecular weight distribution of the Avicel® PH-101 starting material.

3) Cellulose solution state NMR data

3.1) Microcrystalline cellulose (MCC, Avicel® PH-101) starting material

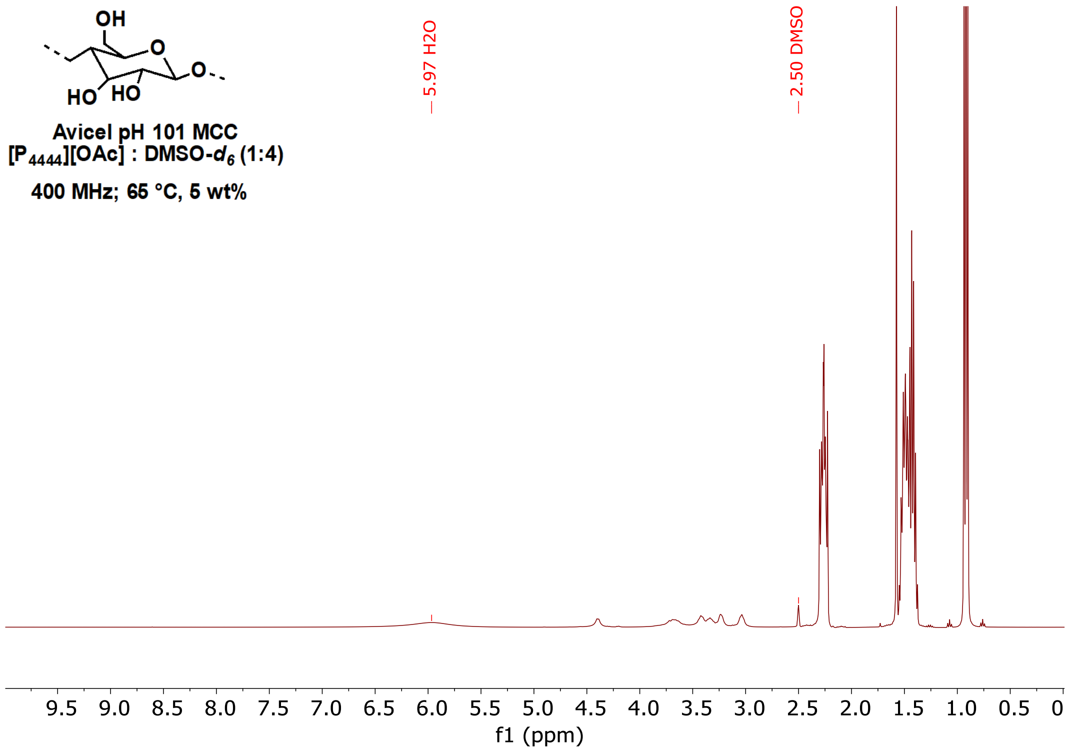

Figure S8. Quantitative ^{1}H NMR spectrum ($[P_{4444}][OAc]$: DMSO-d_6 (v/v = 1 : 4); 400 MHz; 65°C) of the Avicel® PH-101 starting material (5 wt%). Full spectral area with electrolyte and water resonances is shown.

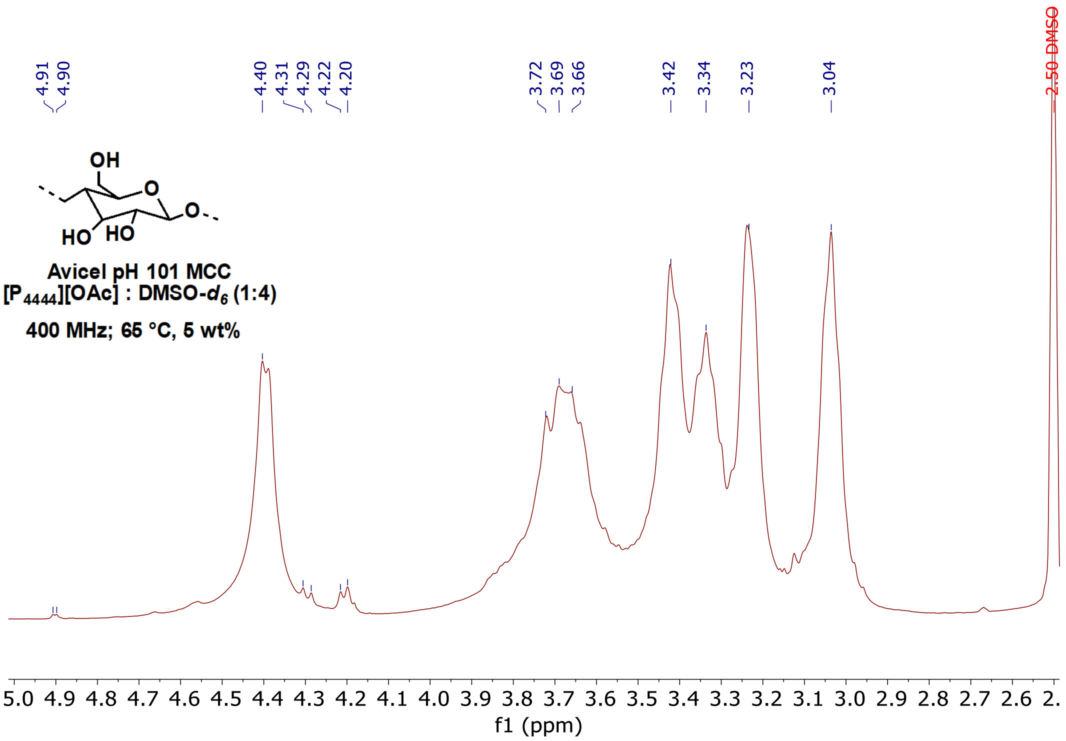

Figure S9. Quantitative ^{1}H NMR spectrum ($[P_{4444}][OAc]$: DMSO-d_6 (v/v = 1 : 4); 400 MHz; 65°C) of the Avicel® PH-101 starting material (5 wt%). Zoom into the polysaccharide region is shown. Resonances of end groups (4.9 and 4.3 ppm) and minor hemicellulose impurities (xylane C1-H at 4.2 ppm) visible.

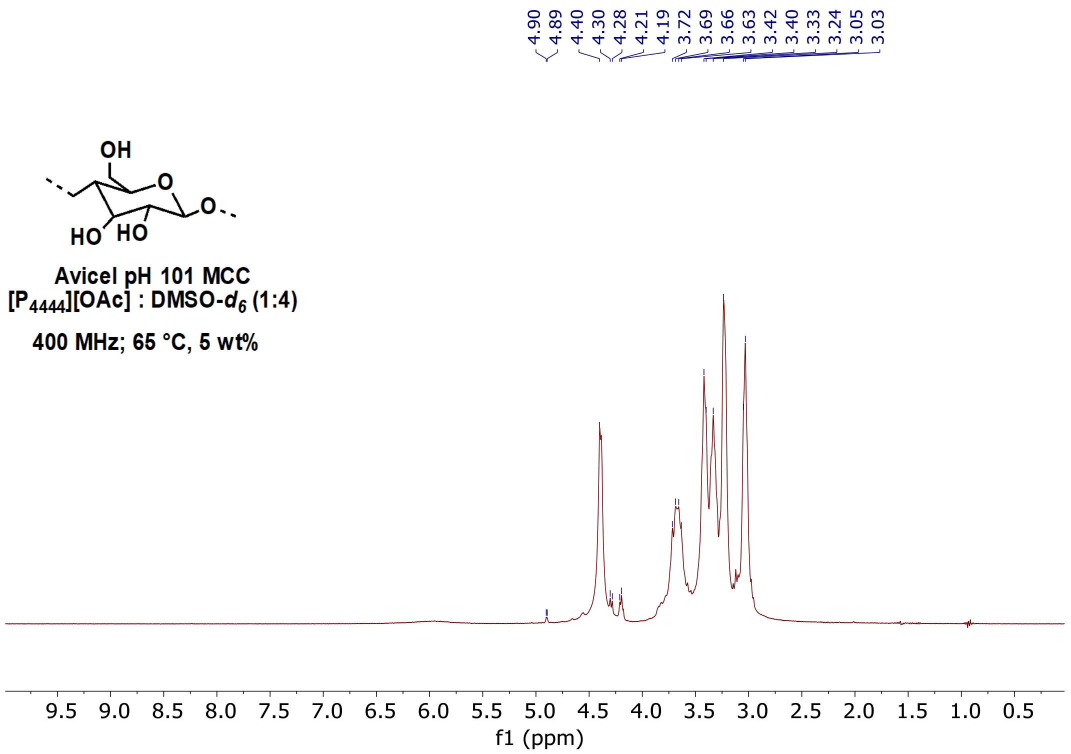

Figure S10. Diffusion edited ^{1}H NMR spectrum ($[P_{4444}][OAc]$: DMSO-d_6 (v/v = 1 : 4); 400 MHz; 65°C) of the Avicel® PH-101 starting material (5 wt%). Full spectral area is shown. Resonances of end groups (4.9 and 4.3 ppm) and minor hemicellulose impurities (xylane C1-H at 4.2 ppm) visible.

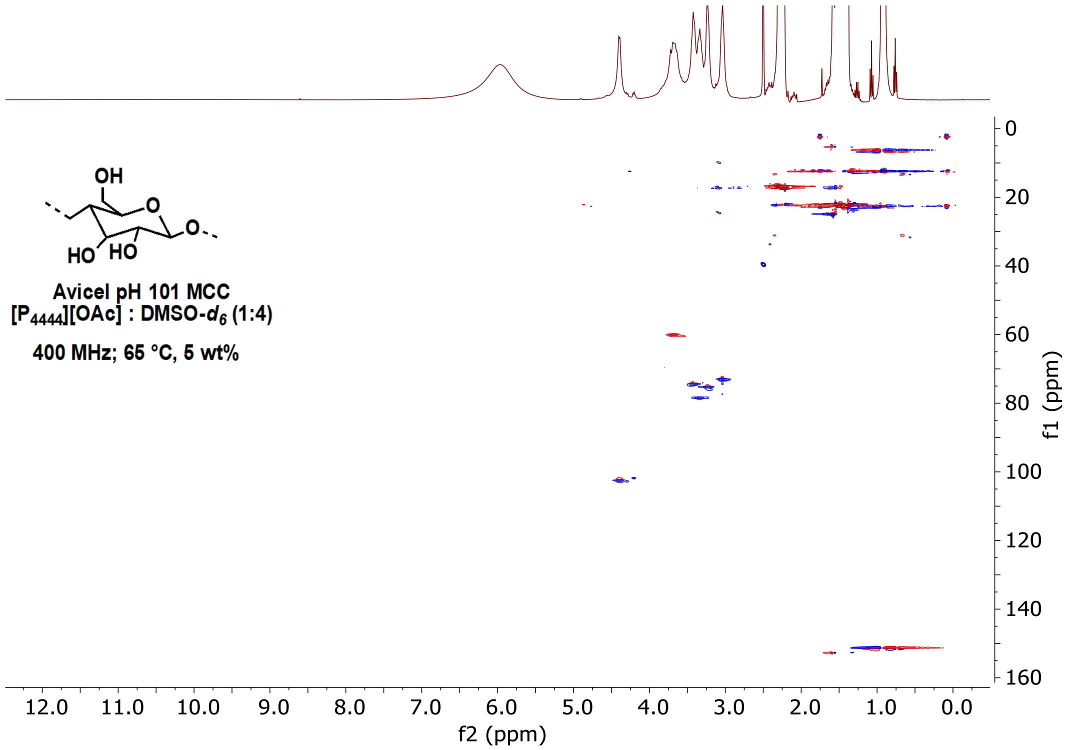

Figure S11. Multiplicity-edited HSQC spectrum ($[P_{4444}][OAc]$:DMSO-d_6 (v/v = 1 : 4); 400 MHz ^{1}H frequency; 65°C) of the Avicel® PH-101 starting material (5 wt%). CH_2 resonances are shown in red, CH / CH_3 signals are shown in blue. On top the quantitative ^{1}H spectrum is inserted. The full spectral with is shown, highlighting the intensive residual electrolyte signals and phasing artifacts in the respective aliphatic regions.

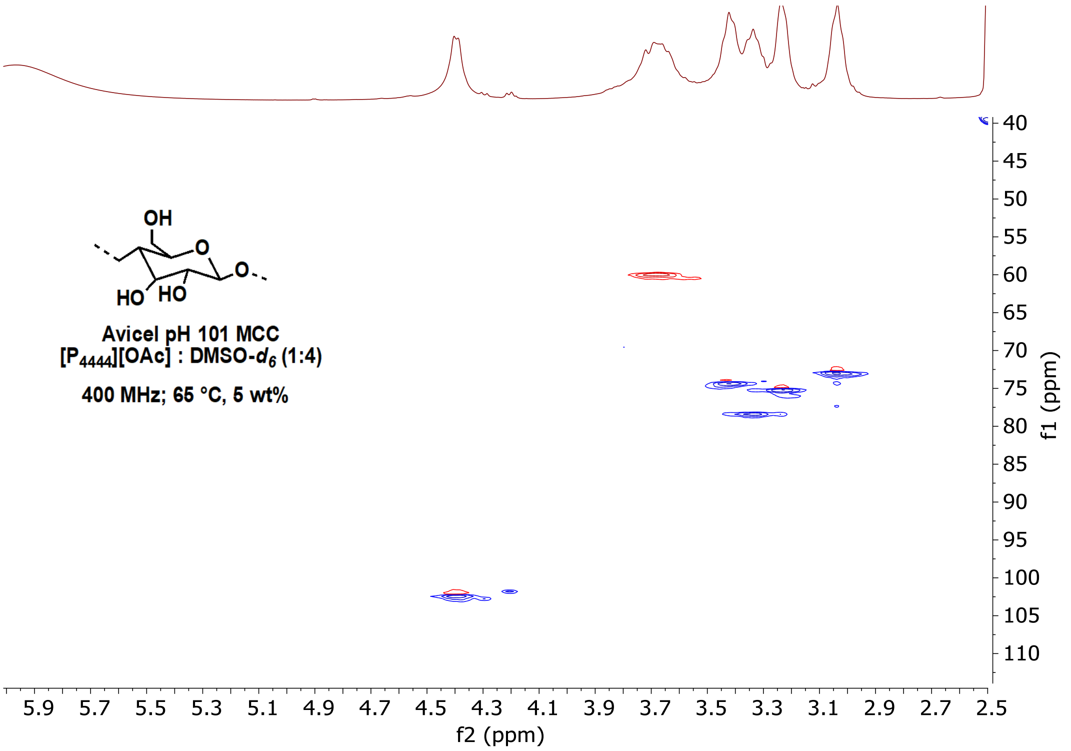

Figure S12. Multiplicity-edited HSQC spectrum ([P_{4444}][OAc]:DMSO-d_6 (v/v = 1 : 4); 400 MHz ^{1}H frequency; 65°C) of the Avicel® PH-101 starting material (5 wt%). CH_2 resonances are shown in red, CH / CH_3 signals are shown in blue. On top the quantitative ^{1}H spectrum is inserted. Only the zoom into the polysaccharide region is shown.

3.2) Dialdehyde cellulose (MCC, DO = 8 %)

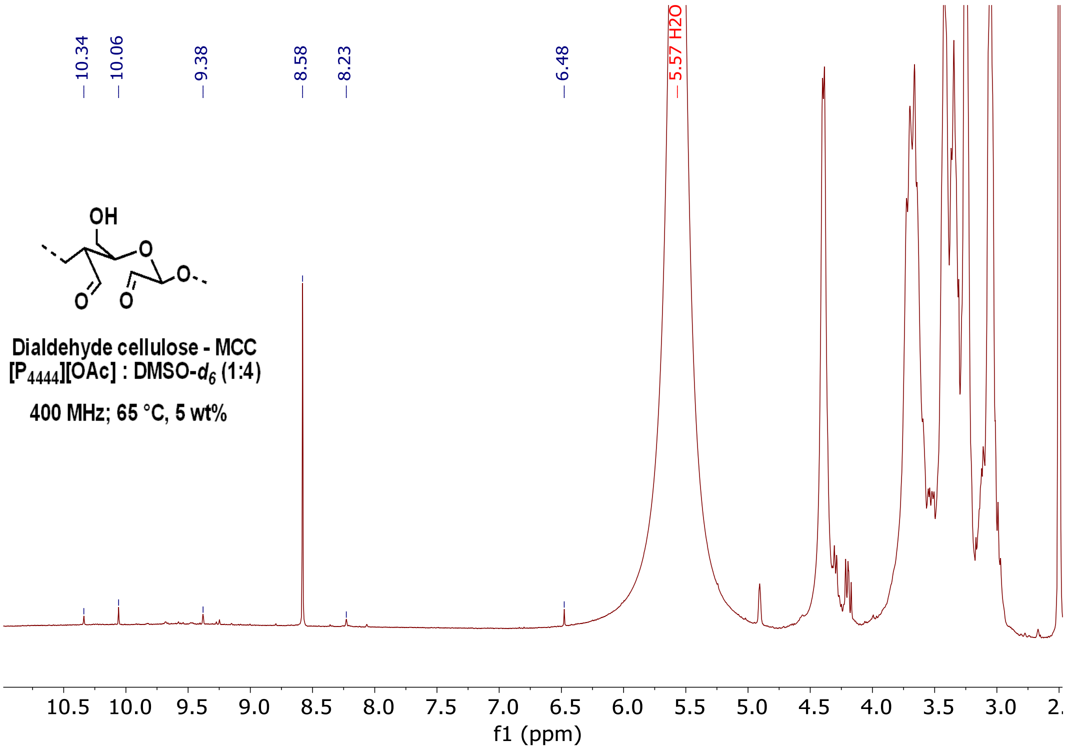

Figure S13. Quantitative ^{1}H NMR spectrum ([P_{4444}][OAc] : DMSO-d_6 (v/v = 1 : 4); 400 MHz; 65°C) of MCC - dialdehyde cellulose (DO = 8 %; 5 wt%). Zoom into the polysaccharide and degradation product regions is shown. Strong degradation to low molecular weight compounds is observed. Major degradation product is formiate ($HCOO^-$), exemplified by the strong peak at 8.58 ppm.

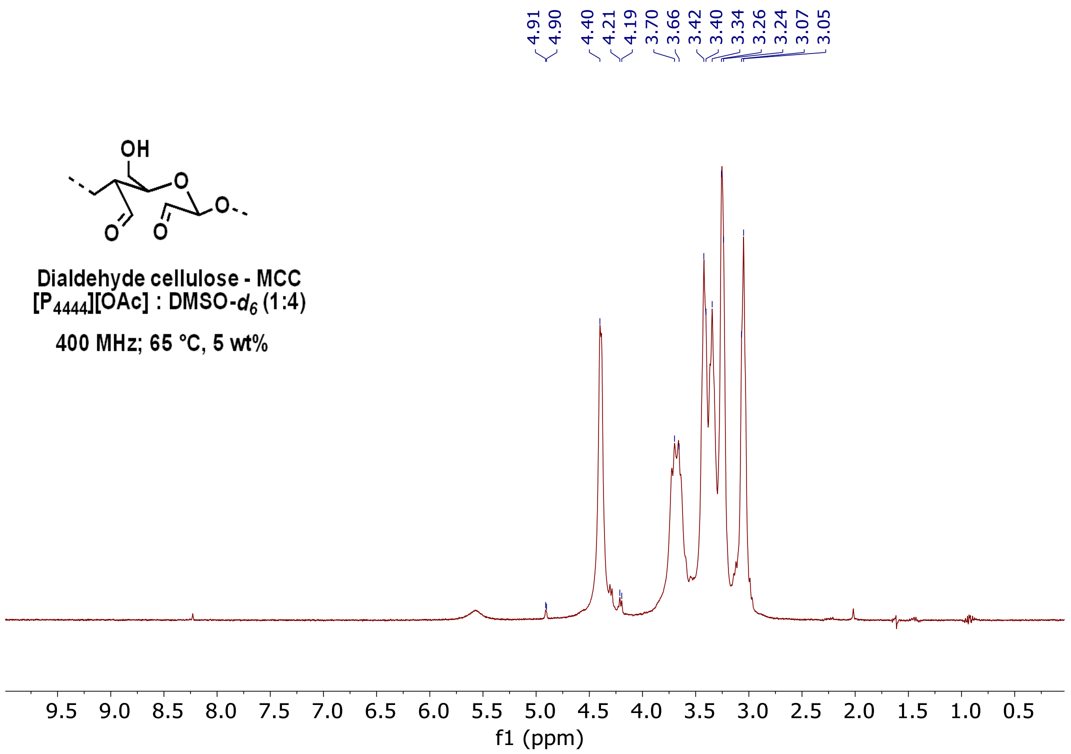

Figure S14. Diffusion edited ^{1}H NMR spectrum ([P_{4444}][OAc] : DMSO-d_6 (v/v = 1 : 4); 400 MHz; 65°C) of MCC - dialdehyde cellulose (DO = 8 %; 5 wt%). The residual polymeric cellulose components show increase of end group intensities, suggesting significant depolymerization.

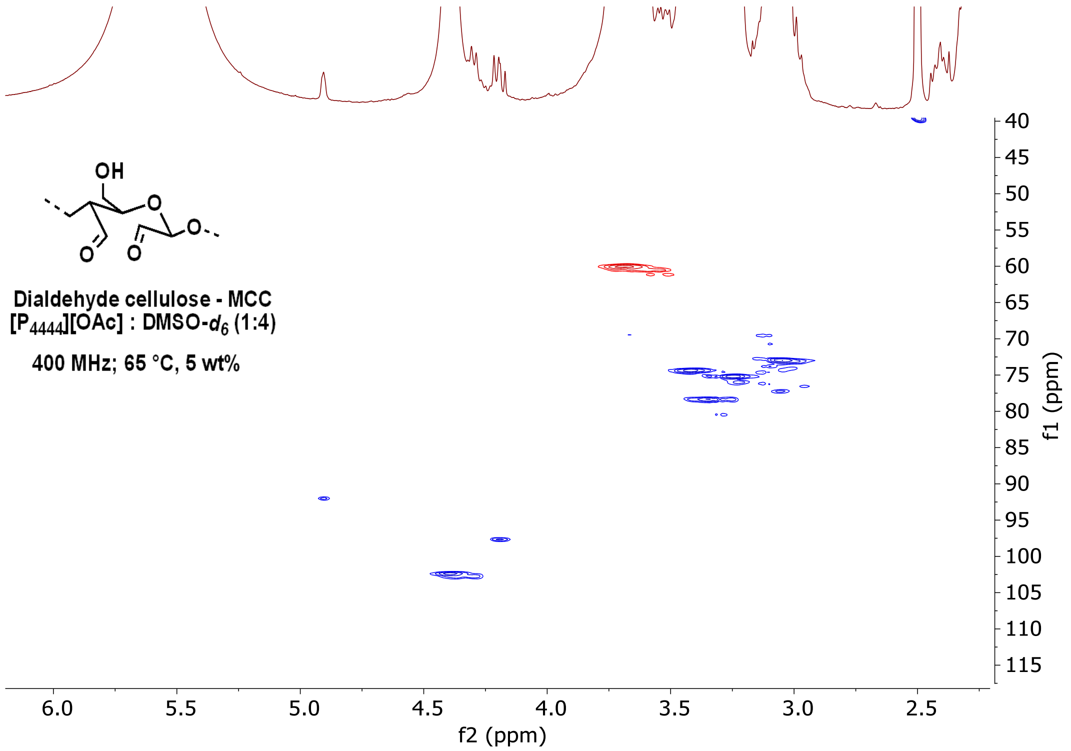

Figure S15. Multiplicity-edited HSQC spectrum ([P_{4444}][OAc]:DMSO-d_6 (v/v = 1 : 4); 400 MHz ^{1}H frequency; 65°C) of MCC - dialdehyde cellulose (DO = 8 %; 5 wt%). CH_2 resonances are shown in red, CH / CH_3 signals are shown in blue. On top the quantitative ^{1}H spectrum is inserted. Zoom into the polysaccharide region is shown.

3.3) Dialcohol cellulose from MCC – DAC (DO = 8 %)

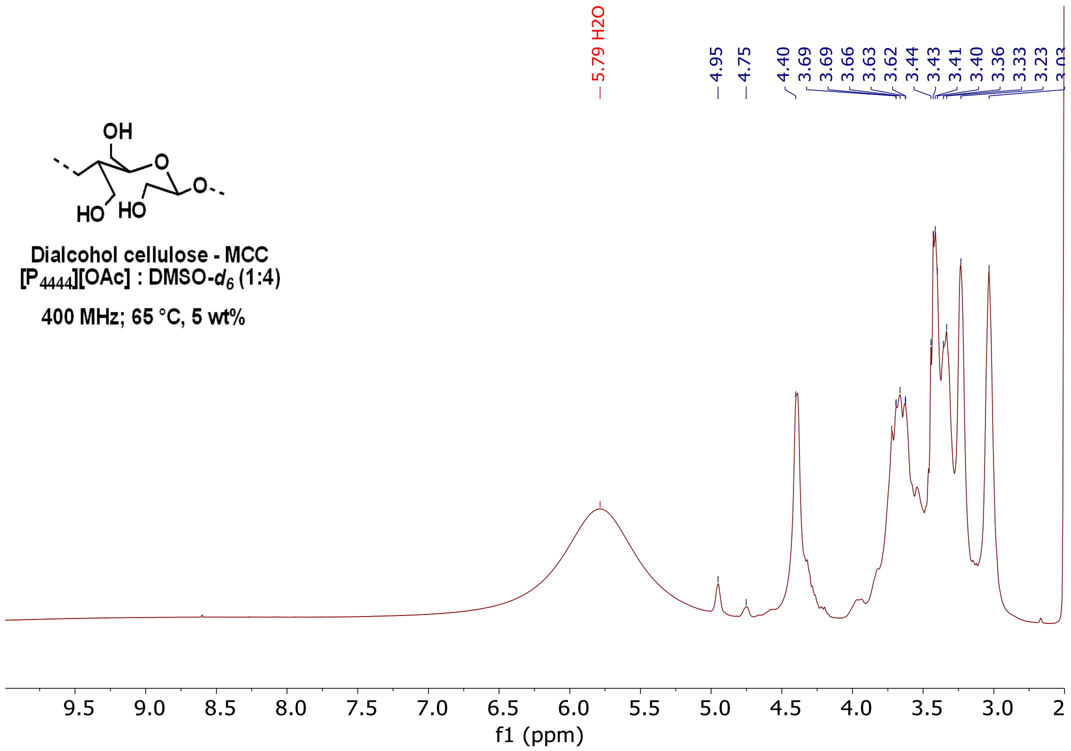

Figure S16. Quantitative ^{1}H NMR spectrum ([P_{4444}][OAc] : DMSO-d_6 (v/v = 1 : 4); 400 MHz; 65°C) of dialcohol cellulose obtained from MCC-DAC (DO = 8 %; 5 wt%). Zoom into the polysaccharide and degradation product regions is shown. Only minimal degradation to formiate (8.58 ppm) is observed, suggesting a stabilisation of the material towards more alkaline conditions.

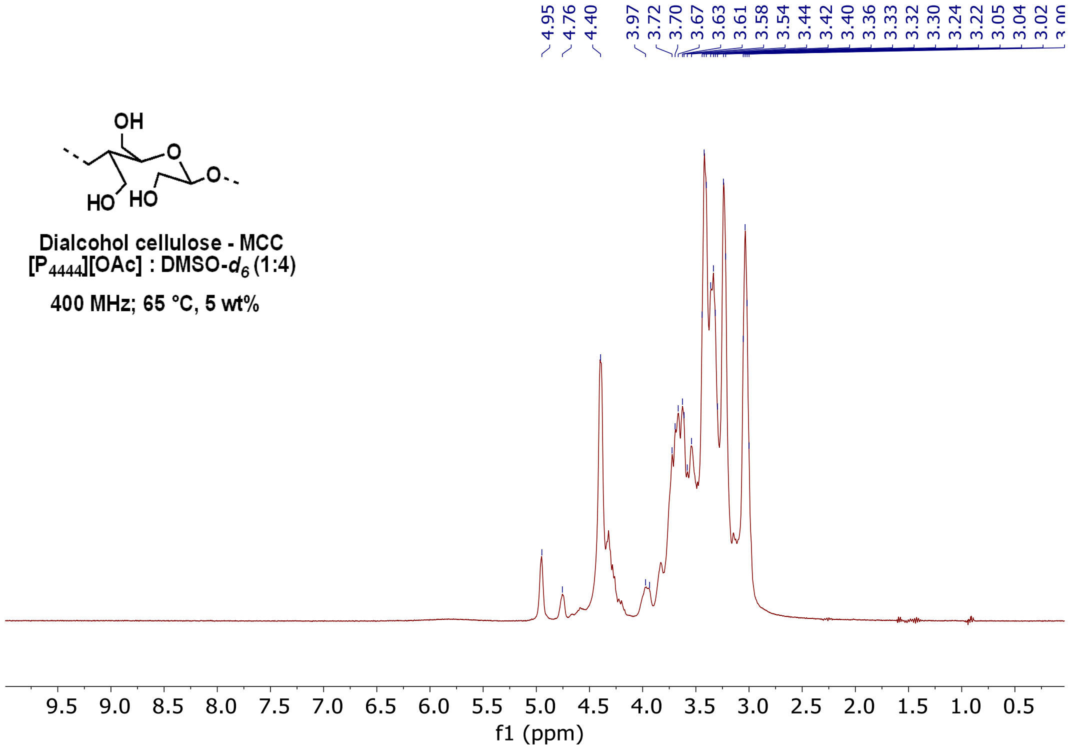

Figure S17. Diffusion edited ^{1}H NMR spectrum ([P_{4444}][OAc] : DMSO-d_6 (v/v = 1 : 4); 400 MHz; 65°C) of dialcohol cellulose obtained from MCC-DAC (DO = 8 %; 5 wt%). Strong peak superposition with residual cellulose resonances. Characteristic dialcohol cellulose peaks for acetal C1-H at 4.95 and 4.76 ppm.

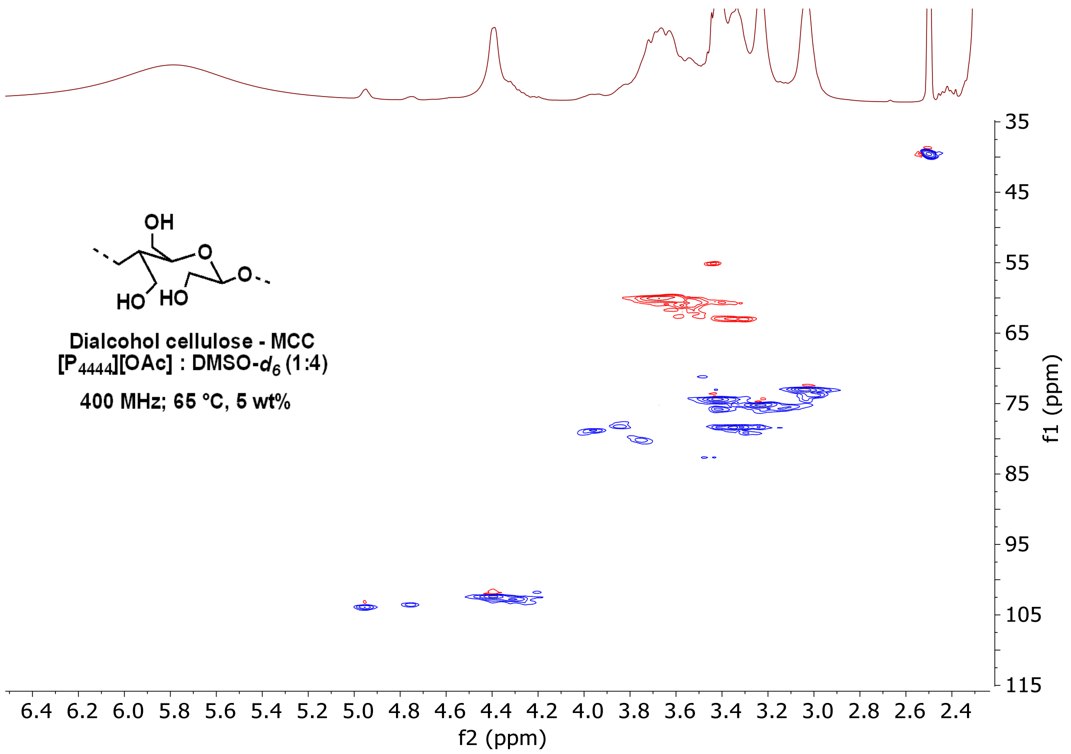

Figure S18. Multiplicity-edited HSQC spectrum ([P_{4444}][OAc]:DMSO-*d₆* (v/v = 1 : 4); 400 MHz ^{1}H frequency; 65°C) of dialcohol cellulose obtained from MCC-DAC (DO = 8 %; 5 wt%). CH_2 resonances are shown in red, CH / CH_3 signals are shown in blue. On top the quantitative ^{1}H spectrum is inserted. Zoom into the polysaccharide region is shown.

3.4) Dityramine cellulose from MCC – DAC (DO = 8 %)

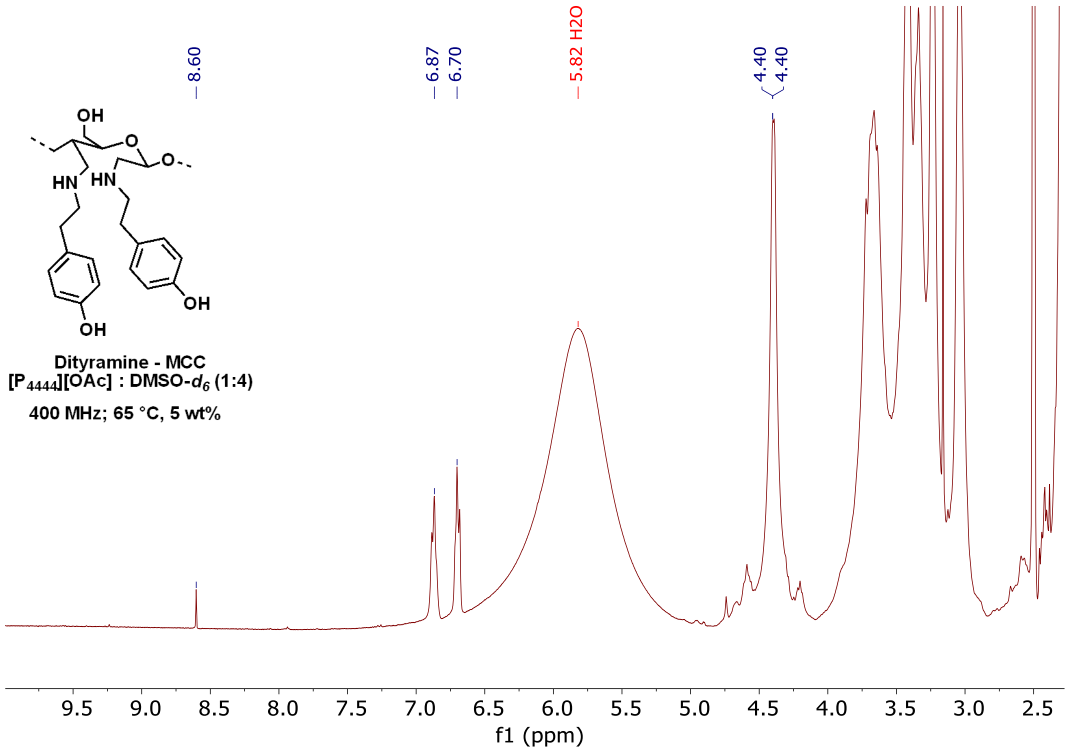

Figure S19. Quantitative ^{1}H NMR spectrum ([P_{4444}][OAc] : DMSO-*d₆* (v/v = 1 : 4); 400 MHz; 65°C) of dityramine cellulose obtained from MCC-DAC (DO = 8 %; 5 wt%). Zoom into the polysaccharide and degradation product regions is shown. Peaks of the newly introduced aromatic moieties and considerable degradation to formiate (8.60 ppm) visible.

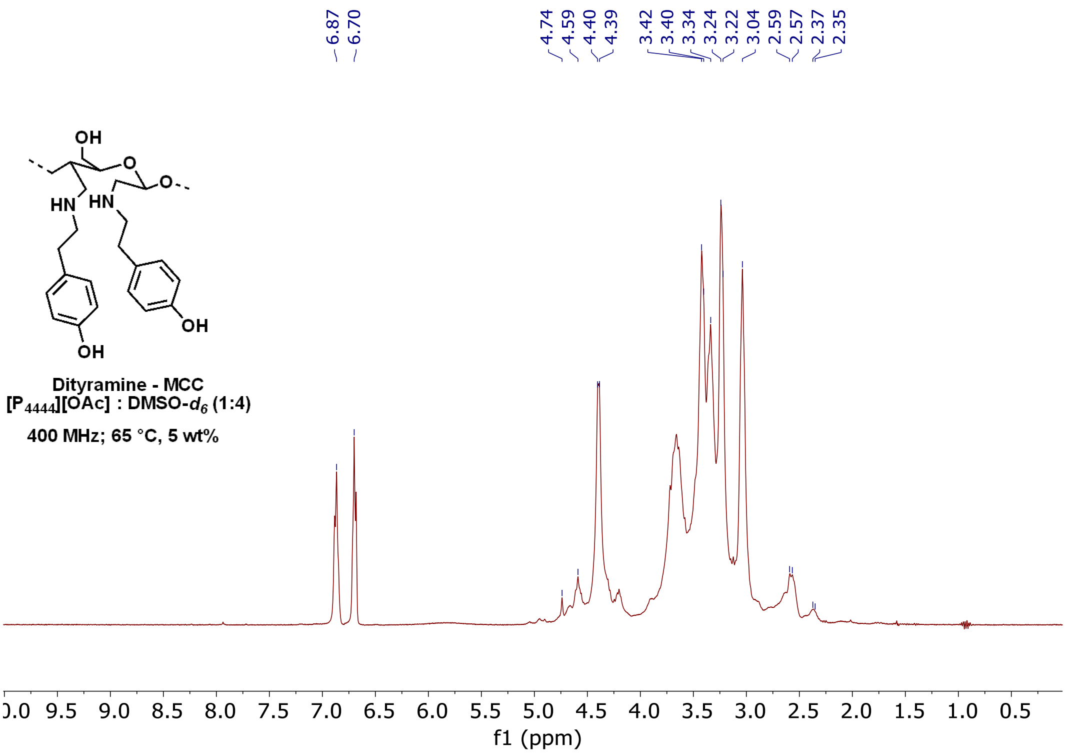

Figure S20. Diffusion edited ^{1}H NMR spectrum ([P_{4444}][OAc] : DMSO-d_6 (v/v = 1 : 4); 400 MHz; 65°C) of dityramine cellulose obtained from MCC-DAC (DO = 8 %; 5 wt%). Dityramine resonances of the aromatic moieties (6.9 – 6.7 ppm) and the ethylene bridge (2.3 – 2.7 ppm) visible. Characteristic diamine cellulose peaks for acetal C1-H at 4.74 and 4.59 ppm.

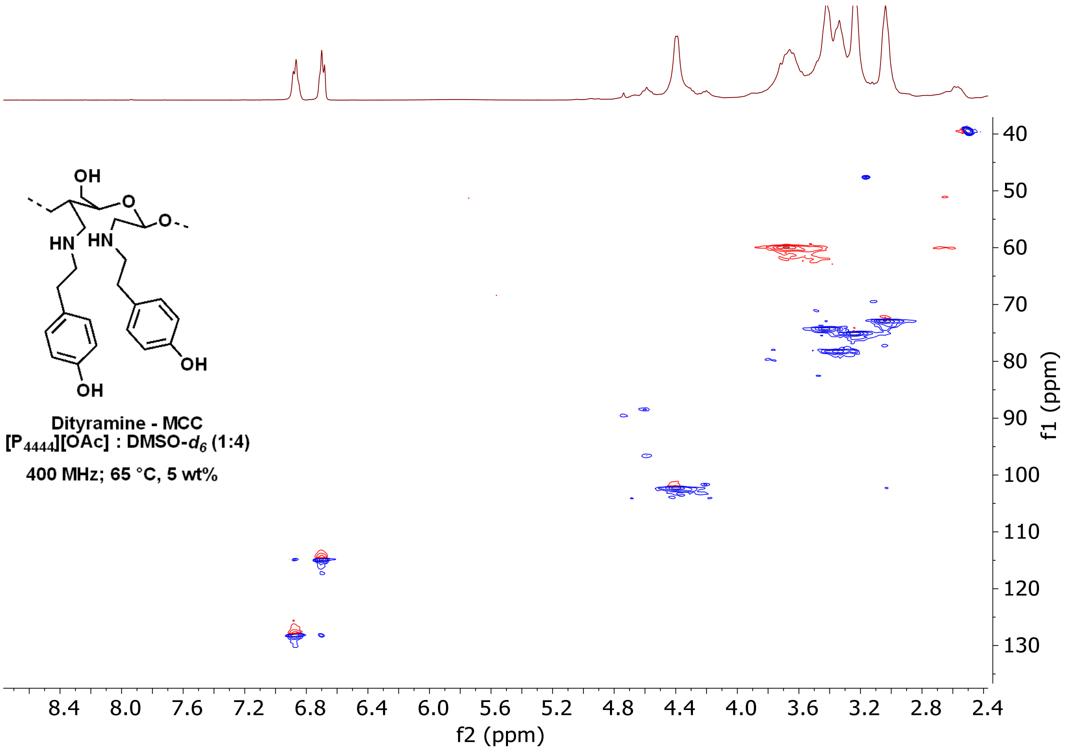

Figure S21. Multiplicity-edited HSQC spectrum ([P_{4444}][OAc]:DMSO-d_6 (v/v = 1 : 4); 400 MHz ^{1}H frequency; 65°C) of dityramine cellulose obtained from MCC-DAC (DO = 8 %; 5 wt%). CH_2 resonances are shown in red, CH / CH_3 signals are shown in blue. On top the diffusion edited ^{1}H spectrum is inserted. Zoom into the polysaccharide region is shown.

3.5) Diethanolamine cellulose from MCC – DAC (DO = 8 %)

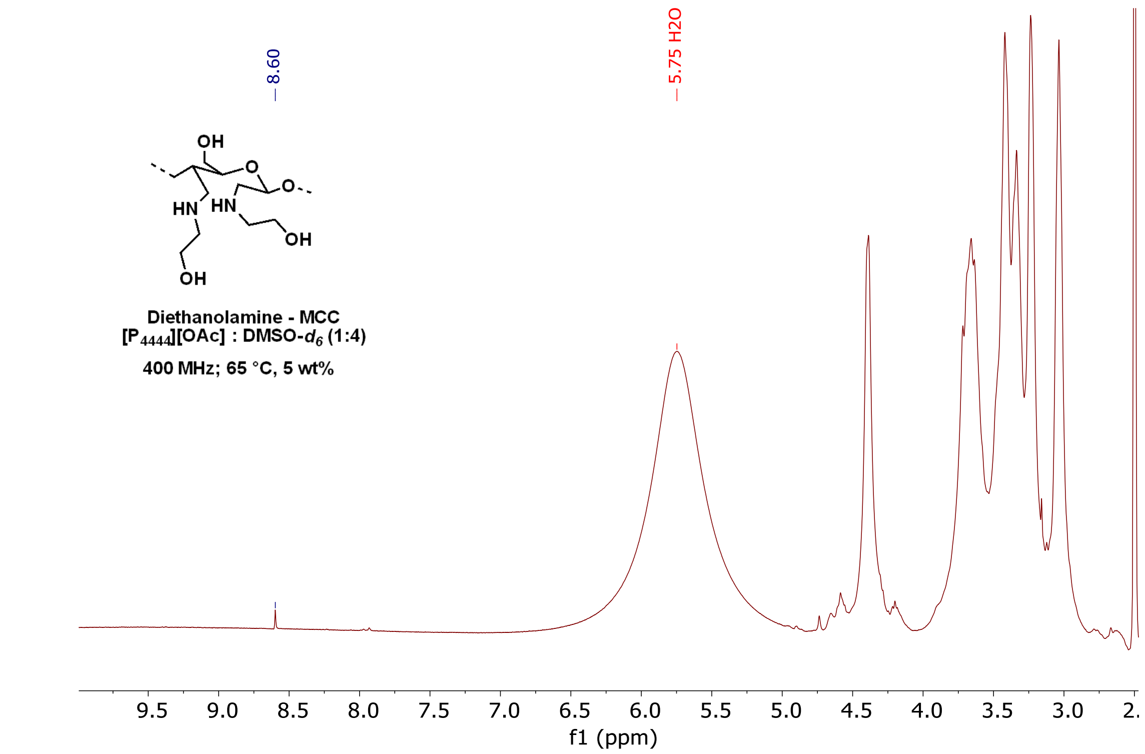

Figure S22. Quantitative ^{1}H NMR spectrum ([P_{4444}][OAc] : DMSO-*d*$_6$ (v/v = 1 : 4); 400 MHz; 65°C) of diethanolamine cellulose obtained from MCC-DAC (DO = 8 %; 5 wt%). Zoom into the polysaccharide and degradation product regions is shown. Peaks of the ethanolamine moieties superimpose with the polysaccharide backbone. Considerable degradation to formiate (8.60 ppm) visible.

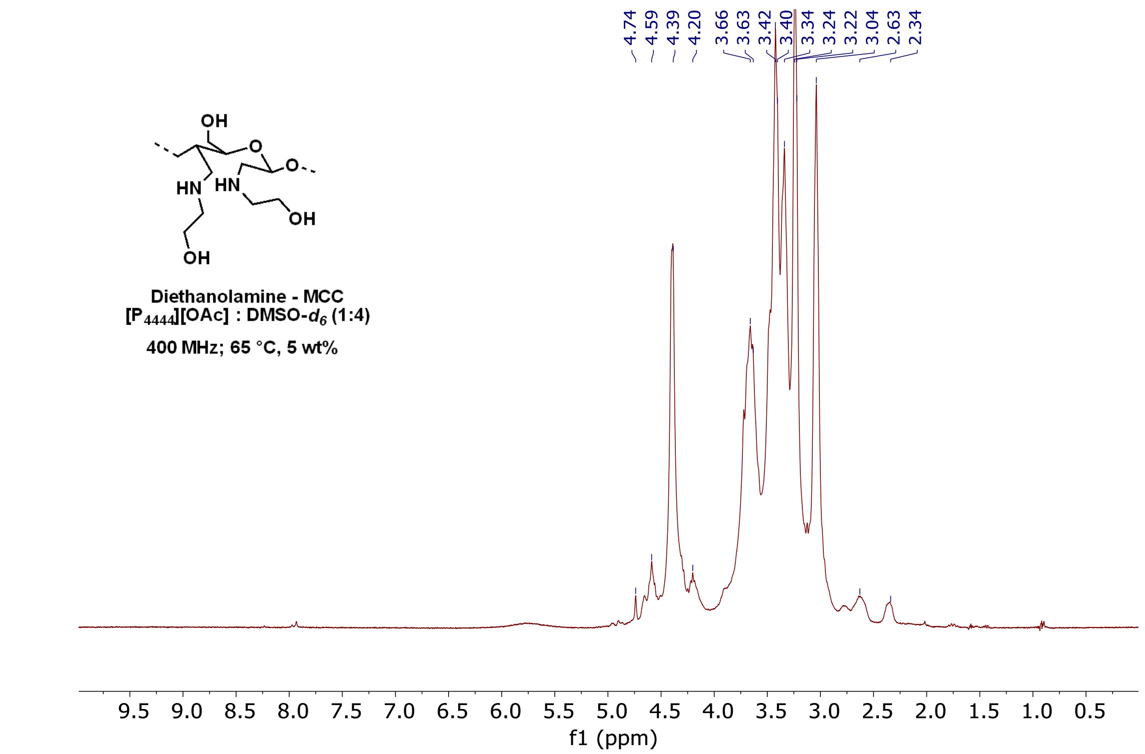

Figure S23. Diffusion edited ^{1}H NMR spectrum ([P_{4444}][OAc] : DMSO-*d*$_6$ (v/v = 1 : 4); 400 MHz; 65°C) of diethanolamine cellulose obtained from MCC-DAC (DO = 8 %; 5 wt%). Diethanolamine resonances of the ethylene bridge (2.3 – 2.7 ppm) visible. Characteristic diamine cellulose peaks for acetal C1-H at 4.74 and 4.59 ppm.

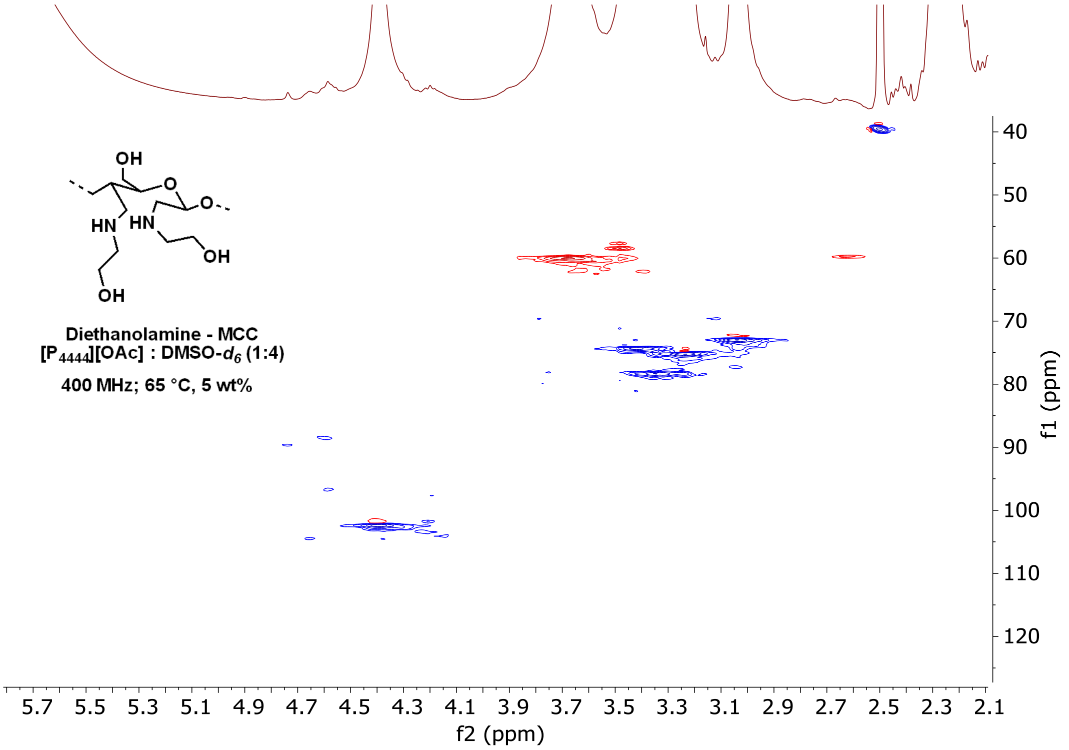

Figure S24. Multiplicity-edited HSQC spectrum ([P_{4444}][OAc]:DMSO-d_6 (v/v = 1 : 4); 400 MHz ^{1}H frequency; 65°C) of diethanolamine cellulose obtained from MCC-DAC (DO = 8 %; 5 wt%). CH_2 resonances are shown in red, CH / CH_3 signals are shown in blue. On top the quantitative ^{1}H spectrum is inserted. Zoom into the polysaccharide region is shown.

3.6) Dibutylamine cellulose from MCC – DAC (DO = 8 %)

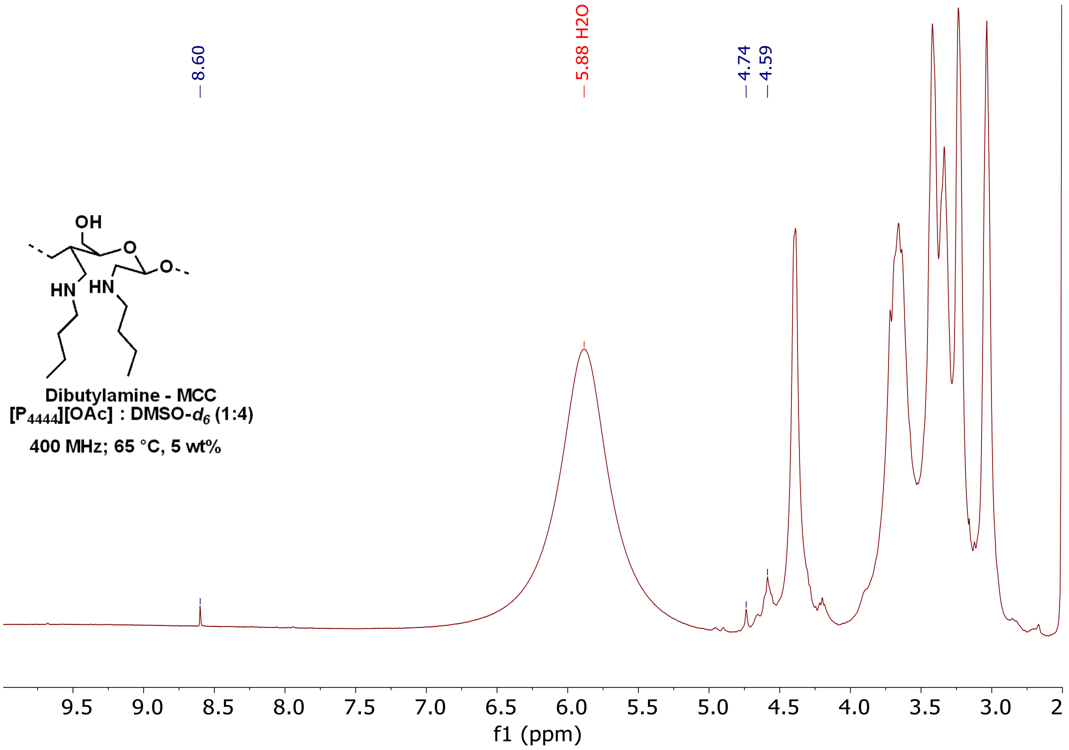

Figure S25. Quantitative ^{1}H NMR spectrum ([P_{4444}][OAc] : DMSO-d_6 (v/v = 1 : 4); 400 MHz; 65°C) of dibutylamine cellulose obtained from MCC-DAC (DO = 8 %; 5 wt%). Zoom into the polysaccharide and degradation product regions is shown. Peaks of the butylamine moieties superimpose with the electrolyte resonances. Considerable degradation to formiate (8.60 ppm) visible.

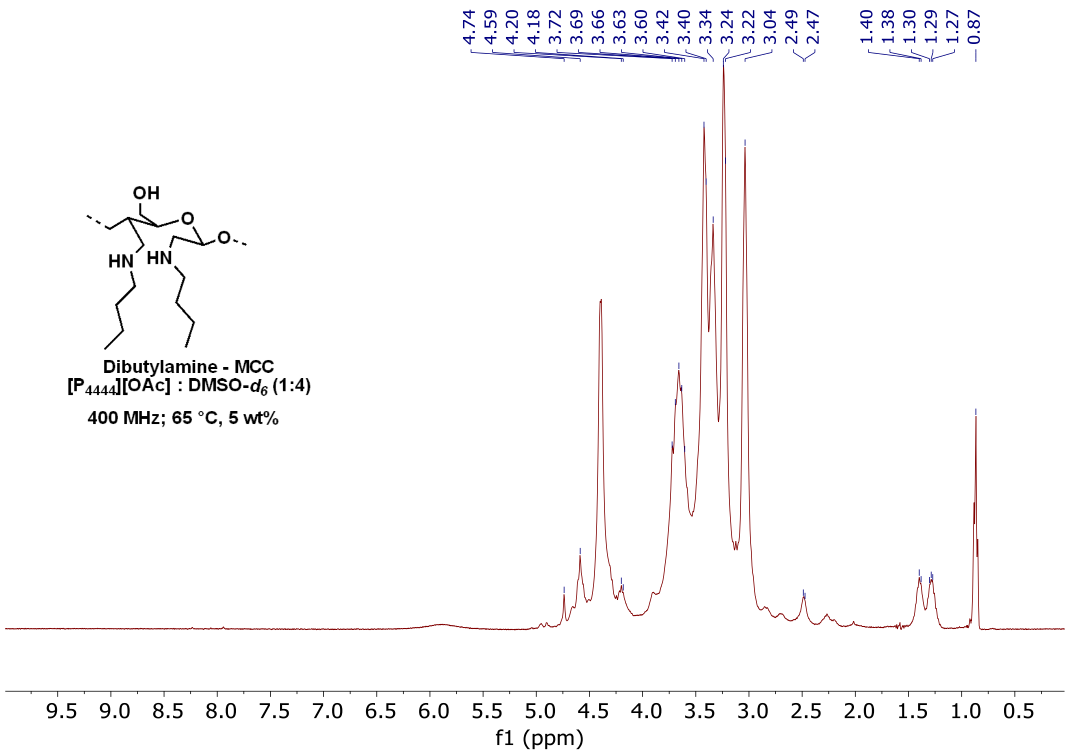

Figure S26. Diffusion edited ^{1}H NMR spectrum ([P_{4444}][OAc] : DMSO-d_6 (v/v = 1 : 4); 400 MHz; 65°C) of dibutylamine cellulose obtained from MCC-DAC (DO = 8 %; 5 wt%). Aliphatic dibutylamine resonances of the alkyl residues (0.8 – 2.5 ppm) clearly visible. Characteristic diamine cellulose peaks for acetal C1-H at 4.74 and 4.59 ppm.

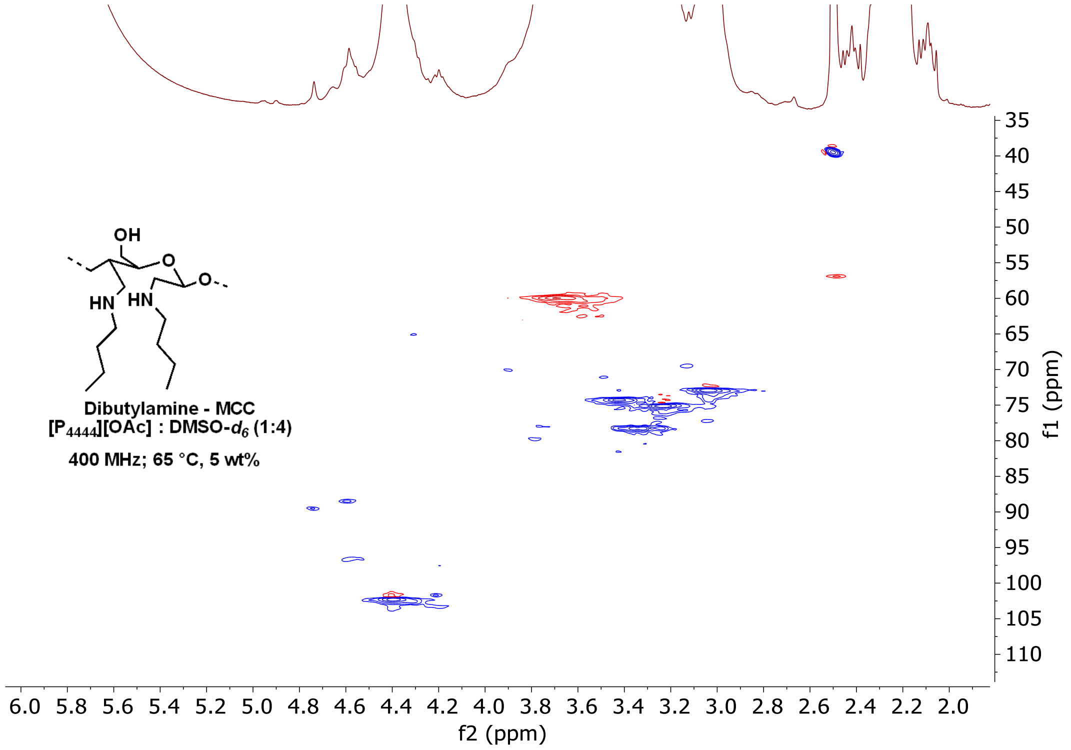

Figure S27. Multiplicity-edited HSQC spectrum ([P_{4444}][OAc]:DMSO-d_6 (v/v = 1 : 4); 400 MHz ^{1}H frequency; 65°C) of dibutylamine cellulose obtained from MCC-DAC (DO = 8 %; 5 wt%). CH_2 resonances are shown in red, CH / CH_3 signals are shown in blue. On top the quantitative ^{1}H spectrum is inserted. Zoom into the polysaccharide region is shown.

3.7) Dihexylamine cellulose from MCC – DAC (DO = 8 %)

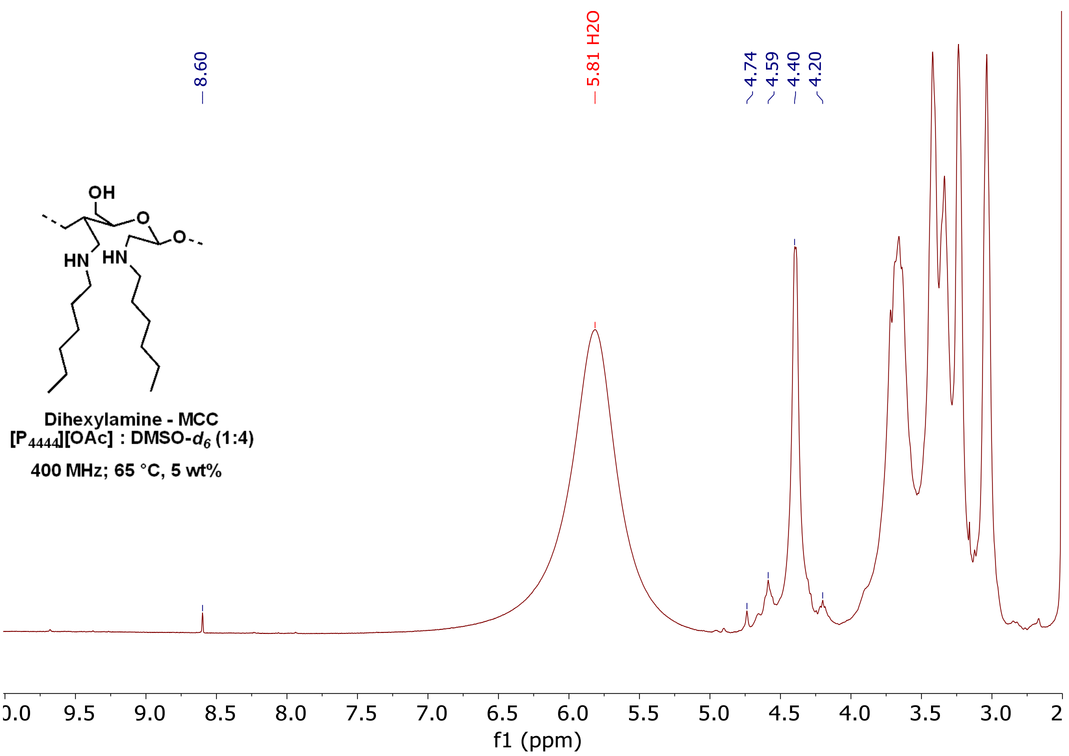

Figure S28. Quantitative ^{1}H NMR spectrum ([P_{4444}][OAc] : DMSO-d_6 (v/v = 1 : 4); 400 MHz; 65°C) of dihexylamine cellulose obtained from MCC-DAC (DO = 8 %; 5 wt%). Zoom into the polysaccharide and degradation product regions is shown. Peaks of the butylamine moieties superimpose with the electrolyte resonances. Considerable degradation to formiate (8.60 ppm) visible.

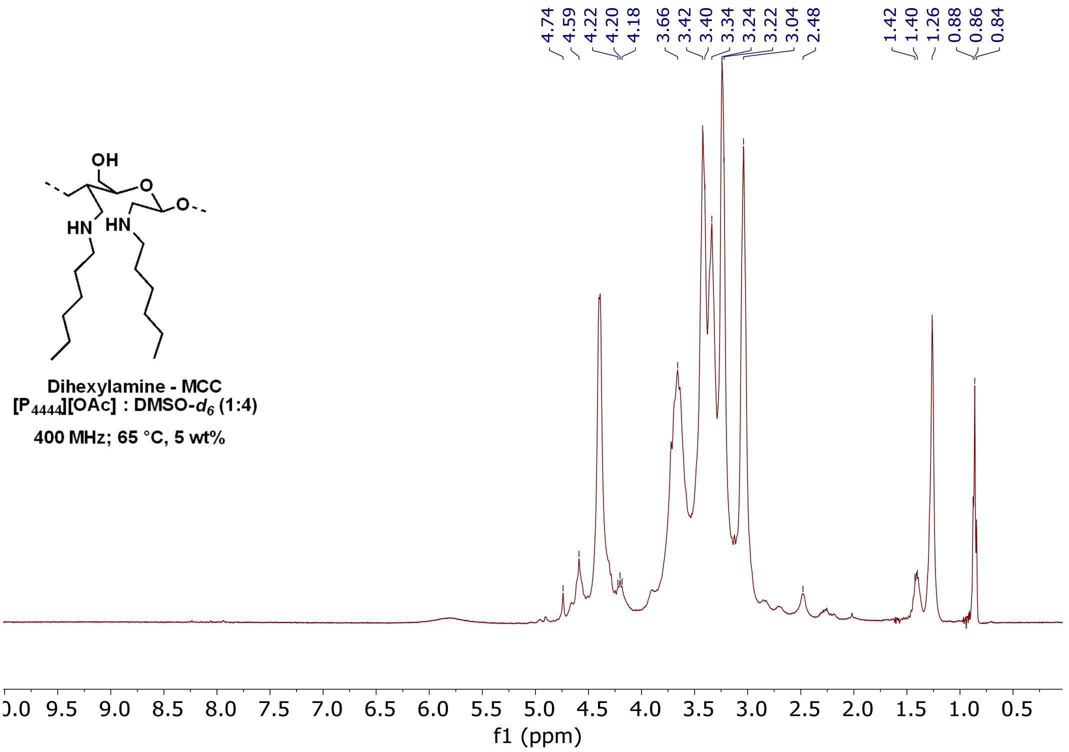

Figure S29. Diffusion edited ^{1}H NMR spectrum ([P_{4444}][OAc] : DMSO-d_6 (v/v = 1 : 4); 400 MHz; 65°C) of dihexylamine cellulose obtained from MCC-DAC (DO = 8 %; 5 wt%). Aliphatic dihexylamine resonances of the alkyl residues (0.8 – 2.5 ppm) clearly visible. Characteristic diamine cellulose peaks for acetal C1-H at 4.74 and 4.59 ppm.

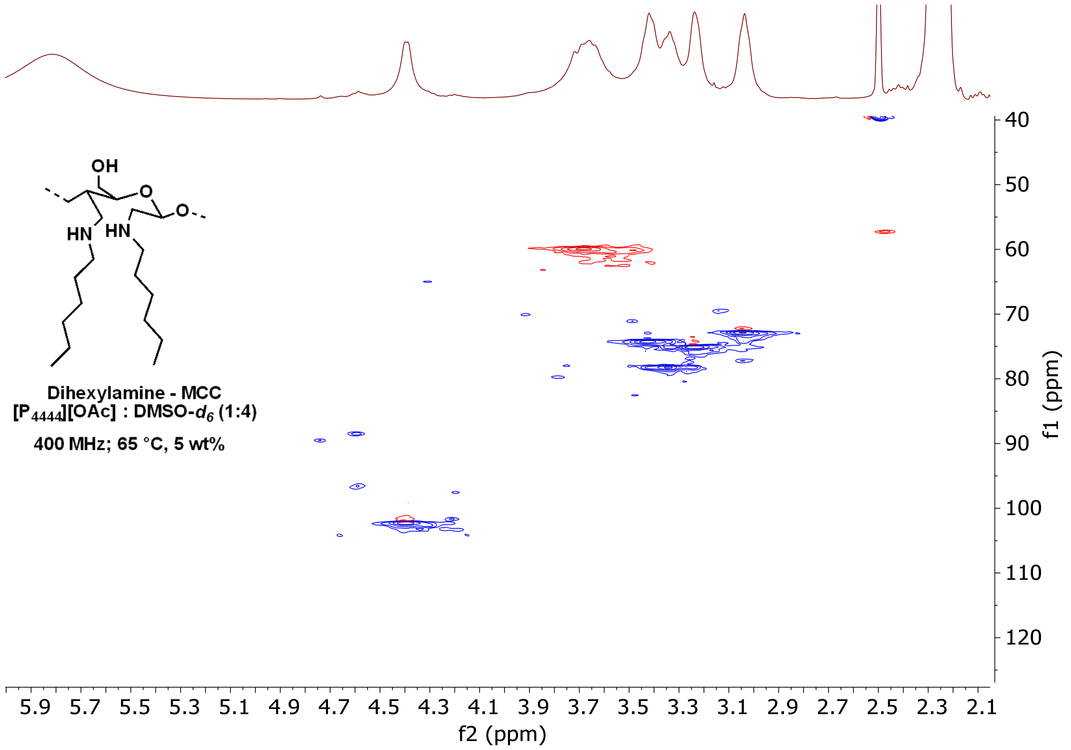

Figure S30. Multiplicity-edited HSQC spectrum ([P_{4444}][OAc]:DMSO-d_6 (v/v = 1 : 4); 400 MHz ^{1}H frequency; 65°C) of dihexylamine cellulose obtained from MCC-DAC (DO = 8 %; 5 wt%). CH_2 resonances are shown in red, CH / CH_3 signals are shown in blue. On top the quantitative ^{1}H spectrum is inserted. Zoom into the polysaccharide region is shown.

3.8) Dianilineamine cellulose from MCC – DAC (DO = 8 %)

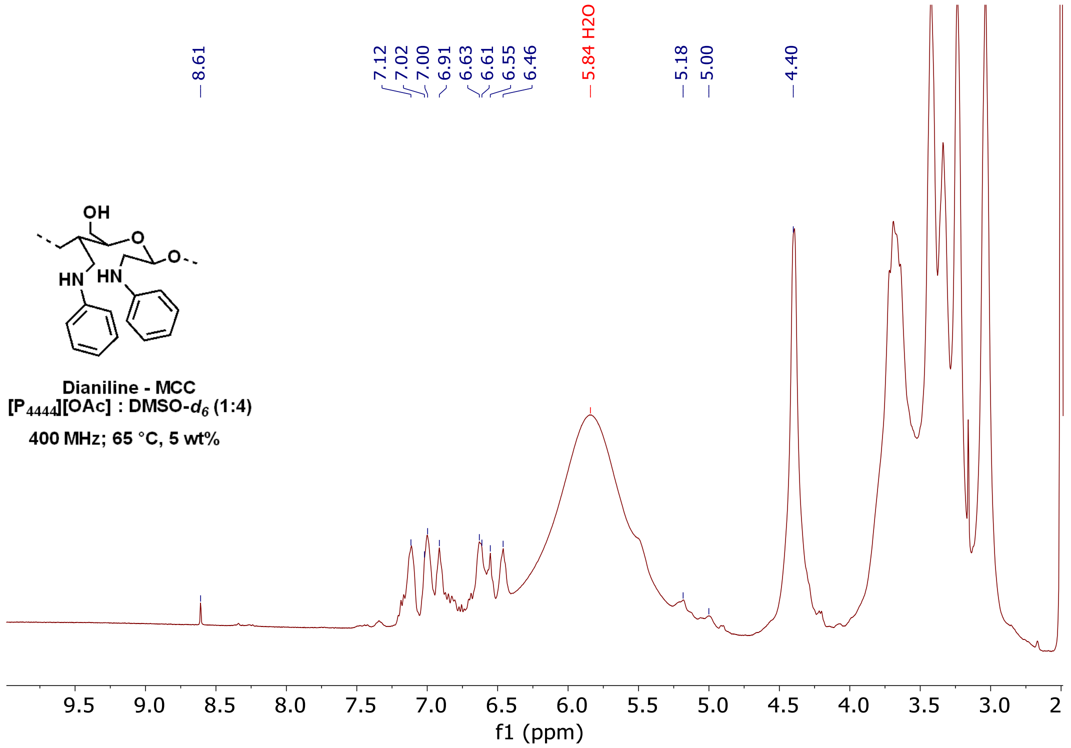

Figure S31. Quantitative ^{1}H NMR spectrum ([P_{4444}][OAc] : DMSO-d_6 (v/v = 1 : 4); 400 MHz; 65°C) of dianiline cellulose obtained from MCC-DAC (DO = 8 %; 5 wt%). Zoom into the polysaccharide and degradation product regions is shown. Resonances of newly introduced aromatic moieties and considerable degradation to formiate (8.60 ppm) visible.

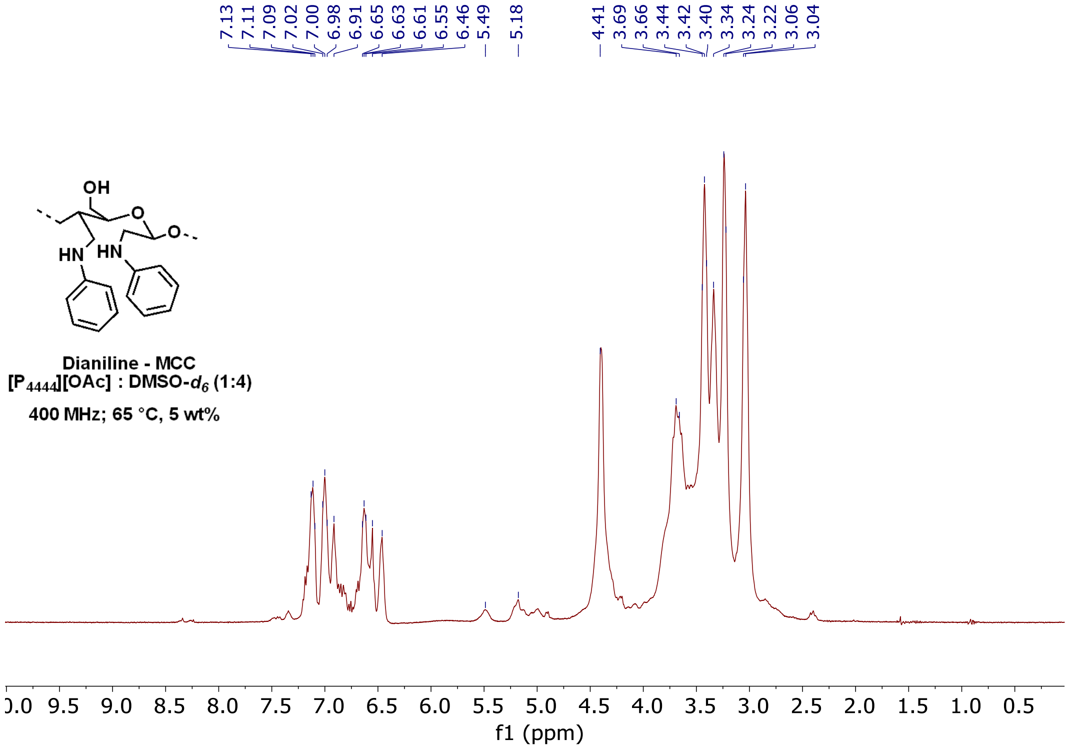

Figure S32. Diffusion edited ^{1}H NMR spectrum ([P$_{4444}$][OAc] : DMSO-d_6 (v/v = 1 : 4); 400 MHz; 65°C) of dianiline cellulose obtained from MCC-DAC (DO = 8 %; 5 wt%). Dianiline resonances of the aromatic moieties (6.4 – 7.2 ppm) visible. Characteristic diamine cellulose peaks for acetal C1-H at 5.49 and 5.18 ppm.

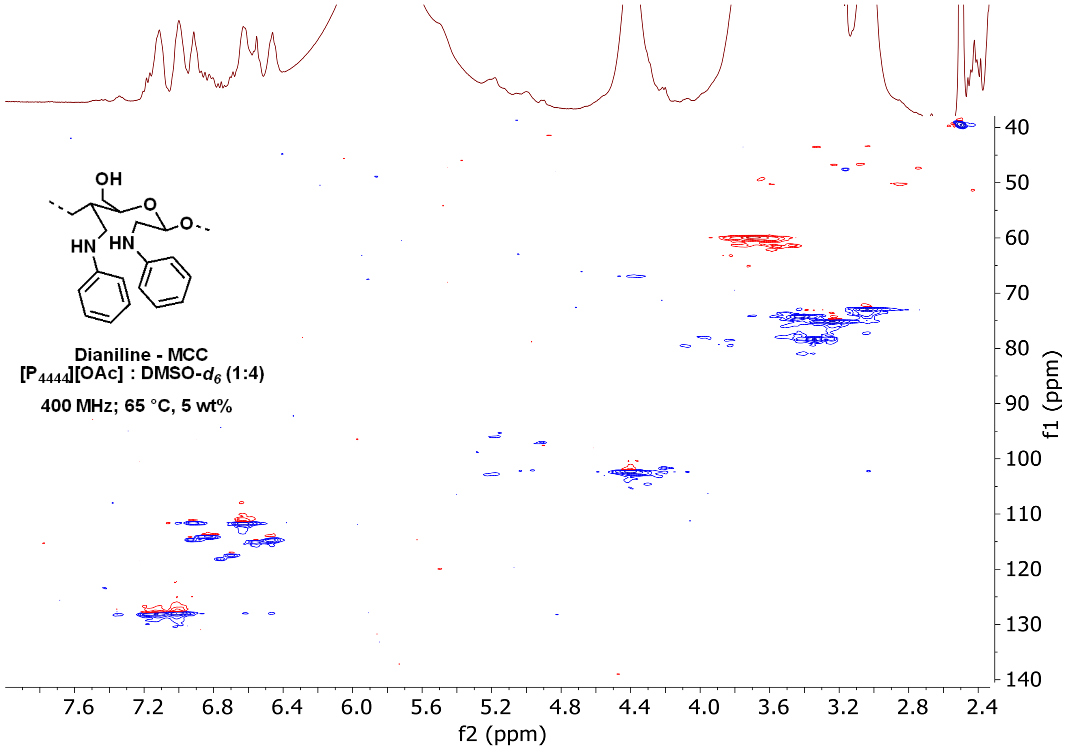

Figure S33. Multiplicity-edited HSQC spectrum ([P$_{4444}$][OAc]:DMSO-d_6 (v/v = 1 : 4); 400 MHz ^{1}H frequency; 65°C) of dianiline cellulose obtained from MCC-DAC (DO = 8 %; 5 wt%). CH_2 resonances are shown in red, CH / CH_3 signals are shown in blue. On top the quantitative ^{1}H spectrum is inserted. Zoom into the polysaccharide region is shown.

3.9) Softwood kraft pulp (SKP) starting material

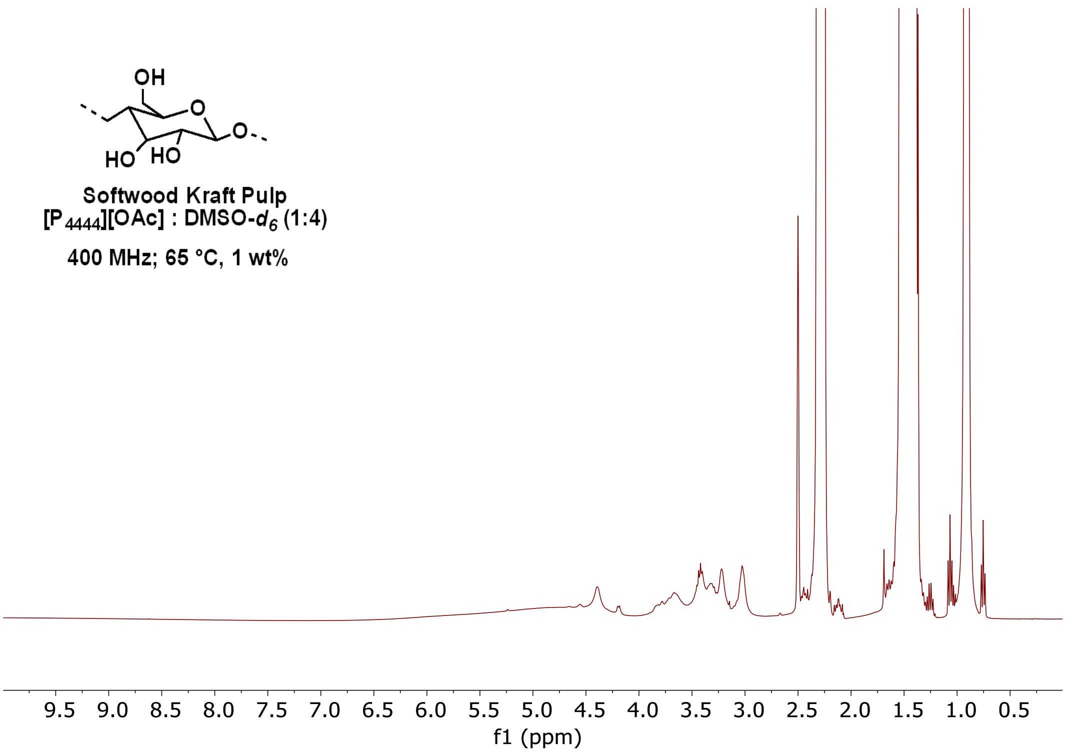

Figure S34. Quantitative ^{1}H NMR spectrum ([P_{4444}][OAc] : DMSO-d_6 (v/v = 1 : 4); 400 MHz; 65°C) of the softwood kraft pulp starting material (1 wt%). Full spectral area is shown. Owing to the high molecular weight of the material, a lower concentration of 1 wt% had to be used to allow dissolution. This resulted in a significant decrease in resolution.

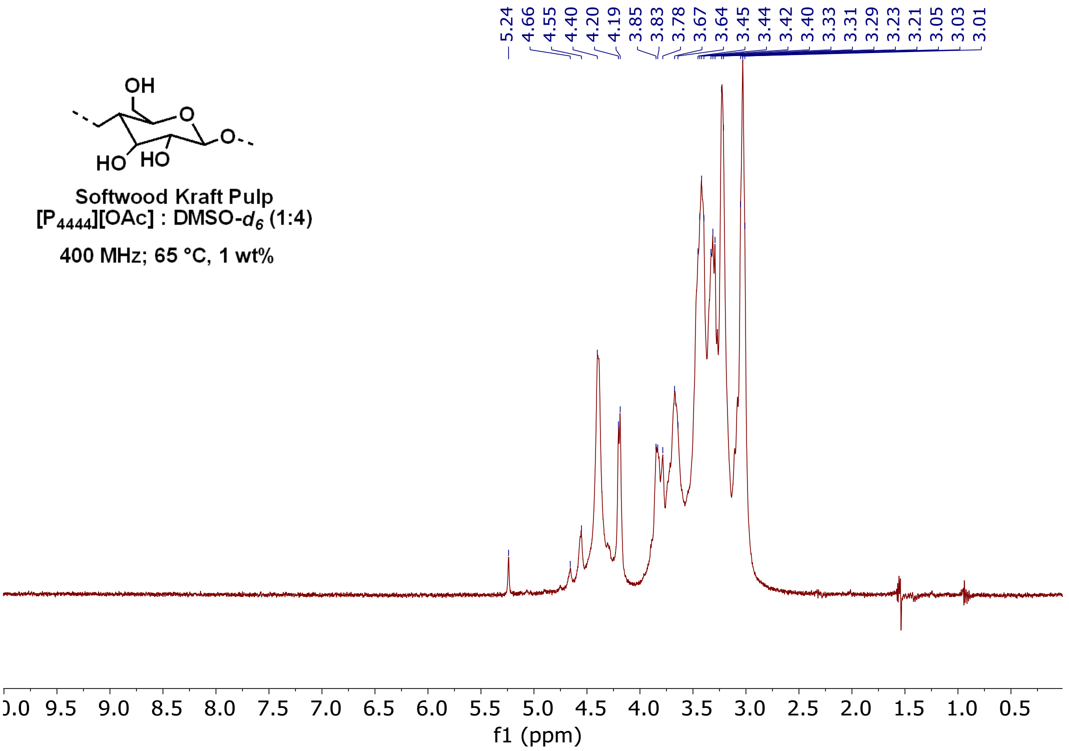

Figure S35. Diffusion edited ^{1}H NMR spectrum ([P_{4444}][OAc] : DMSO-d_6 (v/v = 1 : 4); 400 MHz; 65°C) of the softwood kraft pulp starting material (1 wt%). Full spectral area is shown. Besides the cellulose resonances the pulp exhibited a significant hemicellulose content, as evidenced by different polysaccharide C1-H signals at 5.24, 4.66, 4.55 and 4.20 ppm.

3.10) Dialdehyde cellulose (SKP, DO = 39 %)

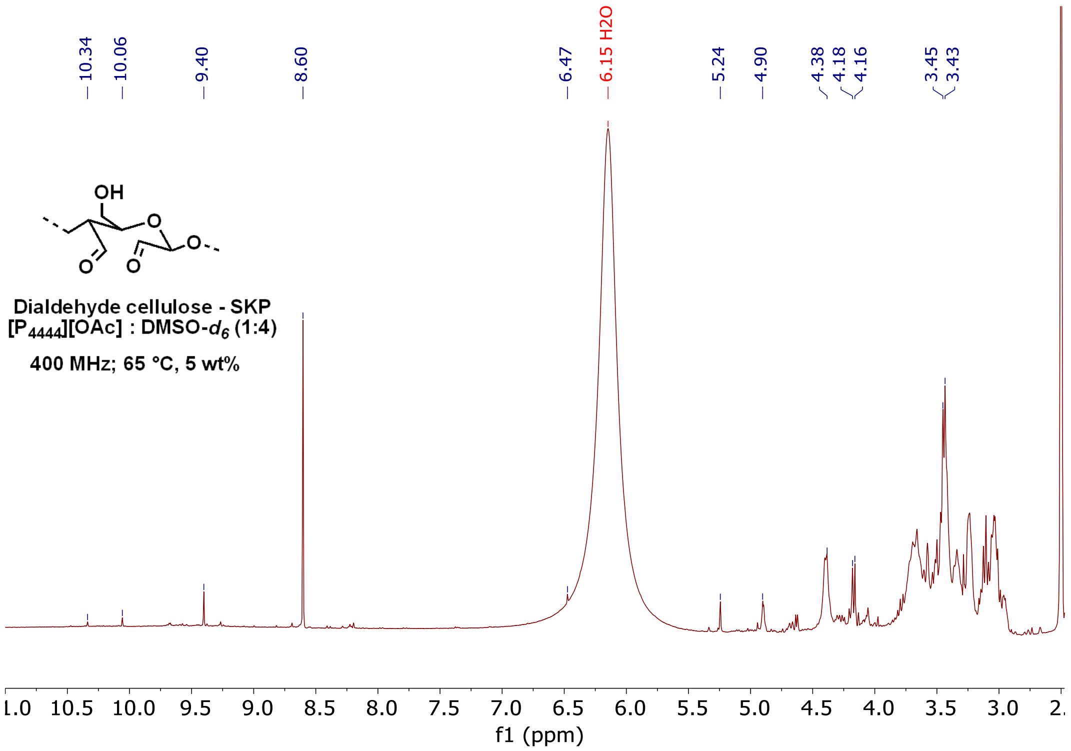

Figure S36. Quantitative ^{1}H NMR spectrum ([P_{4444}][OAc] : DMSO-d_6 (v/v = 1 : 4); 400 MHz; 65°C) of softwood kraft pulp - dialdehyde cellulose (DO = 39 %; 5 wt%). Zoom into the polysaccharide and degradation product regions is shown. Strong degradation to low molecular weight compounds is observed. Major degradation product is formiate ($HCOO^{-}$), exemplified by the strong peak at 8.60 ppm.

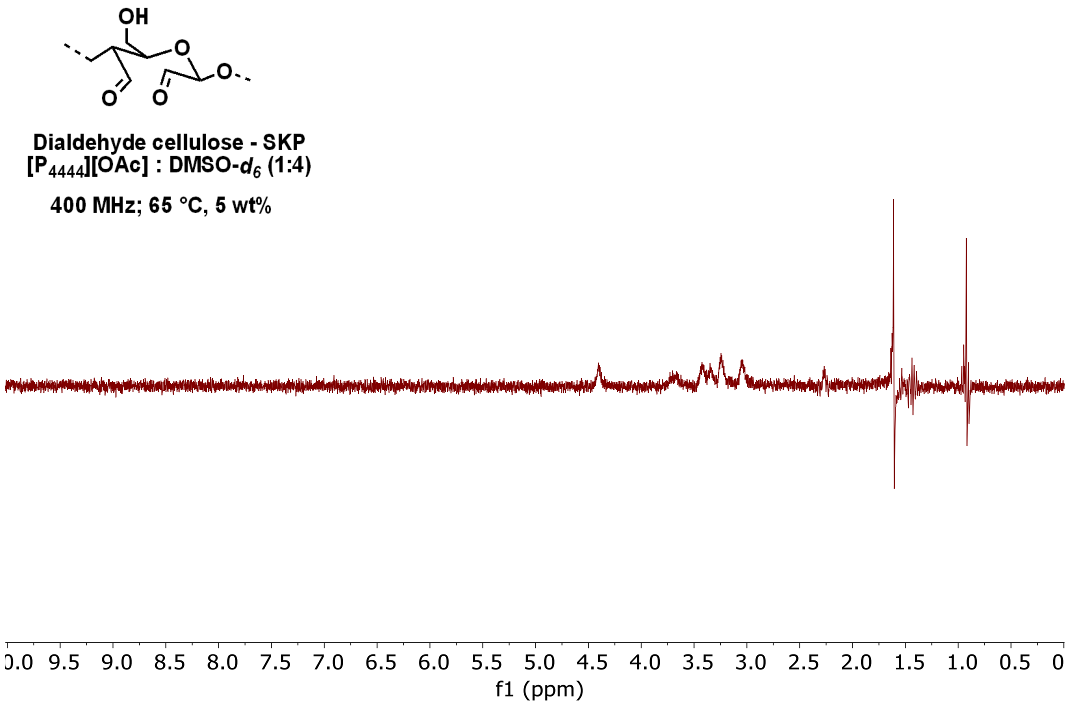

Figure S37. Diffusion edited ^{1}H NMR spectrum ([P_{4444}][OAc] : DMSO-d_6 (v/v = 1 : 4); 400 MHz; 65°C) of of softwood kraft pulp - dialdehyde cellulose (DO = 39 %; 5 wt%). Full spectral area is shown. The spectrum shows no significant peaks, suggesting that all the polymeric constituents were completely degraded.

3.11) Diethanolamine cellulose from SKP – DAC (DO = 39 %)

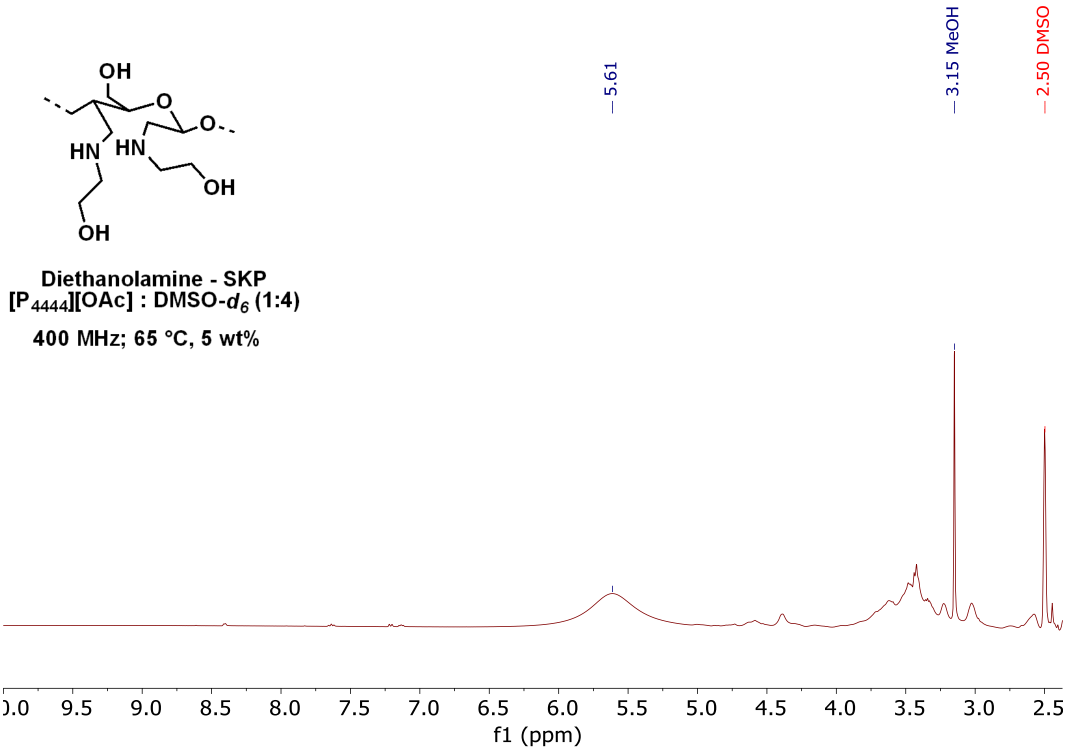

Figure S38. Quantitative ^{1}H NMR spectrum ([P_{4444}][OAc] : DMSO-*d*$_6$ (v/v = 1 : 4); 400 MHz; 65°C) of diethanolamine cellulose obtained from SKP-DAC (DO = 39 %; 5 wt%). Zoom into the polysaccharide and degradation product regions is shown. Owing to strong peak superposition assignment of the resonances is difficult. Only minor degradation to formiate (8.60 ppm) visible. Considerable methanol impurity from insufficient drying.

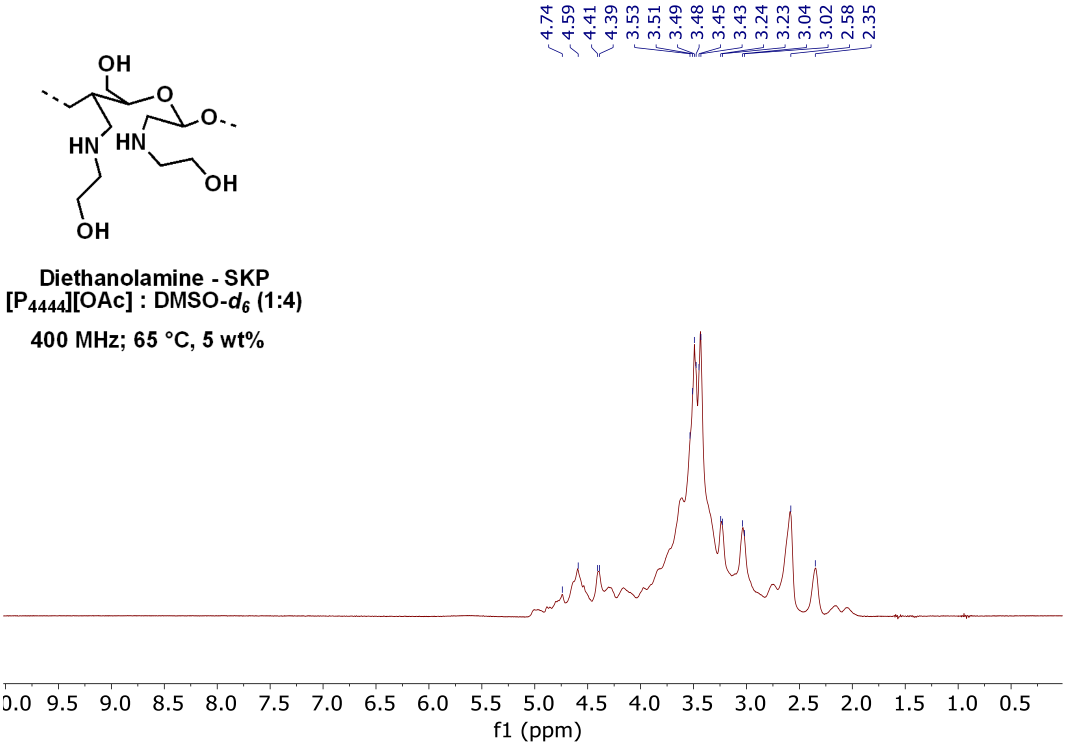

Figure S39. Diffusion edited ^{1}H NMR spectrum ([P_{4444}][OAc] : DMSO-*d*$_6$ (v/v = 1 : 4); 400 MHz; 65°C) of diethanolamine cellulose obtained from SKP-DAC (DO = 39 %; 5 wt%). Strong peak superposition in the polysaccharide region. Diethanolamine resonances of the ethylene bridge (2.3 – 2.7 ppm) visible. Characteristic diamine cellulose peaks for acetal C1-H at 4.74 and 4.59 ppm.

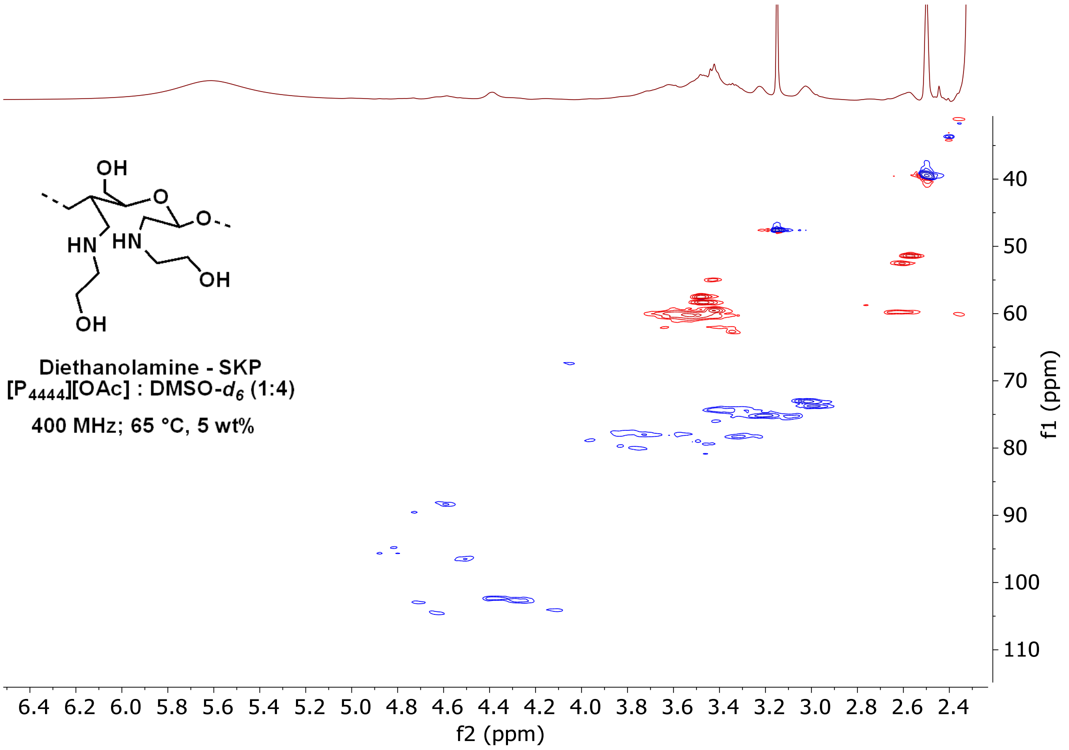

Figure S40. Multiplicity-edited HSQC spectrum ([P_{4444}][OAc]:DMSO-d_6 (v/v = 1 : 4); 400 MHz ^{1}H frequency; 65°C) of diethanolamine cellulose obtained from SKP-DAC (DO = 39 %; 5 wt%). CH_2 resonances are shown in red, CH / CH_3 signals are shown in blue. On top the quantitative ^{1}H spectrum is inserted. Zoom into the polysaccharide region is shown.

3.12) Dianilineamine cellulose from SKP – DAC (DO = 39 %)

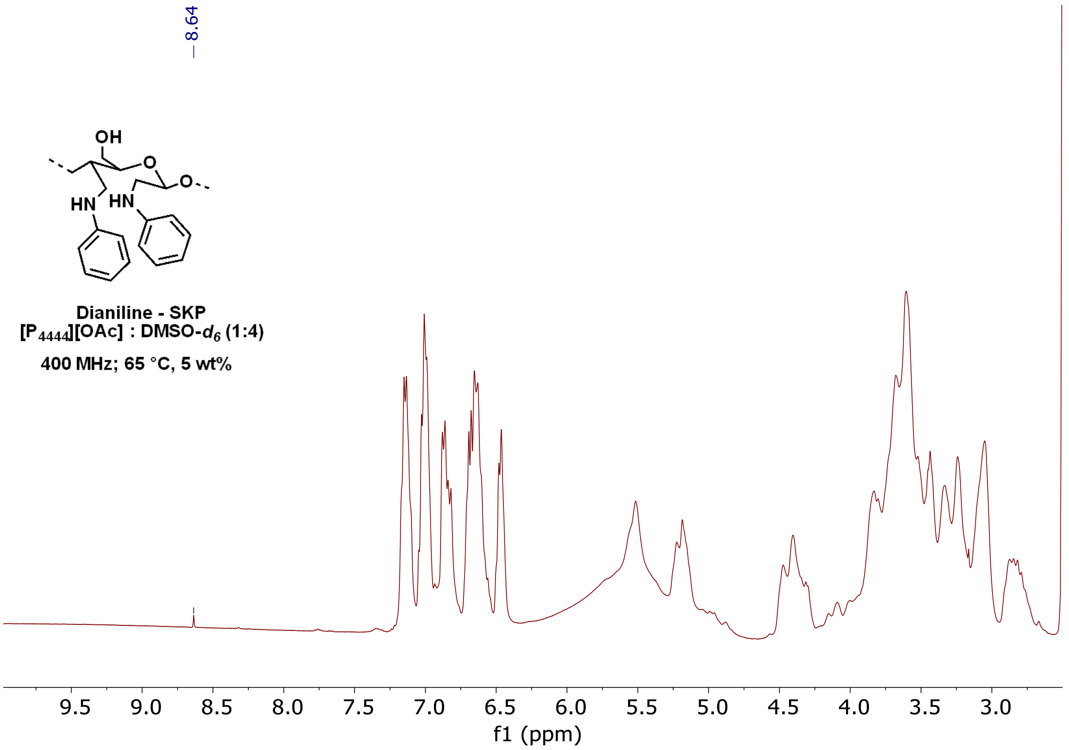

Figure S41. Quantitative ^{1}H NMR spectrum ([P_{4444}][OAc] : DMSO-d_6 (v/v = 1 : 4); 400 MHz; 65°C) of dianiline cellulose obtained from SKP-DAC (DO = 39 %; 5 wt%). Zoom into the polysaccharide and degradation product regions is shown. Zoom into the polysaccharide and degradation product regions is shown. Resonances of newly introduced aromatic moieties and slight degradation to formiate (8.64 ppm) visible.

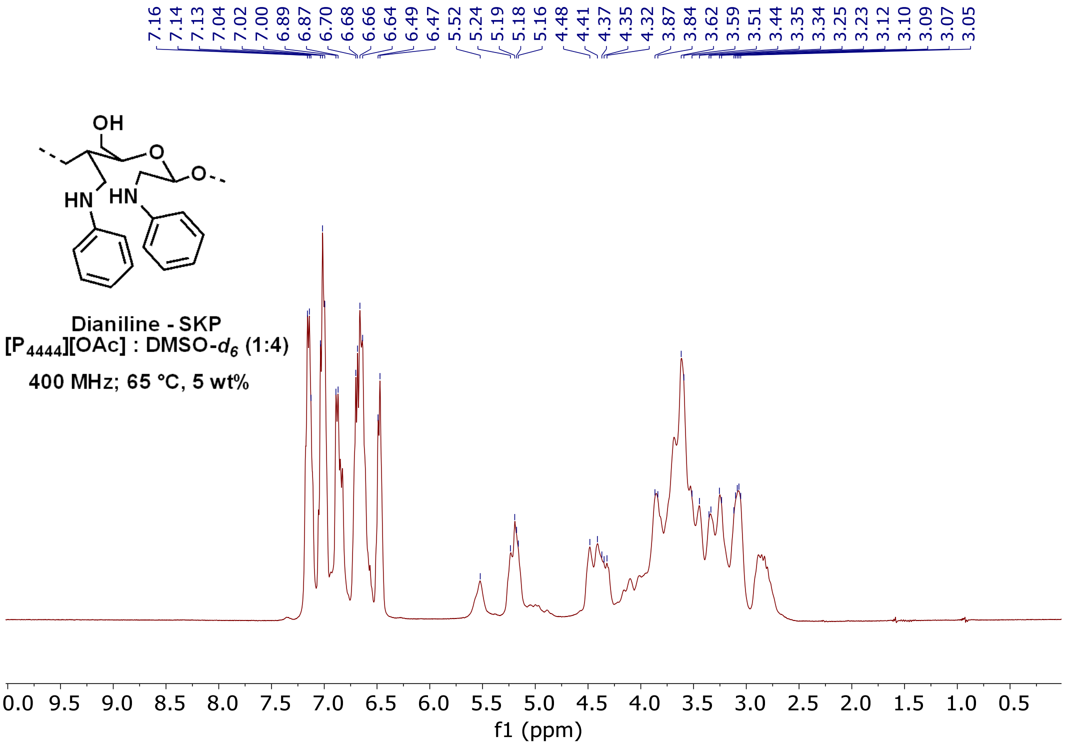

Figure S42. Diffusion edited ^{1}H NMR spectrum ($[P_{4444}][OAc]$: DMSO-d_6 (v/v = 1 : 4); 400 MHz; 65°C) of dianiline cellulose obtained from SKP-DAC (DO = 39 %; 5 wt%). Dianiline resonances of the aromatic moieties (6.4 – 7.2 ppm) visible. Characteristic diamine cellulose peaks for acetal C1-H at 5.52 and 5.19 ppm.

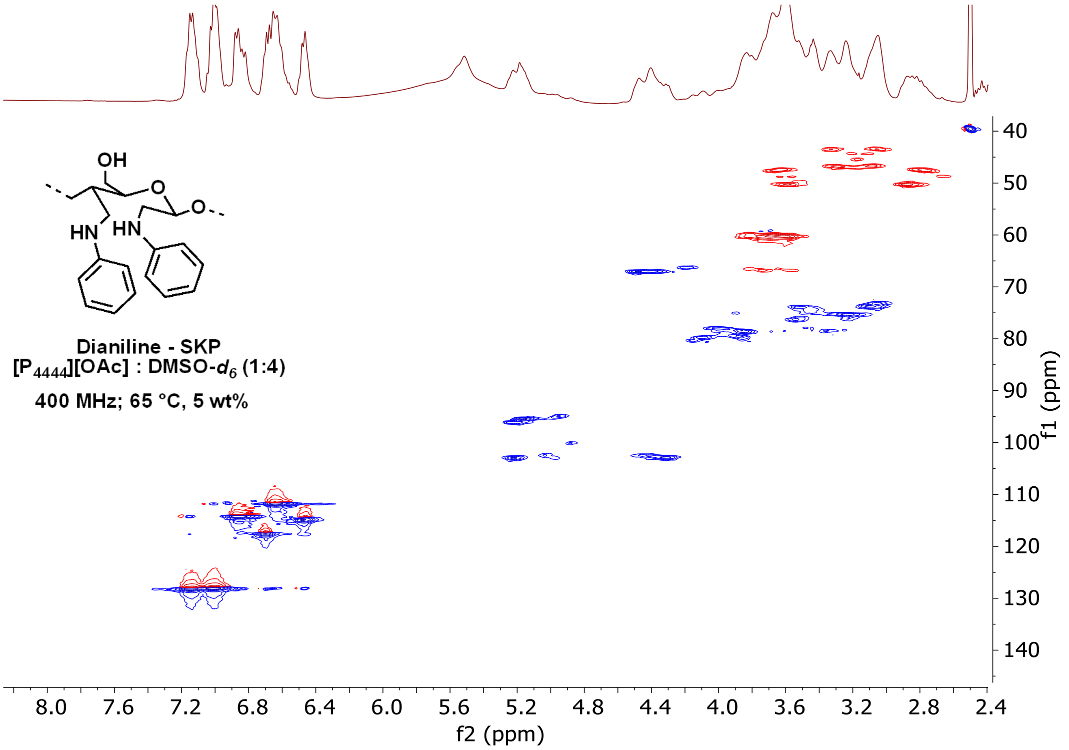

Figure S43. Multiplicity-edited HSQC spectrum ($[P_{4444}][OAc]$:DMSO-d_6 (v/v = 1 : 4); 400 MHz ^{1}H frequency; 65°C) of dianiline cellulose obtained from SKP-DAC (DO = 39 %; 5 wt%). CH_2 resonances are shown in red, CH / CH_3 signals are shown in blue. On top the quantitative ^{1}H spectrum is inserted. Zoom into the polysaccharide region is shown.

4) FTIR spectra for the isolated diamino celluloses

4.1) Diamine celluloses obtained from microcrystalline cellulose (MCC, Avicel® PH-101) (DO = 8 %)

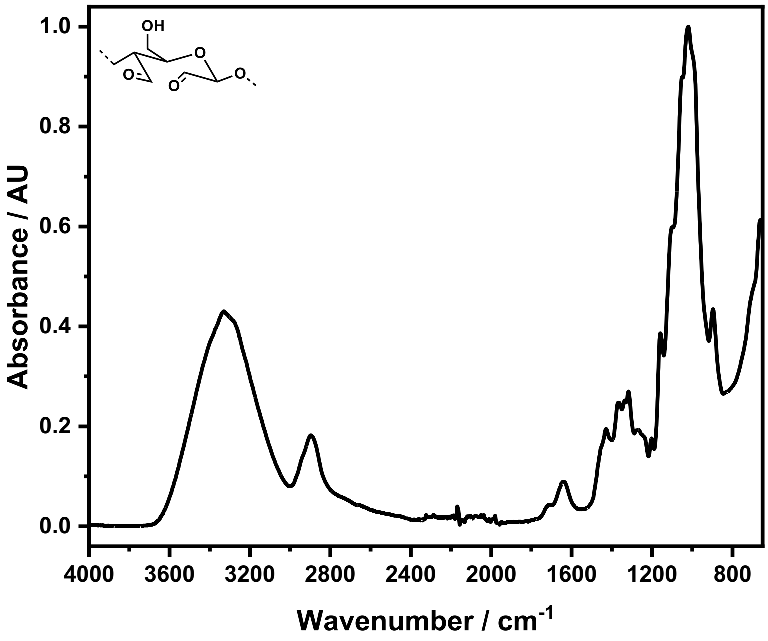

Figure S44. Normalized Fourier-transform infrared spectra of DAC (DO = 8 %) obtained from MCC.

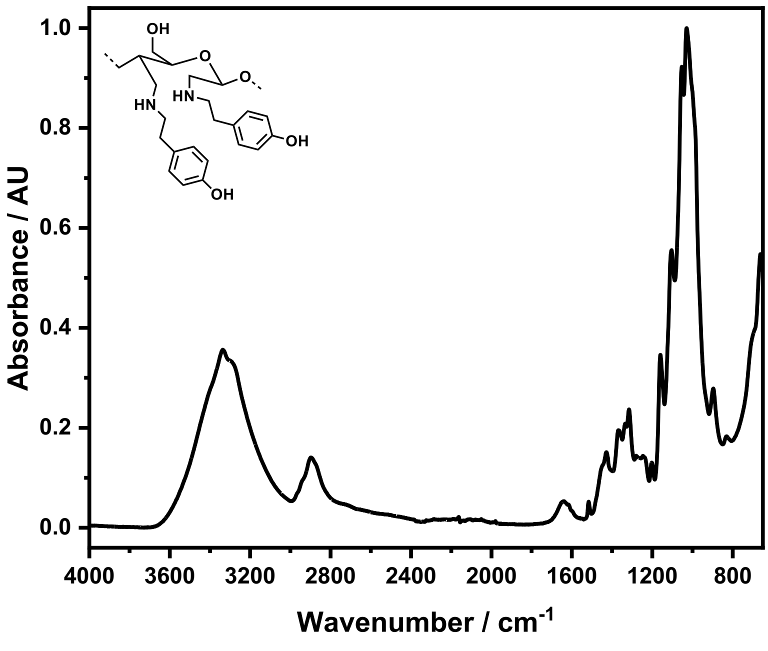

Figure S45. Normalized Fourier-transform infrared spectra of dityramine cellulose obtained from MCC-DAC (DO = 8 %).

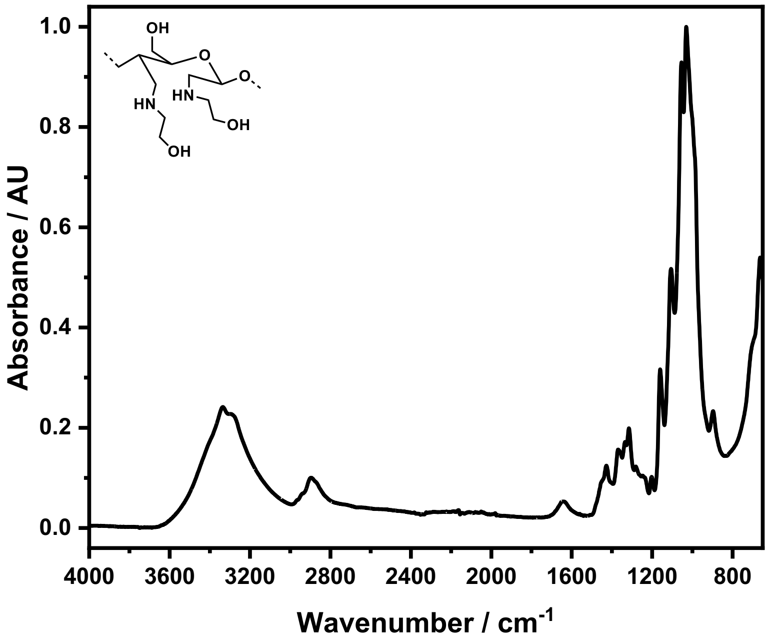

Figure S46. Normalized Fourier-transform infrared spectra of diethanolamine cellulose obtained from MCC-DAC (DO = 8 %).

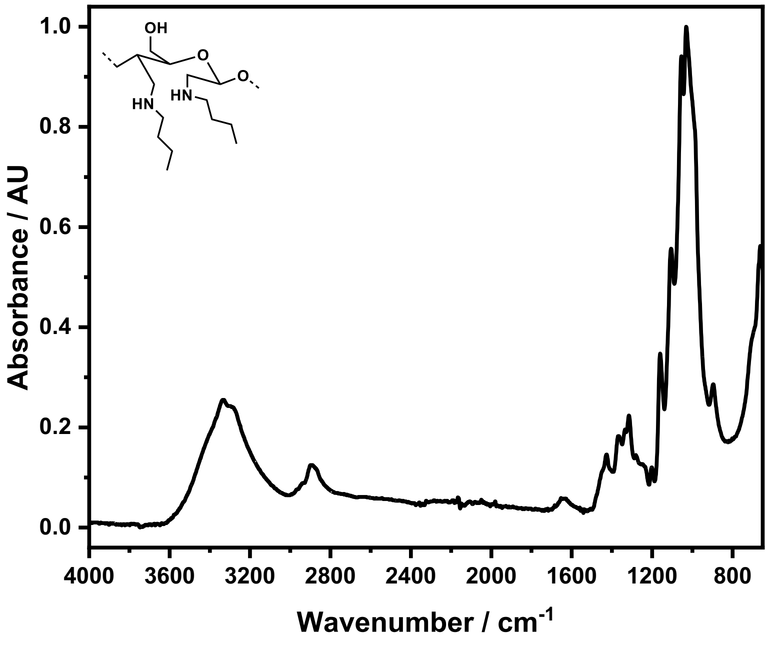

Figure S47. Normalized Fourier-transform infrared spectra of dibutylamine cellulose obtained from MCC-DAC (DO = 8 %).

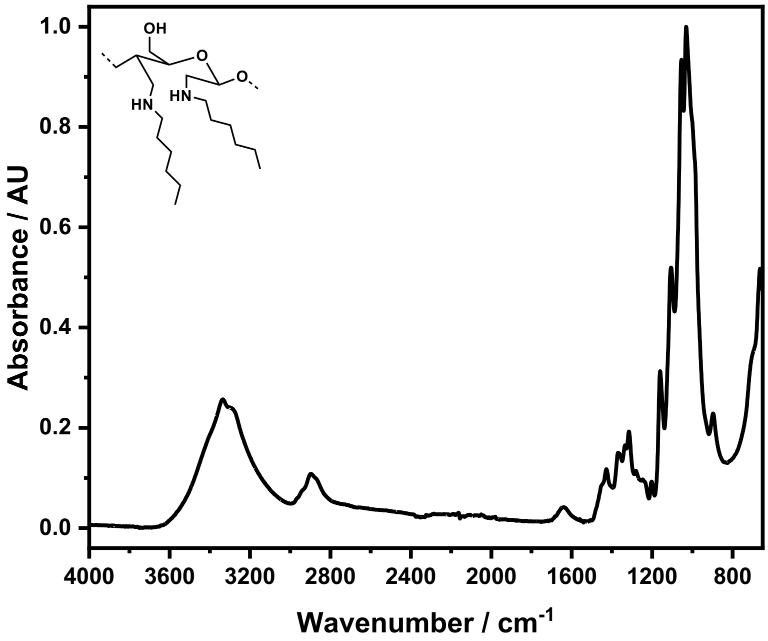

Figure S48. Normalized Fourier-transform infrared spectra of dihexylamine cellulose obtained from MCC-DAC (DO = 8 %).

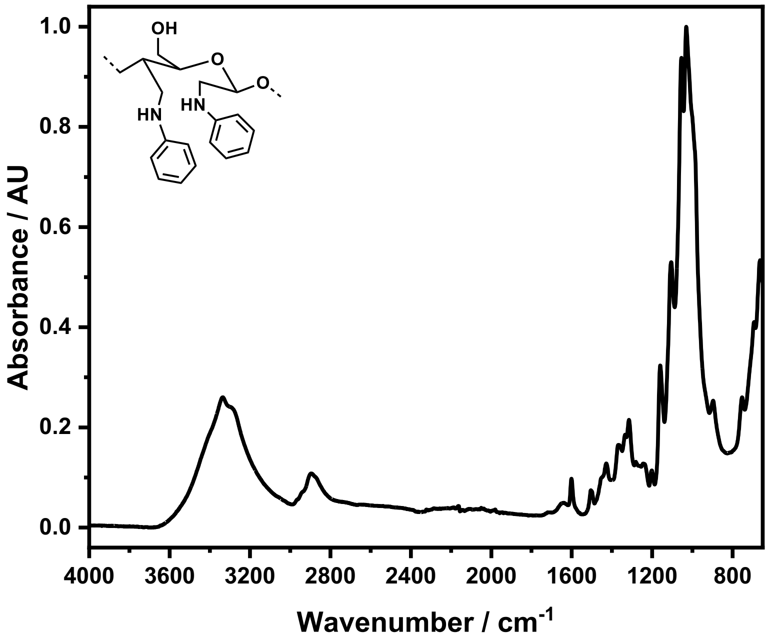

Figure S49. Normalized Fourier-transform infrared spectra of dianiline cellulose obtained from MCC-DAC (DO = 8 %).

4.2) Diamine celluloses obtained from partially oxidized softwood kraft pulp (DO = 39 %)

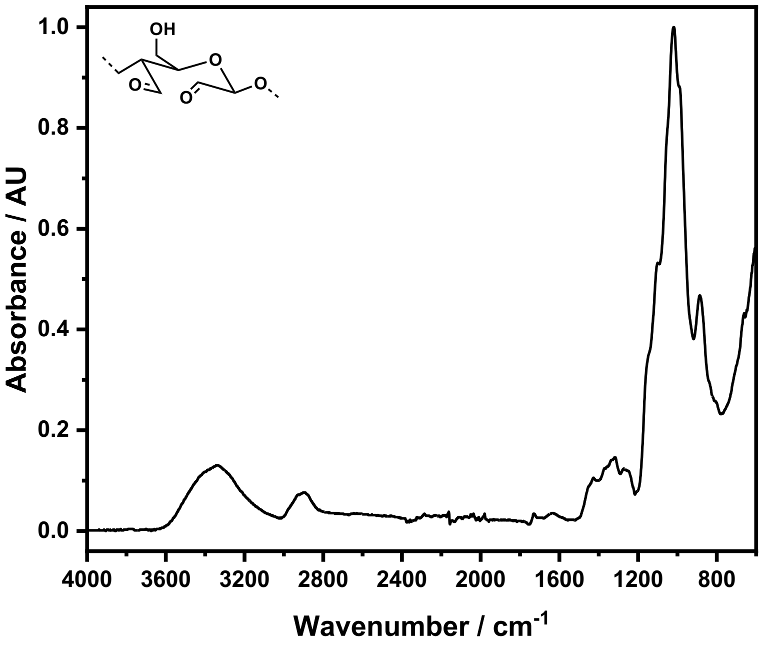

Figure S50. Normalized Fourier-transform infrared spectra of DAC (DO = 39 %) from SKP.

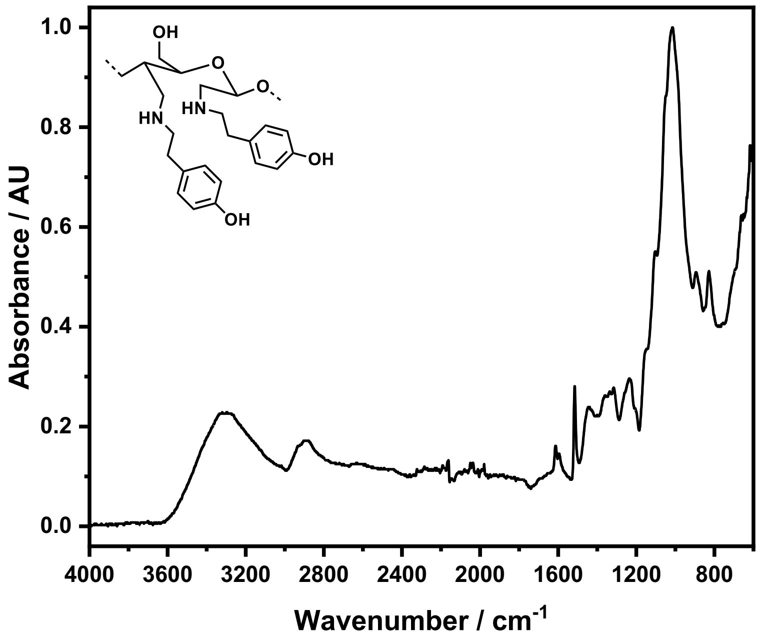

Figure S51. Normalized Fourier-transform infrared spectra of dityramine cellulose obtained from SKP-DAC (DO = 39 %).

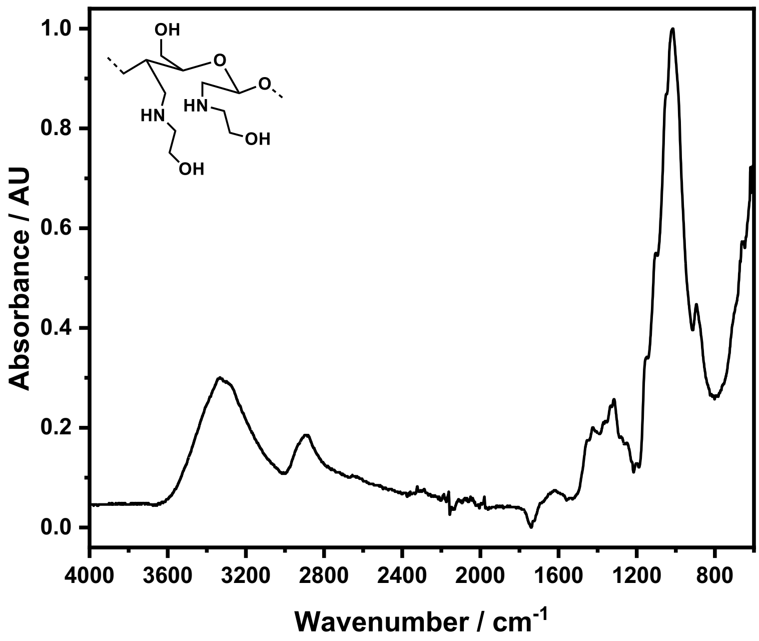

Figure S52. Normalized Fourier-transform infrared spectra of diethanolamine cellulose obtained from SKP-DAC (DO = 39 %).

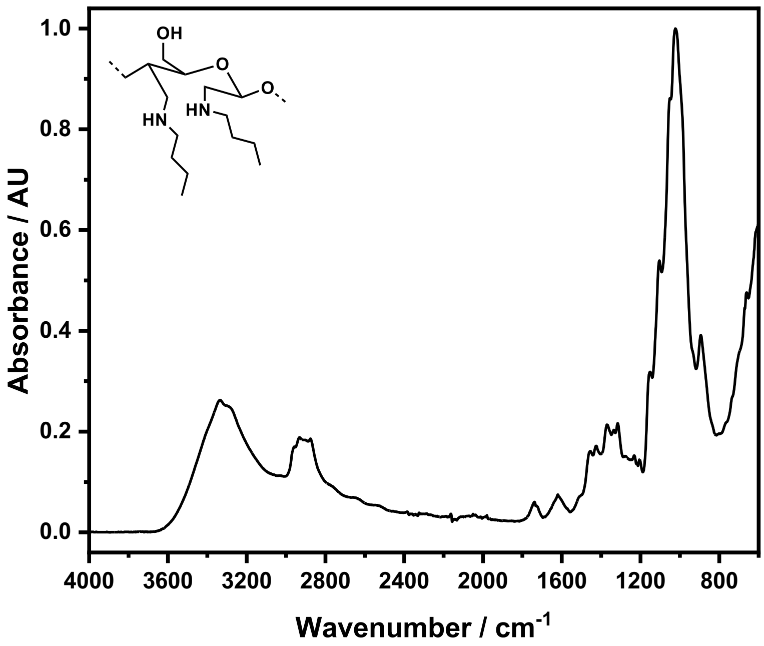

Figure S53. Normalized Fourier-transform infrared spectra of dibutylamine cellulose obtained from SKP-DAC (DO = 39 %).

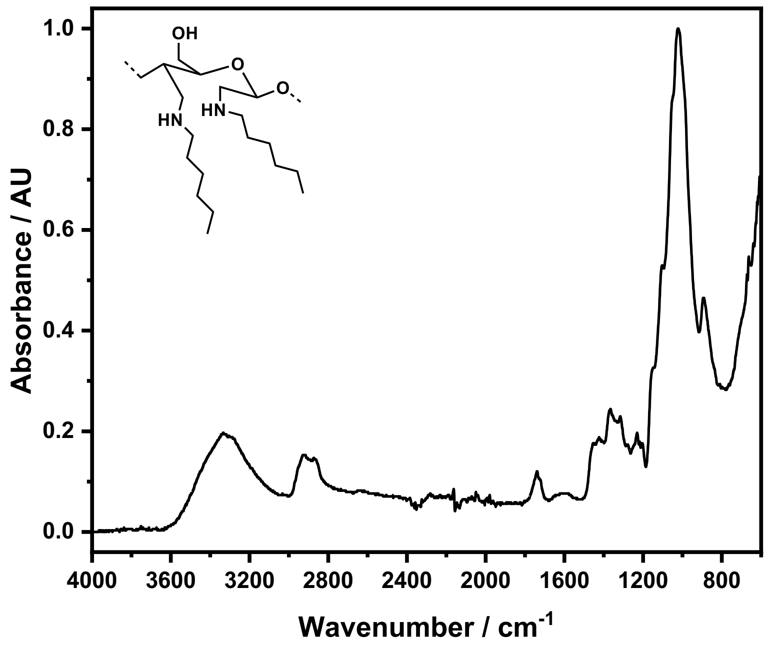

Figure S54. Normalized Fourier-transform infrared spectra of dihexylamine cellulose obtained from SKP-DAC (DO = 39 %).

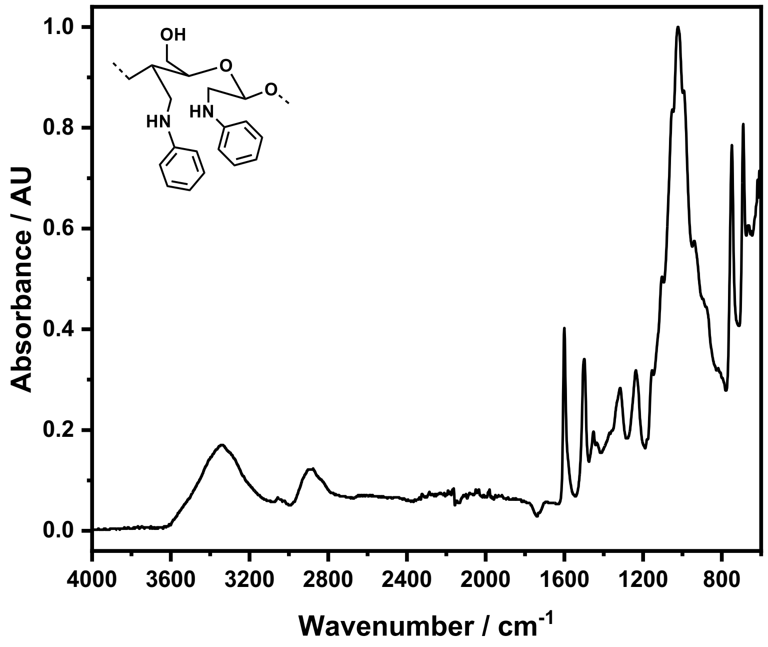

Figure S55. Normalized Fourier-transform infrared spectra of dianiline cellulose obtained from SKP-DAC (DO = 39 %).

5) GPC/MALLS-RI data

5.1) Diamine celluloses obtained from microcrystalline cellulose (MCC, Avicel® PH-101) (DO = 8 %)

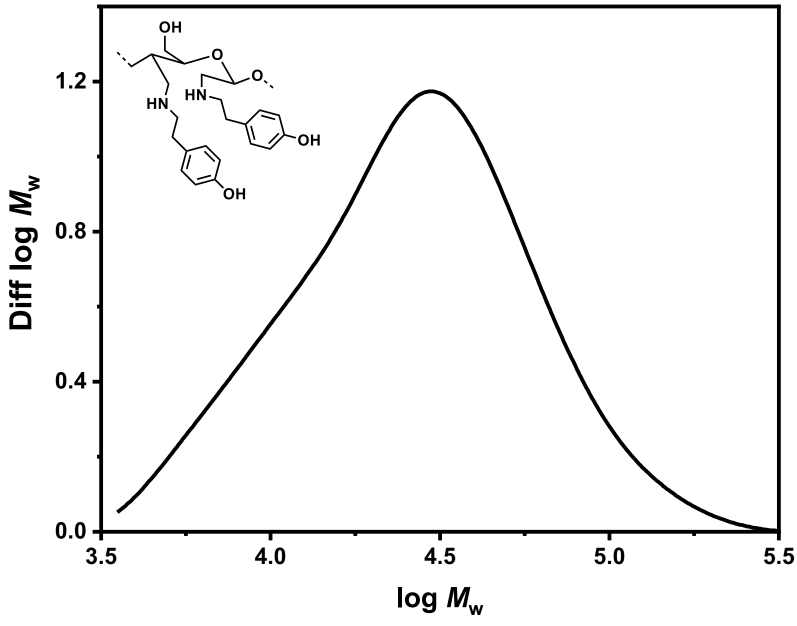

Figure S56. Molecular weight distribution of dityramine cellulose obtained from MCC-DAC (DO = 8 %).

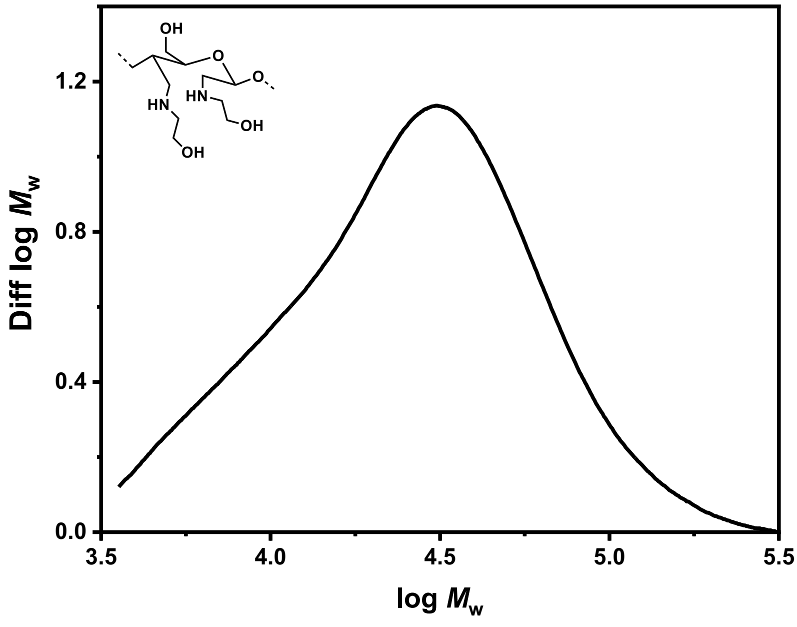

Figure S57. Molecular weight distribution of ditethanolamine cellulose obtained from MCC-DAC (DO = 8 %).

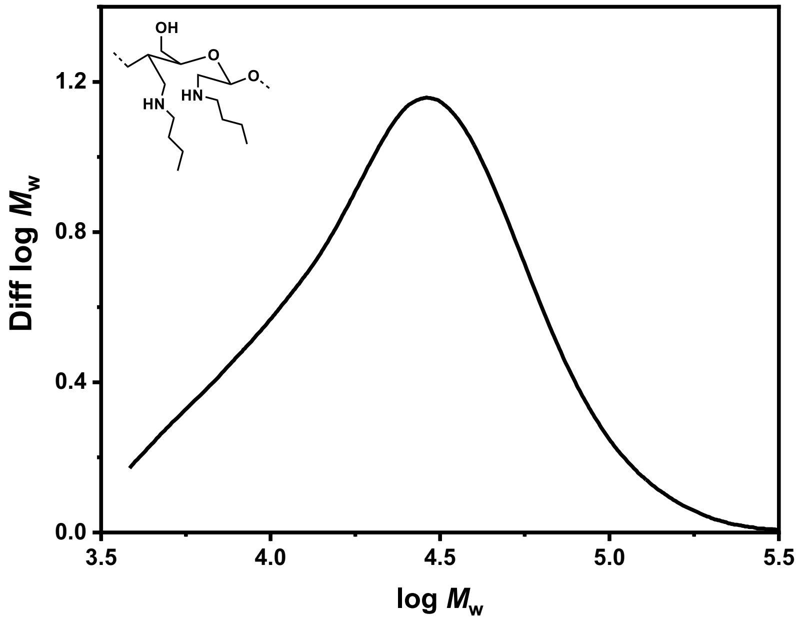

Figure S58. Molecular weight distribution of dibutylamine cellulose obtained from MCC-DAC (DO = 8 %).

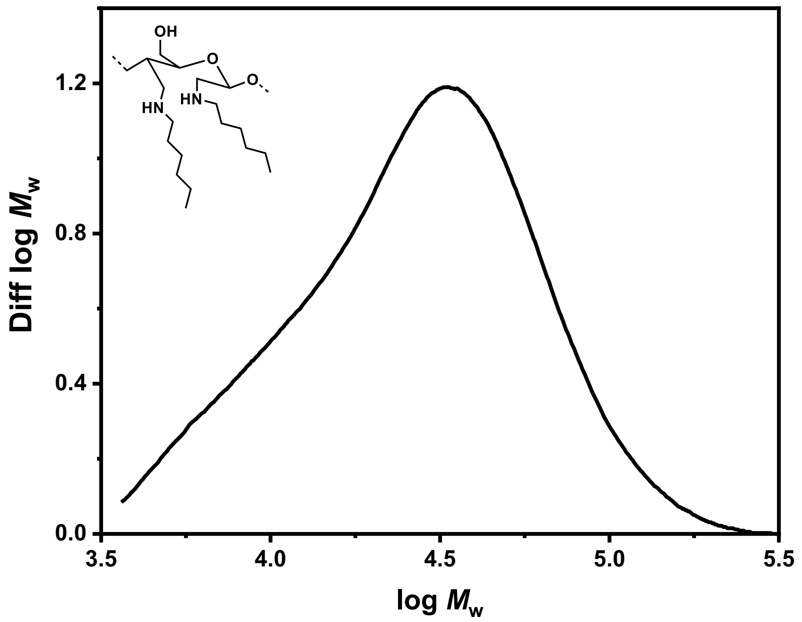

Figure S59. Molecular weight distribution of dihexylamine cellulose obtained from MCC-DAC (DO = 8 %).

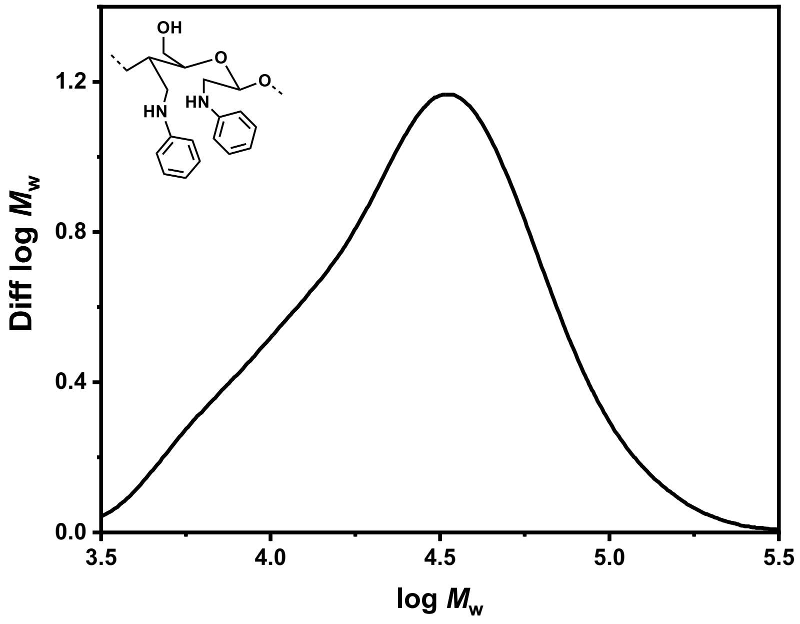

Figure S60. Molecular weight distribution of dianiline cellulose obtained from MCC-DAC (DO = 8 %).

5.2) Diamine celluloses obtained from partially oxidized softwood kraft pulp (DO = 39 %)

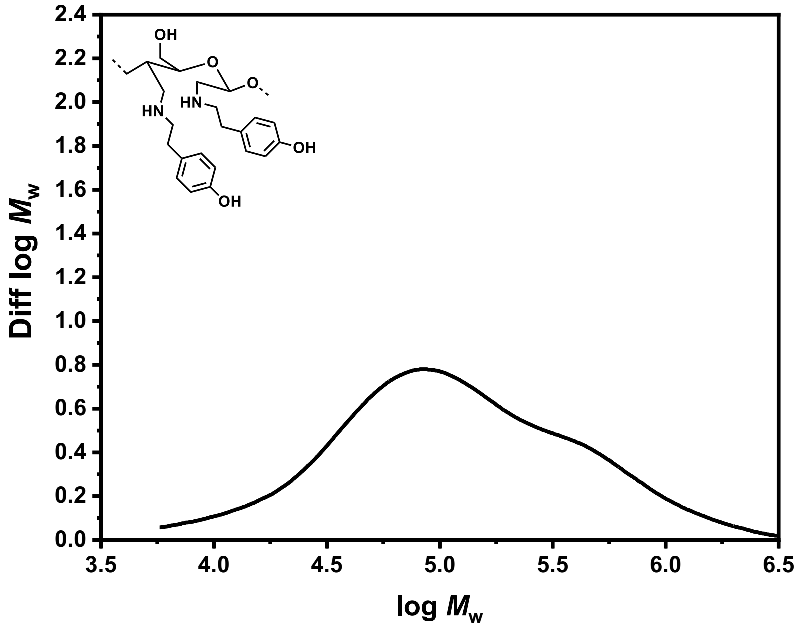

Figure S61. Molecular weight distribution of dityramine cellulose obtained from SKP-DAC (DO = 39 %).

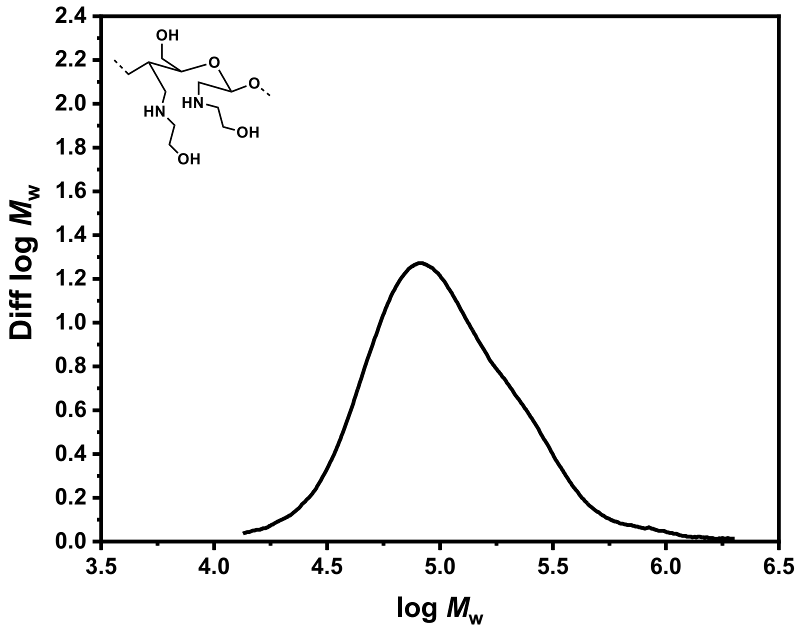

Figure S62. Molecular weight distribution of diethanolamine cellulose obtained from SKP-DAC (DO = 39 %).

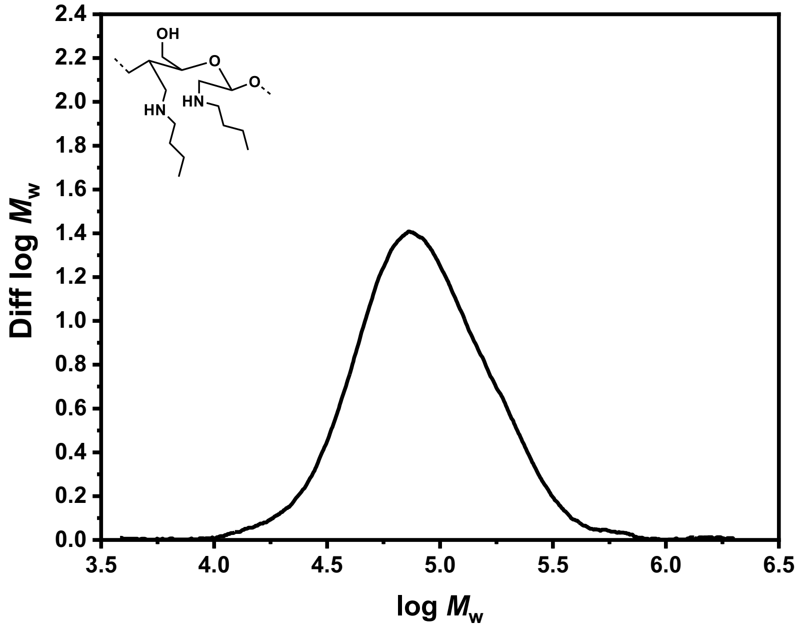

Figure S63. Molecular weight distribution of dibutylamine cellulose obtained from SKP-DAC (DO = 39 %).

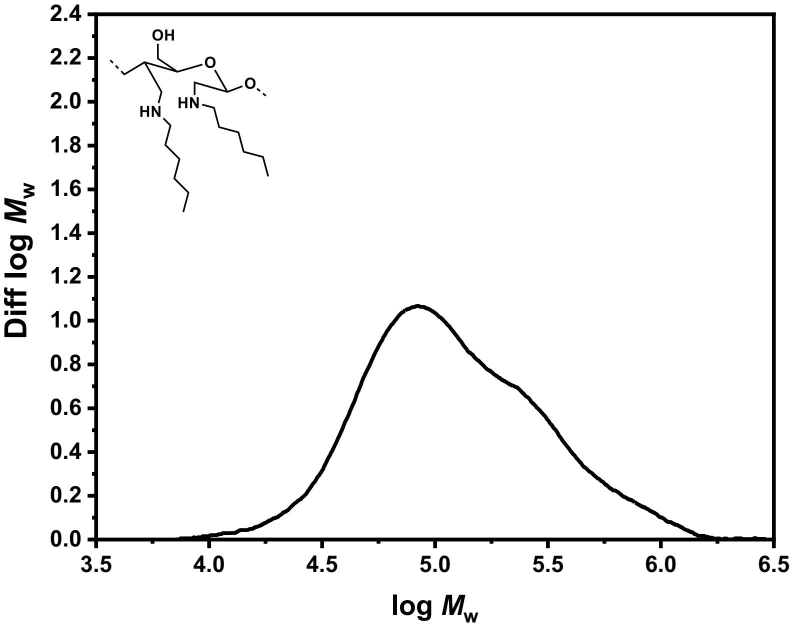

Figure S64. Molecular weight distribution of dihexylamine cellulose obtained from SKP-DAC (DO = 39 %).

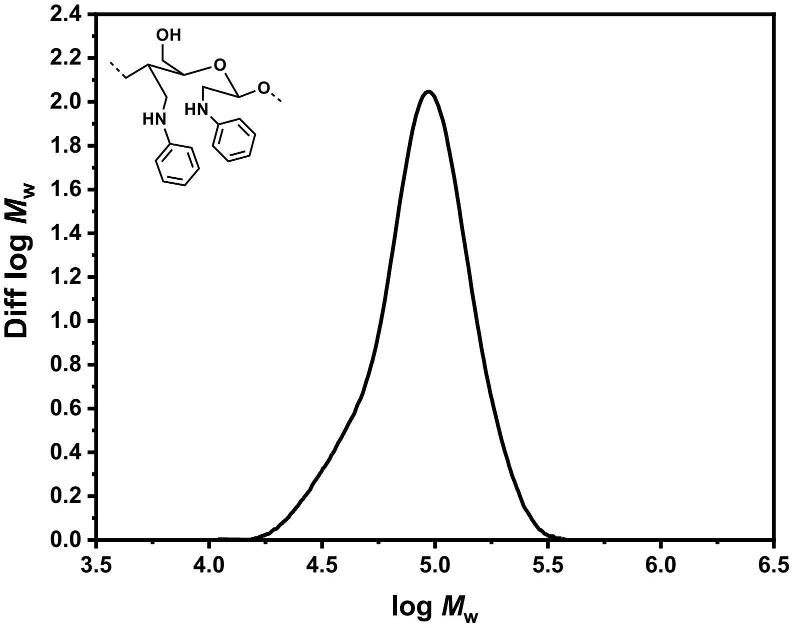

Figure S65. Molecular weight distribution of dianiline cellulose obtained from SKP-DAC (DO = 39 %).

6) Derivation of the formula to calculate the aldehyde content from titration data

Formula 1:

$$CHO\ [\%] = \frac{V_{NaOH} \cdot [NaOH] \cdot M \cdot V_0}{2 \cdot m_0 \cdot V_1} \cdot 100$$

to calculate the aldehyde content CHO can be derived from the formed hydrochloric acid after quantitative reaction with hydroxylamine hydrochloride. The remaining aldehyde groups correspond to the proportion of oxidized anhydroglucose units $n_{oxidized}$ in the polymer backbone:

$$CHO\ [\%] = \frac{n_{oxidized}}{n_0} \cdot 100$$

The moles of oxidized anhydroglucose units which were not converted to the diamine after reductive amination can be calculated from the consumed sodium hydroxide solution when neutralizing the formed hydrochloric acid. The oximation of each remaining aldehyde group forms one mole of hydrochloric acid. And since every oxidized anhydroglucose unit contains two carbonyl groups, the moles of remaining oxidized anhydroglucose are:

$$n_{oxidized} = \frac{n_{NaOH}}{2} = \frac{V_{NaOH} \cdot [NaOH]}{2}$$

Here, $[NaOH]$ is the NaOH concentration and V_{NaOH} the volume of consumed NaOH. The total moles of the sample can be calculated from

$$n_0 = \frac{m_0}{M} \cdot \frac{V_1}{V_0}$$

where m_0 is the weight of the freeze-dried sample, M is the molecular weight average of the modified and unoxidized anhydroglucose units (which depend of the introduced amine and the degree of oxidation). For the molecular weight M, we assumed that the molecular weight of the diamino cellulose consists only of aminated units and unoxidized anhydroglucose units:

$$M = \frac{DO}{100} \cdot M_{DA} + \left(\frac{100 - DO}{100}\right) \cdot M_{AGU}$$

DO is the degree of oxidation of the dialdehyde cellulose used (in our study 39 % or 8 %), M_{DA} the molecular weight of the diaminated anhydroglucose unit and M_{AGU} the molecular weight of the unoxidized anhydroglucose unit (162.14 g mol^{-1}).

Therefore,

$$CHO\ [\%] = \frac{\frac{V_{NaOH} \cdot [NaOH]}{2}}{\frac{m_0}{M} \cdot \frac{V_1}{V_0}} \cdot 100$$

$$\Rightarrow \frac{V_{NaOH} \cdot [NaOH] \cdot M \cdot V_0}{2 \cdot m_0 \cdot V_1} \cdot 100$$

7) Derivation of the formula to calculate the degree of substitution from elemental analysis data

Equation 3:

$$DS\ [\%] = \frac{100 \cdot N\% \cdot M_{AGU}}{-N\% \cdot M_{DA} + N\% \cdot M_{AGU} + 2800}$$

can be derived from the determined nitrogen content $N\%$ in the analyzed cellulose derivative. The degree of substitution DS corresponds to the proportion of modified/deaminated anhydroglucose units n_{DA} in the cellulose backbone (sum of modified anhydroglucose units n_{DA} and unmodified units n_{AGU}):

$$DS\ [\%] = \frac{n_{DA}}{n} \cdot 100 = \frac{n_{DA}}{n_{DA} + n_{AGU}} \cdot 100$$

Note that

$$n_{DA} = \frac{N\%}{100 \cdot 2 \cdot 14} = \frac{N\%}{100 \cdot 28} = \frac{N\%}{2800}$$

where $N\%$ is the determined nitrogen content in percent. Every nitrogen atom has an atomic weight of 14 u, and every oxidized anhydroglucose unit is aminated twice. The moles of unoxidized anhydroglucose units in the polymer backbone correspond to

$$n_{AGU} = \frac{m_{AGU}}{M_{AGU}} = \frac{(1 - m_{DA})}{M_{AGU}} = \frac{(1 - M_{DA} \cdot n_{DA})}{M_{AGU}} = \frac{\left(1 - M_{DA} \cdot \frac{N\%}{2800}\right)}{M_{AGU}} = \frac{\left(\frac{-N\% \cdot M_{DA}}{2800} + 1\right)}{M_{AGU}}$$

where m_{AGU} is the mass fraction of the unmodified anhydroglucose units, M_{AGU} the molecular weight of the unmodified anhydroglucose units (162.14 g mol^{-1}), m_{DA} the mass fraction of the deaminated anhydroglucose units, M_{DA} the molecular weight of the deaminated anhydroglucose units (which depends of the introduced amine) and n_{DA} again the moles of deaminated anhydrocluose units.

Therefore,

$$DS\ [\%] = \frac{\frac{N\%}{2800}}{\frac{N\%}{2800} + \frac{\left(\frac{-M_{DA} \cdot N\%}{2800} + 1\right)}{M_{AGU}}} \cdot 100 = \frac{\frac{N\%}{2800} \cdot 100}{\frac{N\%}{2800} + \frac{\left(\frac{-M_{DA} \cdot N\%}{2800} + 1\right)}{M_{AGU}}}$$

$$= \frac{\frac{N\%}{2800} \cdot 100}{\frac{N\% \cdot M_{AGU}}{2800 \cdot M_{AGU}} + \frac{\left(\frac{-M_{DA} \cdot N\%}{2800} + 1\right) \cdot 2800}{M_{AGU} \cdot 2800}}$$

$$= \frac{\frac{N\%}{2800} \cdot 100}{\frac{N\% \cdot M_{AGU}}{M_{AGU} \cdot 2800} + \frac{(-M_{DA} \cdot N\% + 2800)}{M_{AGU} \cdot 2800}} = \frac{\frac{N\%}{2800} \cdot 100}{\frac{N\% \cdot M_{AGU} + (-M_{DA} \cdot N\% + 2800)}{M_{AGU} \cdot 2800}}$$

$$= \frac{N\% \cdot 100}{2800} \cdot \frac{M_{AGU} \cdot 2800}{N\% \cdot M_{AGU} + (-M_{DA} \cdot N\% + 2800)}$$

$$\Rightarrow \frac{100 \cdot N\% \cdot M_{AGU}}{-N\% \cdot M_{DA} + N\% \cdot M_{AGU} + 2800}$$

References

(1) Jusner, P.; Bacher, M.; Simon, J.; Bausch, F.; Khaliliyan, H.; Schiehser, S.; Sumerskii, I.; Schwaiger, E.; Potthast, A.; Rosenau, T. Analyzing the Effects of Thermal Stress on Insulator Papers by Solid-State 13C NMR Spectroscopy. *Cellulose* **2021**, *0123456789*. https://doi.org/10.1007/s10570-021-04338-z.

(2) Sundheq, A.; Sundherg, K.; Lillandt, C.; Holmhom, B. Determination of Hemicelluloses and Pectins in Wood and Pulp Fibres by Acid Methanolysis and Gas Chromatography. *Nord. Pulp Pap. Res. J.* **1996**, *11* (4), 216–219. https://doi.org/doi:10.3183/npprj-1996-11-04-p216-219.
